# Strategies for de-implementation of low-value care—a scoping review

**DOI:** 10.1186/s13012-022-01247-y

**Published:** 2022-10-27

**Authors:** Sara Ingvarsson, Henna Hasson, Ulrica von Thiele Schwarz, Per Nilsen, Byron J. Powell, Clara Lindberg, Hanna Augustsson

**Affiliations:** 1 Procome Research Group, Medical Management Centre, Department of Learning, Informatics, Management and Ethics, Karolinska Institutet, Karolinska, Sweden; 2 Unit for implementation and evaluation, Center for Epidemiology and Community Medicine (CES), Stockholm Region, Stockholm, Sweden; 3 School of Health, Care and Social Welfare, Mälardalen University, Västerås, Sweden; 4 Department of Health, Medicine and Caring Sciences, Division of Public Health, Linköping University, Linköping, Sweden; 5 Center for Mental Health Services Research, Brown School, Washington University in St. Louis, St. Louis, MO USA; 6 Center for Dissemination and Implementation, Institute for Public Health, Washington University in St. Louis, St. Louis, MO USA; 7 Division of Infectious Diseases, John T. Milliken Department of Medicine, School of Medicine, Washington University in St. Louis, St. Louis, MO USA

**Keywords:** De-implementation, Low-value care, Strategies, Scoping review

## Abstract

**Background:**

The use of low-value care (LVC) is a persistent problem that calls for knowledge about strategies for de-implementation. However, studies are dispersed across many clinical fields, and there is no overview of strategies that can be used to support the de-implementation of LVC. The extent to which strategies used for implementation are also used in de-implementing LVC is unknown. The aim of this scoping review is to (1) identify strategies for the de-implementation of LVC described in the scientific literature and (2) compare de-implementation strategies to implementation strategies as specified in the Expert Recommendation for Implementing Change (ERIC) and strategies added by Perry et al.

**Method:**

A scoping review was conducted according to recommendations outlined by Arksey and O’Malley. Four scientific databases were searched, relevant articles were snowball searched, and the journal *Implementation Science* was searched manually for peer-reviewed journal articles in English. Articles were included if they were empirical studies of strategies designed to reduce the use of LVC. Two reviewers conducted all abstract and full-text reviews, and conflicting decisions were discussed until consensus was reached. Data were charted using a piloted data-charting form. The strategies were first coded inductively and then mapped onto the ERIC compilation of implementation strategies.

**Results:**

The scoping review identified a total of 71 unique de-implementation strategies described in the literature. Of these, 62 strategies could be mapped onto ERIC strategies, and four strategies onto one added category. Half (50%) of the 73 ERIC implementation strategies were used for de-implementation purposes. Five identified de-implementation strategies could not be mapped onto any of the existing strategies in ERIC.

**Conclusions:**

Similar strategies are used for de-implementation and implementation. However, only a half of the implementation strategies included in the ERIC compilation were represented in the de-implementation studies, which may imply that some strategies are being underused or that they are not applicable for de-implementation purposes. The strategies *assess and redesign workflow* (a strategy previously suggested to be added to ERIC), *accountability tool,* and *communication tool* (unique new strategies for de-implementation) could complement the existing ERIC compilation when used for de-implementation purposes.

**Supplementary Information:**

The online version contains supplementary material available at 10.1186/s13012-022-01247-y.

Contributions to the literature

- The study contributes a synthesis of strategies that can be used to support the de-implementation of LVC identified in the peer-reviewed literature.
- The study surfaces similarities and differences between de-implementation and implementation strategies.
- Strategies that could be beneficial to add to the existing Expert Recommendation for Implementing Change (ERIC) compilation when used for de-implementation purposes are proposed.
- The scoping review identifies gaps in the current knowledge base, and suggestions for future research are offered.

## Background

Recognition is growing with regard to the importance of reducing low-value care (LVC), i.e., “care that is unlikely to benefit the patient given the harms, cost, available alternatives, or preferences of the patient” [1]. Common examples of LVC are non-indicated antibiotics, unnecessary imaging, potentially inappropriate medications for the elderly and unnecessary lab tests [2]. LVC has become a pervasive problem in health care in high-income countries [1, 3–5], with around 30% of care estimated to be of low value [6]. Furthermore, estimations show that about 7% of the care considered to be best practice 1 year becomes LVC the next [7]. Thus, the rapid development of new practices (e.g., diagnostics and treatments) not only calls for continuous implementation of new evidence but also requires the de-implementation of LVC. De-implementation entails a structured process with the purpose of reducing or ceasing the use of LVC [8].

Similar to implementing evidence-based interventions, de-implementing LVC is a complex process influenced by multilevel factors [9]. Determinants of LVC use and de-implementation include various patient characteristics such as age, gender, ethnicity, and socio-economic factors, although there are no consistent patterns as to their positive or negative influence on LVC [2]. For instance, older age is usually associated with use of LVC [10–12], but some studies have linked younger age with higher LVC use [13, 14]. Patients’ health conditions—e.g., the severity of illness and characteristics of the disease [15, 16]—often contribute to use of LVC. Patient expectations, e.g., patients who request non-indicated prescriptions, also tend to increase the occurrence of LVC [17, 18]. Health professionals’ characteristics are also associated with LVC use. As with patient characteristics, the results are inconsistent regarding professionals’ age, gender, and length of experience [2]. However, a lack of or inadequate training has consistently been linked to use of LVC [17, 19]. Professionals’ knowledge of LVC contributes to and protects against the use of LVC [2]. For example, a lack of knowledge about cost-effectiveness [20] and poor cost-awareness [21] are associated with use of LVC. Professionals’ expectations and attitudes also influence LVC use, e.g., their fear of malpractice and desire to meet patient requests [2]. Interaction between patients and professionals can also impact the use of LVC, e.g., communication about unnecessary antibiotics or tests [22].

Determinants of LVC also exist at the contextual level [2]. Inner context, including setting characteristics, care processes (e.g., lack of care continuity), perceived lack of time and time pressure when performing work tasks, accessibility of decision support, staffing levels, and composition and organizational incentives for LVC use have been identified [2]. Outer context determinants have included location of the health care organization (e.g., metropolitan, urban, suburban, or rural), financing and financial incentives (e.g., fee-for-service funding), policy and political support, and marketing initiatives such as promotion of screening directed to the population and direct-to-consumer advertising about drugs or treatments.

Yet, knowing the potential determinants of LVC is not sufficient for changing them. Strategies to address determinants constitute the “how-to” component of changing practice. Strategies are methods and techniques to facilitate implementation of evidence-based practices and/or de-implementation of LVC [23]. Some implementation strategies are likely to be applicable for de-implementation, while other strategies may be unique or more applicable for de-implementation [24]. However, the evidence for de-implementation strategies is dispersed across multiple clinical fields, which makes it difficult to document and survey findings [2]. Studies investigating strategies to reduce LVC have been published in a broad range of journals, typically within specific clinical and medical care areas, from microbiological research on antimicrobial resistance to potentially inappropriate medication for the elderly [2]. Studies on strategies for de-implementation have also focused on specific LVC within fields such as nursing [25], low-value blood management techniques in primary hip and knee arthroplasty [26], pharmacological prescriptions [27], and cancer [28]. An exception is a systematic review of de-implementation strategies covering a wide range of clinical areas [29] that found promising results for clinical decision support and performance feedback, concluding that multicomponent strategies addressing both clinicians and patients had the greatest potential for reducing LVC. Another review [28] focused specifically on cancer care similarly found that most de-implementation strategies were multifaceted. The most widely used strategies were audit and feedback, use of clinical champions, educating clinicians through developing and disseminating guidelines, and decision-support tools. Integrating a clinical decision-support tool in the electronic health record system for real-time alerts was the most effective strategy.

The de-implementation field suffers from a lack of established nomenclature for how strategies to de-implement LVC are named, defined, and organized. This makes it difficult to compare strategies across studies, hindering an accumulation of a generalizable body of knowledge related to effectively de-implementing LVC. A notable exception is a recent study in which researchers used the behavior-change techniques taxonomy to categorize de-implementation categories based on data from three systematic reviews [30]. They also compared de-implementation strategies to implementation strategies and showed that *behavior substitution*, *monitoring of behavior by others without feedback,* and *restructuring social environment* were more frequently used in de-implementation efforts than in implementation. However, this study was limited to strategies aiming to change individual clinicians’ behavior and did not cover strategies on a system or policy level.

In contrast, researchers studying implementation have developed taxonomies to guide the identification, selection, and reporting of strategies, thus making it easier to compare strategies across studies [31–34]. One such taxonomy is the ERIC compilation [31], which consists of 73 discrete implementation strategies belonging to nine categories [35]. ERIC has been widely used in implementation science and is useful in evaluations of implementation strategies [36–39]. However, it is unknown whether the same types of strategies are also used in de-implementation. It is likely that some of the 73 strategies and nine categories of the ERIC compilation are also relevant for de-implementation purposes. Still, findings from a recent study [30] using behavior change taxonomy suggest that this might not be the case, since de-implementation differs in the primary behavior-change techniques utilized. Thus, it is necessary to investigate to what extent de-implementation and implementation strategies are the same.

This study addresses two important knowledge gaps in the literature. First, due to the dispersion of studies on strategies for de-implementation across many clinical fields, no overview of the strategies that can be used to support LVC de-implementation exists. This fragmentation inhibits the systematic development of knowledge about effective strategies for the de-implementation of LVC. Second, it is unknown whether and to what extent implementation strategies are also applicable for de-implementing LVC. Addressing these key knowledge gaps, the aim of this review is to evaluate the scope of the literature to (1) identify strategies for the de-implementation of LVC described in the scientific literature and (2) compare de-implementation strategies to implementation strategies as specified in ERIC and strategies added by Perry et al. [36].

## Methods

### Design

We conducted a scoping review based on the steps outlined by Arksey and O´Malley [40] and reported the review in accordance with the Preferred Reporting Items for Systematic Reviews and Meta-Analyses Extension for Scoping Reviews (PRISMA-ScR) checklist [41] (Additional file 1).

### Protocol and registration

We described the method in a previously published study protocol [42].

### Eligibility criteria

We included English-language articles describing empirical studies published in peer-reviewed journals. Using the PCC mnemonic (Population, Concept, Context) recommended for scoping reviews [43], we specified that the content of the articles should focus on interventions or strategies aiming to reduce LVC within health care (context). Health care was defined as all types of primary, hospital (secondary), community, and mental health care. We did not specify a population since we were interested in strategies for reducing LVC in health care in general. All study designs were considered. We included LVC practices as defined based on a published guideline or recommendation, as stated in the article. All eligibility criteria are reported in Table 1.

**Table 1:** Eligibility criteria for inclusion

1. English language	
2. Published between January 2013 and September 2021	
3. Published in a peer-reviewed journal	
4. Empirical study	
5. Population: not specified	
6. Concept: Strategies for reducing the use of LVC (NB. The studies needed to refer to a recommendation [e.g., choosing wisely] or a guideline [e.g., clinical guidelines] stating that the practice is not recommended)	
7. Context: Health care setting (including primary care, hospital care, community care, and mental health)	

### Information sources

We searched four electronic databases: MEDLINE, Embase, CINAHL, and Web of Science. We also searched the journal *Implementation Science* manually and searched reference lists in relevant articles for additional papers.

### Search

We identified keywords for the search by means of an extensive discussion among ourselves, a review outlining potential terminology for de-implementation [44], an inspection of key articles, and discussions with representatives from the Swedish Agency for Health Technology Assessment and Assessment of Social Services. We identified 18 key articles used to inform the search strategy. In collaboration with the Karolinska Institutet library, we defined, tested, and refined a search strategy three times to ensure that it was broad enough to capture the 18 key articles and sufficiently discriminant to generate a reasonable number of articles. We conducted the first search in June 2018, and the search strategy included articles published from 2013 to June 2018. We chose to limit our time frame to make the number of papers feasible and chose 2013 as a starting point due to comparatively higher number of articles published during these years. We conducted a second search in September 2021 because a considerable number of studies had been published during the finalization of the review. The second search included articles published between June 2018 and September 2021. Table 2 shows the search strategy for Web of Science for the first search. All other strategies, including the second search, can be found as an attachment (Additional file 2). The only difference between the first and second search was the focus solely on strategies in the second one.

**Table 2:** Search strategy used in Web of Science

Field labels	
• TS/Topic = title, abstract, author keywords and Keywords Plus	
• NEAR/x = within x words, regardless of order	
• * = truncation of word for alternate endings	
#1 TOPIC: (((abandon* OR contradict* OR deadopt* OR ”de-adopt*” OR disadopt* OR ”dis-adopt*” OR decommission* OR ”de-commission*” OR deimplement* OR ”de-implement*” OR delist* OR ”de-list*” OR disinvest* OR ”dis-invest” OR deprescript* OR deprescrib* OR divest* OR inapprop* OR ineffective* OR ”low-value” OR obsole* OR outmoded OR overuse OR reallocate* OR reassess* OR ”re-assess*” OR refute* OR refuting OR ”re-invest*” OR ”medical revers*” OR supersed* OR unlearn*) NEAR/3 (care OR clinic* OR device* OR drug OR drugs OR evidence* OR health OR healthcare OR medical OR medication* OR prescrib* OR procedur* OR technolog* OR therap* OR treat*)))	
#2 (((chang* or discontinu* or ”dis-continu*” or decreas* or declin* or drop or reduc* or withdraw*) NEAR/1 ("use" or practice) NEAR/3 (care or clinic* or device* or drug or drugs or evidence* or health or healthcare or medical or medication* or prescrib* or procedur* or technolog* or therap* or treat*)))	
#3 TS=("choosing wisely" or "priority setting") AND TS=(care or clinic* or device* or drug or drugs or evidence* or health or healthcare or medical or medication* or prescrib* or procedur* or technolog* or therap* or treat*)	
#4 #3 OR #2 OR #1	
#5 ((abandon* OR contradict* OR deadopt* OR ”de-adopt*” OR disadopt* OR ”dis-adopt*”	
OR decommission* OR ”de-commission*” OR deimplement* OR ”deimplement*” OR delist* OR ”de-list*” OR disinvest* OR ”dis-invest” OR discontinu* OR ”dis-continu*” OR deprescipt* OR deprescrib* OR divest* OR inapprop* OR ineffective* OR ”low-value” OR obsole* OR outmoded OR overuse OR reallocate* OR reassess* OR ”re-assess*” OR refute* OR refuting OR ”re-invest*” OR ”medical revers*” OR supersed* OR unlearn* OR withdraw*) NEAR/3 (factor* OR barrier* OR engag* OR ”evidencebased” OR facilitat* OR determinant* OR predict* OR model* OR framework* OR intervent* OR policy OR policies OR ”practice pattern*” OR program* OR strateg* OR tool*))	
#6 #5 AND #4	
Refined by: LANGUAGES: ( ENGLISH )	

### Selection of sources

We imported all articles to Rayyan [45] to screen. In total, eight people (authors 1, 2, 6, and 7 and four research assistants) were involved in the process. In the first step, authors 1 and 7, along with two research assistants, reviewed a sample of 40 abstracts independently to test the inclusion and exclusion criteria. They discussed differences in judgements and adjusted the criteria based on the discussion to ensure a consistent assessment among screeners. Authors 1, 2, and 7 and two research assistants then screened the abstracts independently. All abstracts were screened by two screeners, who discussed any inconsistencies. If these were not resolved, all authors discussed the inconsistencies. In the next step, two reviewers (authors 1, 6, and 7 and two other research assistants) assessed all full-text articles and applied the same process for resolving inconsistencies. Corresponding authors were contacted for clarification if the screeners were uncertain whether an article fulfilled the criteria for a formal guideline used to define LVC.

### Data charting

Authors 1 and 6 developed a data-charting table in Excel and tested it on five articles. The authors did not find any inconsistencies between them. One author [6] continued with the data extraction of all articles from the first search and consulted the rest of the authors when needed. Two research assistants who had been trained by the first author conducted extraction from the second search. The first author reviewed all the extracted data.

### Data items

The data that were charted included aim and research question; setting; study design; data collection method; type of evaluation (e.g., effectiveness/efficacy, process evaluation, or cost effectiveness); type of LVC; guideline used to define LVC; single or multicomponent strategy; and activities included in the strategy (as described in the original article).

### Synthesis of results

The data were first coded inductively regarding the type of strategy described in the study. Inductive coding made it possible to capture a detailed description of the strategies and was chosen since no existing taxonomy for de-implementation strategies exists. The coding was performed in pairs. Strategies were excluded if they could not be understood as a result of limited reporting. All inductive codes were then compared and mapped onto ERIC strategies, including three new strategies suggested by Perry et al. [36]. Authors 1 and 5 performed the mapping onto ERIC, and authors 1 and 7 solved inconsistencies. Author 2 checked the samples of these codes to make sure the transfer from inductive codes to ERIC categories did not change the intent of the strategy described in the studies. All inductive codes and their ERIC mapping can be found in Additional file 3. The results of the mapping were then summarized based on the nine categories of ERIC strategies outlined by Waltz et al. [35].

## Results

**Fig. 1:**
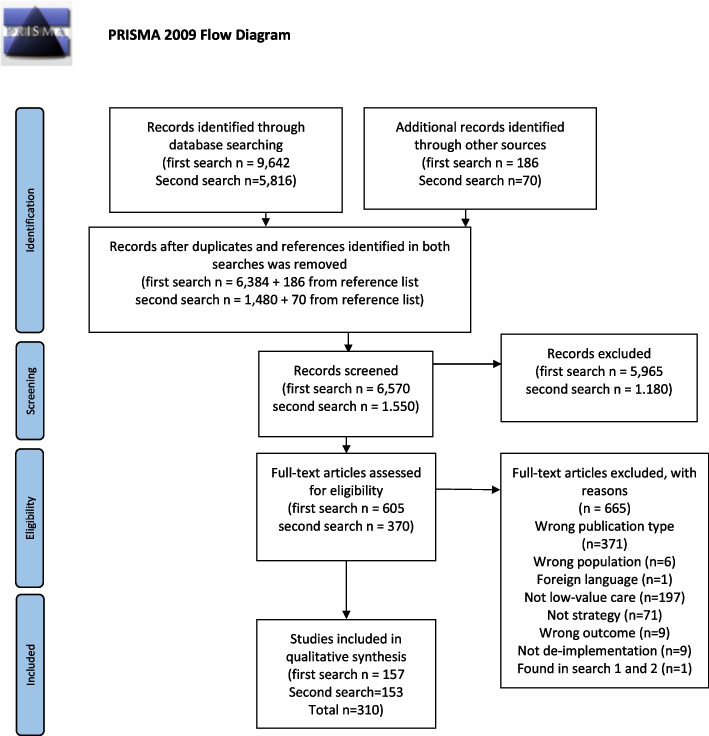
PRISMA flow diagram

The first search resulted in 9642 citations and the second search in an additional 5816 (Fig. 1). An additional 186 citations in the first search and 70 in the second search were identified through searches in reference lists. Removing duplicates resulted in 6570 unique citations from the first search and 1,550 from the second search for abstract screening. After abstract screening, 605 citations from the first search and 370 from the second search were included for full-text review. Following full-text screening, 310 citations remained and were included in the study.

### Study characteristics

The 310 studies originated from 38 countries, with almost half conducted in the USA (*n* = 136). The most frequent type of LVC was non-indicated antibiotics (*n* = 84), followed by potentially inappropriate medication for the elderly (*n* = 73), imaging (*n* = 41), and lab tests (*n* = 31). The most common study setting was hospitals (*n* = 153), followed by primary care (*n* = 87). Only three studies focused on influencing on a systems level whereas the rest of the studies targeted individual health care professionals.

Of the 310 studies, 279 were based on quantitative methods, 25 were mixed methods, and 10 were qualitative. The most common study design was a pre–post study design (*n* = 147), followed by quasi-experimental study design (*n* = 66) and randomized controlled trial (*n* = 39). The most frequent type of evaluation was efficacy/effectiveness (*n* = 260), followed by process evaluation (*n* = 21). Of the 310 studies, 217 used multicomponent strategies to reduce LVC, and 93 used single-component strategies.

### Identified de-implementation strategies

The inductive coding yielded 71 unique strategies, of which 62 could be mapped onto ERIC strategies. A total of 36 of the 73 ERIC strategies were covered. Four of the identified strategies could be mapped onto one of the additional strategies suggested by Perry et al.: *assess and redesign workflow* [36]. Five of the identified strategies could not be mapped onto the ERIC compilation or the suggested additions from Perry et al. [36].

The de-implementation strategies used most commonly were related to the ERIC categories *training and education of stakeholders*, *use of evaluative and iterative strategies*, and *support of clinicians* (Table 3).

**Table 3:** Number and percentages of the identified de-implementation strategies

Categories of strategies in ERIC	Strategy in ERIC	Number of studies	Percentage of total no. of studies
**Train and educate stakeholders**	Conduct ongoing training	0	0%
	Provide ongoing consultation	0	0%
	Develop educational materials	106	34%
	Make training dynamic	85	27%
	Distribute educational materials	59	19%
	Use train-the-trainer strategies	3	1%
	Conduct educational meetings	64	21%
	Conduct educational outreach visits	30	10%
	Create a learning collaborative	3	1%
	Shadow other experts	0	0%
	Work with educational institutions	0	0%
	**Sum strategies**	**350**^a^	
**Use evaluative and iterative strategies**	Assess for readiness and identify barriers and facilitators	10	3%
	Audit and provide feedback	89	29%
	Purposively reexamine the implementation	0	0%
	Develop and implement tools for quality monitoring	3	1%
	Develop and organize quality monitoring systems	86	28%
	Develop a formal implementation blueprint	0	0%
	Conduct local needs assessment	10	3%
	Stage implementation scale up	2	1%
	Obtain and use patients/consumers and family feedback	0	0%
	Conduct cyclical small tests of change	6	2%
	**Sum strategies**	**206**	
**Support clinicians**	Facilitate relay of clinical data to providers	3	1%
	Remind clinicians	92	30%
	Develop resource sharing agreements	0	0%
	Revise professional roles	2	1%
	Create new clinical teams	3	1%
	**Sum strategies**	**100**	
**Develop stakeholder interrelationships**	Identify and prepare champions	15	5%
	Organize clinician implementation team meetings	1	1%
	Recruit, designate, and train for leadership	0	0%
	Inform local opinion leaders	0	0%
	Build a coalition	0	0%
	Obtain formal commitment	9	3%
	Identify early adopters	0	0%
	Conduct local consensus discussions	1	1%
	Capture and share local knowledge	0	0%
	Use advisory boards and workgroups	28	9%
	Use an implementation advisor	0	0%
	Model and simulate change	0	0%
	Visit other sites	0	0%
	Involve executive boards	0	0%
	Develop an implementation glossary	0	0%
	Develop academic partnerships	0	0%
	Promote network weaving	0	0%
	**Sum strategies**	**54**	
**Change infrastructure**	Mandate change	2	1%
	Change record systems	1	1%
	Change physical structure and equipment	38	12%
	Create or change credentialing and/or licensure standards	0	0%
	Change service sites	0	0%
	Change accreditation or membership requirements	0	0%
	Start a dissemination organization	1	1%
	Change liability laws	0	0%
	**Sum strategies**	**40**	
**Utilize financial strategies**	Fund and contract for clinical innovation	0	0%
	Access new founding	0	0%
	Place innovation on fee for service lists/formularies	0	0%
	Alter incentive/allowance structures	6	2%
	Make billing easier	0	0%
	Alter patient/consumer fees	3	1%
	Use other payment schemes	4	1%
	Develop disincentives	1	1%
	Use capitated payments	0	0%
	**Sum strategies**	**14**	
**Adapt and tailor to context**	Tailor strategies	6	2%
	Promote adaptability	0	0%
	Use data experts	0	0%
	Use data warehousing techniques	2	1%
	**Sum strategies**	**8**	
**Provide interactive assistance**	Facilitation	0	0%
	Provide local technical assistance	0	0%
	Centralize technical assistance	0	0%
	Provide clinical supervision	5	2%
	**Sum strategies**	**5**	
**Engage consumers**	Involve patients/consumers and family members	0	0%
	Intervene with patients/consumers to enhance uptake and adherence	0	0%
	Prepare patients/consumers to be active participants	0	0%
	Increase demand	0	0%
	Use mass media	2	1%
	**Sum strategies**	**2**	
**Added strategy by Perry et al.**	Assess and redesign workflow	21	7%
	Create online learning communities	0	0%
	Engage community resources	0	0%
**Strategies not found**	Accountability tool	22	7%
	FDA black box warning	1	1%
	Policy and regulations	5	2%
	Communication tool	9	3%
	International collaboration	1	1%

^a^Number exceeds total number of strategies since many of the studies used multiple strategies

Starting with the most common category, Table 3 presents the identified de-implementation strategies for each of the nine categories in ERIC [35], the added strategies by Perry et al. [36], and strategies not reflected in any of the previous strategies. A table with all identified strategies in each study is provided in Additional file 4.

#### Train and educate stakeholders

A majority of the identified de-implementation strategies were related to the category *train and educate stakeholders. Develop educational materials* (*n* = 106) was the most frequently used strategy in that category. This strategy was comprised of information tailored to various target audiences such as patients (e.g., [46, 47]) and practitioners (e.g., [48, 49]). *Make training dynamic* (*n* = 85) was the second most common strategy. This strategy included various types of staff trainings with active participation from the participants, including case studies, handouts, and a pre- and post-education knowledge test (e.g., [50, 51]). Another frequently used strategy included *distribute educational materials* (*n* = 59), which consisted of a more passive distribution of guidelines to practitioners (e.g., [52, 53]).

#### Use evaluative and iterative strategies

*Use of evaluative and iterative strategies* was the second most common category of ERIC strategies. Within this category, *audit and provide feedback* (*n* = 89) was the most frequently used de-implementation strategy. It included various types of targets for the feedback such as individuals (e.g., [49, 54] and teams (e.g., [55, 56]), as well as feedback targeted to high prescribers only (e.g., [57]) and combined with social comparisons (e.g., [58]) or benchmark data (e.g., [59]). Another common strategy within this group was *develop and organize a quality monitoring system* (*n*=86). This strategy consisted of monitoring systems electronically (e.g., [60, 61]) or via a pharmacist (e.g., [62, 63]) or peer (e.g., [64]). It also included feedback on the clinical outcomes of the reduced use of LVC (e.g., [65]).

#### Support clinicians

This category of ERIC strategies was the third most common. The majority of the identified de-implementation strategies in the category used the ERIC strategy *remind clinicians* (*n* = 92). This entailed digital (e.g., [66, 67]) or analog (e.g., [68, 69]) clinical decision support or other types of reminders such as stickers (e.g., [70]).

#### Develop stakeholder interrelationships

In this category of ERIC strategies, *use advisory boards and workgroups* (*n* = 28) was the most common de-implementation strategy. This category consisted of studies that had involved staff in planning the strategy (e.g., [71, 72]). Sixteen studies also used the strategy *identify and prepare champions* (e.g., [73, 74]), and 10 studies used *obtain formal commitments* (e.g., [75, 76])*.*

#### Change infrastructure

Within this category of ERIC strategies, the most frequently used strategy for de-implementation was *change physical structure and equipment* (*n* = 38). This encompassed changes in the ordering system for lab tests (e.g., [53, 77], changes in prescription process concerning medications (e.g., [78, 79]), facilitation of testing to only prescribe to patients with a certain test result (e.g., [80, 81]), facilitation of alternative practice (e.g., [82, 83]), and restricted availability of LVC practices (e.g., [84]).

#### Utilize financial strategies

In this category, *alter incentive/allowance structure* (*n* = 6) was the most common ERIC strategy. This strategy involved changing the level of reimbursement for LVC practices or the addition of criteria in the incentive system related to LVC use (e.g., [85]. The category *alter patient/consumer fees* (*n* = 3) included both increased patient costs for LVC (e.g., [86]) and reduced patient costs for diagnostic tests that may hinder non-indicated antibiotic prescriptions (e.g., [65]).

#### Adapt and tailor to context

In this category, *tailor strategies* (*n* = 6) was the most common ERIC strategy. The researchers used various methods to tailor the strategies to specific contexts, such as an educational strategy based on previously assessed knowledge among staff (e.g., [87]).

#### Provide interactive assistance

The only strategy that had been used for de-implementation in this category was *provide clinical supervision* (*n* = 5), which involved studies in which clinicians received supervision regarding when and how to use LVC practices and when other practices would be more beneficial for the patients. The supervision was either tailored to high prescribers (e.g., [88] or on demand for clinicians who requested it (e.g., [89]).

#### Engage consumers

The only strategy identified within this category was *use mass media* (*n* = 2). This strategy was represented by two studies in which an education campaign targeting the general population was conducted [90, 91].

### De-implementation strategies mapped to the strategies suggested by Perry et al. [36]

Twenty-one studies were based on strategies that matched the suggested added category: *assess and redesign workflow.* In the identified studies, the de-implementation strategies used included changes in coordinating patient follow-up within primary care (e.g., [62, 92]), the clinical pathway (e.g., [58, 93], and more general changes in work process (e.g., [87, 94]). The other two suggested strategies were not found in the literature.

### Strategies not found in ERIC

Four inductively coded strategies were not found in ERIC or the additional strategies suggested by Perry et al. [36]. These were *accountability tool* (*n* = 22), *black box warning* (*n* = 1), *policy and regulations* (*n* = 5), and *communication tool* (*n* = 9). *Accountability tools* included various tools that provided a gatekeeping function through which the clinicians were held accountable for their decision to use a low-value practice. They were requested to provide an argument as to why they were planning to use a low-value practice either by means of a written answer in the electronic health record or a verbal response to a colleague, specialist, or pharmacist (e.g., [95, 96]). *Black box warning*s were warning text on drug packages about the risk of the LVC drug so as to make both the clinician and patient aware of its risks (e.g., [97]). *Policy and regulations* had to do with directives instructing people to avoid using LVC (e.g., [47, 90]). *Communication tools* comprised a written script describing a process for communicating with patients about why they are not receiving a low-value practice, including processes for shared decision-making (e.g., [98, 99]).

## Discussion

This scoping review identified a total of 71 unique de-implementation strategies described in the literature. Of these, 62 strategies could be mapped onto ERIC compilation strategies, whereas four strategies could be mapped onto one of the added strategies [36]. Thus, 87% of de-implementation strategies reported across various fields overlapped with strategies used in implementation, while four identified strategies could not be mapped onto any existing implementation strategy. Two of these strategies (i.e., *policy and regulations* and *international collaboration*) are likely to be useful for both de-implementation and implementation, whereas *accountability tool, communication tool* and *black box warning* may be unique to de-implementation.

The most commonly used category of strategies was *train and educate stakeholders,* ranging from *distribute educational materials* to *make training dynamic*. These types of strategies are also prevalent strategies for implementation [100]. However, previous studies have suggested that education alone is insufficient for successful de-implementation [29] and implementation [101]. Colla et al. [29] found that educational strategies, combined with patient information and/or audit and feedback (i.e., multicomponent strategies), were more effective at reducing LVC. Regardless, as many as 24 of the studies in our review were based on the *train and educate stakeholders* category as the sole strategy. This suggests that de-implementation strategies may be chosen pragmatically, without much regard for research findings as to what is most effective.

Four de-implementation strategies were not possible to map onto ERIC or the additional strategies suggested by Perry et al. [36]. Three of these strategies may be unique to de-implementation and thus differ from the implementation process of introducing a new practice. Of these, the strategy *accountability tool* (*n* = 22) was most common and had the purpose of holding clinicians accountable when prescribing an LVC practice. It serves to disrupt the habitual use of a practice and forces clinicians to stop and reflect on whether they should prescribe the practice. This could be considered a more important strategy for de-implementation than implementation. The other strategies that may be unique for de-implementation were communication *tool* and *black box warning*. *Communication tools* consisted of a structured method for communicating with patients or next of kin about why a patient did not receive a practice, and *black box warning* consisted of a clear written warning on the packaging for certain medications. The other two strategies identified in this scoping review and not captured by ERIC or the additions by Perry et al. were policy and regulation and international collaboration. These two strategies might be relevant for both implementation and de-implementation, which could suggest that the ERIC compilation should be extended. Thus, as suggested in the study by Perry et al. [36], a potential limitation of the ERIC compilation is that all possible implementation strategies may not be covered. The original authors of ERIC similarly stated that the compilation should not be seen as the final word and welcomed comments and critique [31].

We found that only half (50%) of the 73 ERIC strategies had been used in the included studies. However, it is unclear whether the remaining strategies lack relevance or applicability for de-implementation or whether they have not been used for other reasons. Strategies from the category labeled *adapt and tailor to context* may be less applicable for de-implementation, where drift from protocols and guidelines may be the very reason for a practice becoming LVC. Examples include indication creep (when a practice is used for purposes for which it has not been proven efficient) and prevention creep (when a practice developed for symptomatic disease is used for asymptomatic individuals [102]. Other exemplary strategies that were rare in this review included the category *develop stakeholder relationships.* This might have considerable potential as a de-implementation strategy, since one determinant for the use of LVC is professionals’ expectations, attitudes, and behaviors [2]. Strategies such as informing local opinion leaders, identifying early adopters, or conducting local consensus discussions can influence professionals’ expectations, attitudes, and behaviors to support de-implementation.

Several of the de-implementation strategies that matched ERIC strategies involved more than one inductive code. For instance, the ERIC strategy *develop educational materials* involved development of information materials that comprised several codes in the inductive coding based on the material’s target: staff, providers, or patients. In fact, patient expectations have been found to be an important determinant for the use of LVC [2, 103], which suggests that strategies involving information for patients may be more important for de-implementation. The de-implementation strategies within the ERIC strategy *audit and provide feedback* entailed many types of audit and feedback. Some of these researchers used individual feedback, and others delivered group-level feedback. More innovative examples included quality-improvement contests and setting a goal for prescription and delivering rewards when reaching the goal. Finally, one frequent example was delivering feedback only to high prescribers, which seems to be a more specific de-implementation strategy because a small number of clinicians can have a large impact on the total amount of prescriptions [104]. Both of these examples indicate that some strategies are more multifaceted and heterogeneous than others, making it challenging to compare the effectiveness of strategies across studies. For future research on the de-implementation of LVC, various components of the strategies must be reported transparently and in detail, preferably using guidelines for specifying and reporting strategies [105].

Very few of the identified studies used any of the ERIC strategies that could be classified as pre-analysis approaches to assist in choosing the most suitable de-implementation strategies. The pre-analysis strategies found were *assess readiness and identify barriers and facilitators* (5% of the studies), *stage implementation scale-up* (1% of the studies), *conduct a local needs assessment* (3% of the studies), and *tailor strategies* (3% of the studies). This finding implies that the choice of de-implementation strategies is rarely tailored to the determinants of LVC use. In contrast, the importance of a comprehensive analysis of the current practice is considered crucial for successful implementation [101].

It is noteworthy that we could not find any studies within the behavioral health field (i.e., all studies were related to medicine). This could be due to the fact that it may be easier to determine that a medical practice is of low value because the efficacy or effectiveness of such practices can often be tested in trials that produce more unequivocal evidence. For instance, the problem of overprescribing antibiotics was defined in parallel with the development of the medication [106], whereas the side effects within psychotherapy research have only been investigated in recent years [107].

### Knowledge gaps and implications

The findings provide an overview of the most-used strategies within de-implementation. Most strategies could be found within ERIC, suggesting that the same type of strategies used for implementation purposes are also relevant for de-implementation. However, some new strategies were found that could be interpreted as more relevant to de-implementation than to implementation, including accountability and communication tool. The accountability tool provides a hurdle for routine use of LVC, and the communication tool helps the professional communicate their decision not to use LVC to patients or their families. Only half of the strategies described in ERIC was found in our review. This could be because some implementation strategies are irrelevant or under-utilized for de-implementation. Future studies could determine if some unused implementation strategies are also beneficial for de-implementation purposes.

Almost one-third of the studies in this review were focused on non-indicated antibiotics, implying that the most common strategies found in this review are a reflection of the most common strategies within the de-implementation of this type of LVC. One question that remains unanswered is whether different strategies could be beneficial for different types of LVC. For instance, patient centered care was suggested as an alternative to potentially inappropriate medications where individual strategies were described based on what caused anxiety to patients with dementia and how to best calm them without the unnecessary use of medications (e.g., [108]). This strategy is perhaps most suitable for patients within nursing homes or similar facilities and perhaps not for other patient populations. However, the lack of studies within behavioral health makes it difficult to assess the generalizability of the findings to this field.

### Methodological considerations

A considerable strength of this research is the number of studies included and the breadth of clinical fields covered. The study was also rigorous in its processes. We designed and performed the literature search in collaboration with the university library. Two reviewers screened all references independently. Two of the authors completed all coding and mapping and solved issues through discussions within the entire author group. However, there were also some limitations. The inconsistent terminology for LVC and de-implementation makes it plausible that studies may have been missed. Moreover, the review only covered literature written in English and published in peer-reviewed sources. We did not report the efficacy or effectiveness of various strategies because this was not the aim of the study. This allowed us to include a wider range of study designs including qualitative, process evaluations and cost-effectiveness studies and as an effect identify a wider range of strategies. Furthermore, the amount of information concerning the described strategies in the included studies varied, which may affect the trustworthiness of the inductive coding and the mapping onto the ERIC compilation. Data were charted individually, which may have influenced the information extracted from the articles. To ensure that relevant data were charted in a consistent way, an additional author piloted the data charting form, all individuals conducting the data charting were trained, and the charted data for a subset of articles were compared across individuals before starting the data charting. Finally, it is unknown if we would have received other results if we had coded the data based on another taxonomy. Future studies could investigate how de-implementation strategies differ depending on which taxonomy is used to code the strategies (e.g., [109]).

## Conclusions

The de-implementation strategies described in the literature overlap with implementation strategies to a large extent. However, only a limited number of the implementation strategies included in ERIC were represented as de-implementation strategies. This could imply that some strategies are underused or not applicable for de-implementation purposes. Nevertheless, the findings show that ERIC can be used to classify de-implementation strategies. We suggest some adaptions when using the ERIC compilation for de-implementation. The strategy *assess and redesign workflow* [36] and two new strategies, *accountability tool* and *communication tool* should complement the existing compilation when used for de-implementation purposes.

## Supplementary Information

**Additional file 1.** Preferred Reporting Items for Systematic reviews and Meta-Analyses extension for Scoping Reviews (PRISMA-ScR) Checklist.

**Additional file 2.** Documentation of search strategies.

**Additional file 3.** All inductive codes and their ERIC mapping.

**Additional file 4.** All identified strategies in each study.

## Data Availability

The datasets used will be available from the corresponding author on reasonable request.

## Abbreviations

LVC: Low-value care
ERIC: Expert Recommendation for Implementing Change
