# Supplementary material for: Strategies for de-implementation of low-value care—a scoping review
Source: Implement Sci. 2022 Oct 27;17:73. doi: 10.1186/s13012-022-01247-y (PMC9615304; doi:10.1186/s13012-022-01247-y)
Supplement: Supplementary file 3 — Additional file 3. All inductive codes and their ERIC mapping. [file 13012_2022_1247_MOESM3_ESM.docx]

| **ERIC** | **Inductive codes** |
| --- | --- |
| Access new founding |  |
| Alter incentive/allowance structures | Financial incentives, Cost reduction of diagnostic tests |
| Alter patient/consumer fees | Reduced cost for patients, increased costs for patients |
| Assess readiness and identify barriers and facilitators | Assess barriers and facilitators |
| Audit and provide feedback | Quality improvement contest, Goal setting and rewards, Assessment and feedback, Real time feedback, Audit and feedback, Targeted feedback (with peer comparison, benchmarking) |
| Build a coalition |  |
| Capture and share local knowledge |  |
| Centralize technical assistance |  |
| Change accreditation or membership requirements |  |
| Change liability laws |  |
| Change physical structure and equipment | Changes in test ordering system, Changes in prescription process, Facilitating of testing, Facilitating of alternative practice, Restriction in availability, adaption of equipment |
| Change record systems | Changes in electronic health record |
| Change service sites |  |
| Conduct cyclical small tests of change | PDSA |
| Conduct educational meetings | Lecture (staff), Patient education |
| Conduct educational outreach visits | Academic detailing, Case based staff training, Clinical round, Consultation, Consultation (ward rounds) |
| Conduct local consensus discussions | Consensus meeting |
| Conduct local needs assessment | Assess prevalence of LVC, pre-assessment of prescription practices |
| Conduct ongoing training |  |
| Create a learning collaborative | Network |
| Create new clinical teams | Multidisciplinary teams |
| Create or change credentialing and/or licensure standards |  |
| Develop a formal implementation blueprint |  |
| Develop academic partnerships |  |
| Develop an implementation glossary |  |
| Develop and implement tools for quality monitoring | Pharmacist documentation |
| Develop and organize quality monitoring systems | Electronic alert system, Medication review by pharmacist, Medication review, Effectiveness feedback, Peer feedback |
| Develop disincentives | Financial disincentives |
| Develop educational materials | Staff information, Provider information (by FDA), patient information |
| Develop resource sharing agreements |  |
| Distribute educational materials | Guideline, Local guideline |
| Facilitate relay of clinical data to providers | Diagnostic test |
| Facilitation |  |
| Fund and contract for the clinical innovation |  |
| Identify and prepare champions | Change champions |
| Identify early adopters |  |
| Increase demand |  |
| Inform local opinion leaders |  |
| Intervene with patients/consumers to enhance uptake and adherence |  |
| Involve executive boards |  |
| Involve patients/consumers and family members |  |
| Make billing easier |  |
| Make training dynamic | Staff training |
| Mandate change | Leadership engagement |
| Model and simulate change |  |
| Obtain and use patients/consumers and family feedback |  |
| Obtain formal commitment | Commitment tool |
| Organize clinician implementation team meetings | Peer support |
| Place innovation on fee for service lists/formularies |  |
| Prepare patients/consumers to be active participants |  |
| Promote adaptability |  |
| Promote network weaving |  |
| Provide clinical supervision | Tailored consultation, On demand consultation |
| Provide local technical assistance |  |
| Provide ongoing consultation |  |
| Purposively reexamine the implementation |  |
| Recruit, designate, and train for leadership |  |
| Remind clinicians | Clinical Decision Support (CDS), Reminders |
| Revise professional roles | Changes in staffing |
| Shadow other experts |  |
| Stage implementation scale up | Feasibility |
| Start a dissemination organization | National drug information center |
| Tailor strategies | Tailor strategies |
| Use advisory boards and workgroups | Involve staff |
| Use an implementation advisor |  |
| Use capitated payments |  |
| Use data experts |  |
| Use data warehousing techniques | Health Information Exchange |
| Use mass media | Public education |
| Use other payment schemes | Changes in reimbursement |
| Use train-the-trainer strategies | Train the trainer staff trainer |
| Visit other sites |  |
| Work with educational institutions |  |
| **Assess and redesign workflow** (1) | Coordination with primary care, Patient follow up, Clinical pathway, Changes in work process, |
|  | **ACCOUNTABILITY TOOL** |
|  | **FDA BLACK BOX WARNING** |
|  | **POLICY AND REGULATIONS** |
|  | **COMMUNICATION TOOL** |
|  | **INTERNATIONAL COLLABORATION** |

1. Perry CK, Damschroder LJ, Hemler JR, Woodson TT, Ono SS, Cohen DJ. Specifying and comparing implementation strategies across seven large implementation interventions: A practical application of theory. Implement Sci. 2019;14(1).
