# Supplementary material for: Strategies for de-implementation of low-value care—a scoping review
Source: Implement Sci. 2022 Oct 27;17:73. doi: 10.1186/s13012-022-01247-y (PMC9615304; doi:10.1186/s13012-022-01247-y)
Supplement: Supplementary file 4 — Additional file 4. All identified strategies in each study. [file 13012_2022_1247_MOESM4_ESM.docx]

| **Reference** | **Country** | **Setting** | **Design** | **Type of evaluation** | **Type of LVC** | **Low-value care practice** | **Guideline/**  **recommendation** | **Strategies used** |
| --- | --- | --- | --- | --- | --- | --- | --- | --- |
| (1) | US | Primary care | Quant pre-post | Efficacy/effectiveness | Imaging | Routine MRI | Diagnosis and treatment of low back pain: a joint clinical practice guideline from the American College of Physicians and the American Pain Society | Remind clinicians [ACCOUNTABILITY TOOL] |
| (2) | India | Multiple settings | Quant pre-post | Efficacy/effectiveness | Obstetrics | Shaving of pubic hair, routine enema, dorsal position for delivery, augmentation of labor, episiotomy for primis, fundal pressure, vaginal packing and routine suction of all new-borns | a) WHO Technical working group: care in normal birth: a practical guide  b) WHO reproductive health library c) WHO, UNPF, UNICEF, The world bank: pregnancy, childbirth, postpartum and new-born care: a guide for essential practice. 2nd ed. 2006 | Make training dynamic, audit and feedback |
| (3) | Pakistan | Hospital | Quant pre-post | Efficacy/effectiveness | Antibiotics | Antibiotic prophylaxis for gynecologic and obstetric procedures | The American College of Obstetricians and Gynecologists (ACOG) practice bulleting on antibiotic prophylaxis for gynecologic and obstetric procedures | Distribute educational materials, conduct educational meeting, develop and organize quality monitoring system, |
| (4) | US | Primary care | Quant RCT | Efficacy/effectiveness | Antibiotics | Inappropriate use of antibiotics (e.g., nonspecific upper respiratory tract infections, acute bronchitis, and influenza) | Principles of appropriate antibiotic use for treatment of nonspecific upper respiratory tract infections in adults. From Annals of Internal Medicine | Remind clinicians, [ACCOUNTABILITY TOOL] |
| (5) | Canada | Aged Care | Mixed – Case study | Feasibility | PIM elderly | Potentially inappropriate medication | Beer’s criteria | Remind clinicians |
| (6) | Canada | Aged Care | Quant pre-post | Efficacy/effectiveness | PIM elderly | Potentially inappropriate medications | Beers Criteria | Develop and organize quality monitoring systems |
| (7) | US | Aged Care | Quant pre-post | Efficacy/effectiveness | Antibiotics | inappropriate  antibiotic use in LTC residents with suspected UTI | Guidelines for the diagnosis and treatment of asymptomatic bacteriuria in adults. By Infectious Diseases  Society of America; American Society of Nephrology; American Geriatric  Society. Infectious Diseases Society of America. | Remind clinicians, make training dynamic, identify and prepare champions |
| (8) | UK | Aged Care | Mixed Case study | Efficacy/Effectiveness & Process evaluation | Anti-psychotics | Inappropriate prescribing of anti-psychotic medication | National Institute for Health and Clinical Excellence/Social Care Institute for Excellence. Guideline on supporting people with dementia and their carer’s in health and social care. 2007 London, British Psychological Society and Gaskell | Use train the trainer strategies |
| (9) | Norway | Hospital | Quant pre-post | Efficacy/Effectiveness | Antibiotics | Inappropriate use of antibiotics for CAP and AECOPD patients | The Norwegian Directorate of Health. National clinical guideline for use of antibiotics in hospital. 2013. | Remind clinicians, audit and provide feedback |
| (10) | Ireland | Primary care | Mixed RCT | Process evaluation | PIM elderly | Potentially inappropriate medications | Beer’s criteria, the STOPP criteria, the Improving Prescribing in the Elderly Tool | conduct educational outreach, remind clinicians, develop educational materials |
| (11) | Norway | Primary Care | Quant pre-post | Efficacy/Effectiveness | Corticosteroids COPD | Inappropriate use of inhaled corticosteroids  for COPD | Global strategy for the diagnosis, management, and prevention of chronic obstructive pulmonary disease: GOLD executive summary. | Provide clinical supervision |
| (12) | US | Hospital | Quant Pre-post | Efficacy/Effectiveness | Imaging | Pediatric head CT use | Identification of children at very low risk of clinically important brain injuries after head trauma: a prospective cohort study. From Lancet. 2009;374(9696):1160–1170 | Remind clinicians, audit and feedback, use advisory board and workgroups, [COMMUNICATION TOOL], assess and redesign workflow. |
| (13) | Portugal | Primary Care | Quant Quasi-experimental | Efficacy/Effectiveness | Lab tests | Inappropriate laboratory tests | The guide to clinical preventive services, 2014: recommendations of the U.S. Preventive Services Task Force. | Change physical structure and equipment |
| (14) | US | Hospital | Quant pre-post | Efficacy/Effectiveness | Lab tests | Inappropriate laboratory tests | Choosing wisely in adult hospital medicine | Change physical structure and equipment, conduct cyclical small tests of change. |
| (15) | US | Hospital | Quant pre-post | Efficacy/Effectiveness | Lab tests | Inappropriate C. difficile test | Multidrug-resistant organism & Clostridium difficile infection (MDRO/CDI) Module. Center for Disease Control and Prevention | Remind clinicians |
| (16) | US | Hospital | Quant pre-post | Efficacy/Effectiveness | Lab tests | Clostridium difficile infection (CDI) testing of non-diarrheal stool | Clostridium difficile infection in infants and children Committee on Infectious Diseases; American Academy of Pediatrics. | make training dynamic, remind clinicians, change physical structure and equipment |
| (17) | US | Hospital | Quant pre-post | Efficacy/Effectiveness | Lab tests | Inappropriate laboratory testing | Choosing Wisely | make training dynamic, remind clinicians |
| (18) | UK | Primary Care | Quant Quasi-experimental | Efficacy/Effectiveness | SMBG | Self-monitoring of blood glucose in people with type 2 diabetes who are not on insulin. | National Institute for Health and Care Excellence. Type 2 diabetes: the management of type 2 diabetes. London: NICE; 2014. | distribute educational materials, make training dynamic, develop educational materials, audit and provide feedback. |
| (19) | Australia | Primary Care | Mixed Cohort | Pilot investigation | Imaging | Low-back pain imaging | National Institute for Health and Clinical Excellence (NICE). Low back pain: early management of persistent non-specific low back pain. | make training dynamic, audit and provide feedback, remind clinicians. |
| (20) | Spain | Aged Care | Quant Prospective randomized trial | Efficacy/Effectiveness | PIM elderly | Potentially inappropriate medications | STOPP (Screening Tool of Older Persons Prescriptions) and START (Screening Tool to Alert Doctors to Right Treatment) | make training dynamic, provide clinical supervision |
| (21) | Italy | Other | Quant cross-sectional | Efficacy/Effectiveness | PIM elderly | Potentially inappropriate medications | Beers Criteria | make training dynamic, remind clinicians |
| (22) | US | Hospital | Quant pre-post | Efficacy/Effectiveness | Infants/proton pump inhibitors | Use of PPIs/H2RAs in both term and  preterm infants | Pediatric gastroesophageal  reflux clinical practice guidelines:  joint recommendations of the North American Society for Pediatric  Gastroenterology, Hepatology,  and Nutrition (NASPGHAN) and the  European Society for Pediatric  Gastroenterology, Hepatology, and  Nutrition (ESPGHAN). | distribute educational materials, develop educational materials, conduct educational meetings, assess and redesign workflow, audit and provide feedback, revise professional roles, remind clinicians. |
| (23) | US | Multiple settings | Quant Quasi-experimental | Evaluate Impact/association | Lab tests | Routine preoperative lab testing | Practice advisory for paranaesthesia evaluation: an updated report by the American Society of Anaesthesiologists Task Force on Paranaesthesia Evaluation.  Guidelines on perioperative cardiovascular evaluation and care for noncardiac surgery: a report of the American College of Cardiology/American Heart Association Task Force on Practice Guidelines | Distribute educational materials |
| (24) | US | Primary Care | Mixed Case study | Process evaluation | Antibiotics | Inappropriate use of antibiotics for acute respiratory tract infections | Chronic cough due to acute bronchitis: ACCP evidence-based clinical practice guidelines. | remind clinicians, develop educational materials, make training dynamic, identify and prepare champions, audit and provide feedback. |
| (25) | US | Hospital | Quant Longitudinal study | Efficacy/Effectiveness | Imaging | Use of diagnostic neuroimaging in the evaluation of headache | Practice parameter: evidence-based guidelines for migraine headache (an evidence-based review): report of the Quality Standards Subcommittee of the American Academy of Neurology. | Use data warehousing techniques |
| (26) | US | Hospital | Quant Pre-post | Efficacy/Effectiveness | echocardiograph | Inappropriate Transthoracic echocardiography | ACCF/ASE/AHA/ASNC/HFSA/HRS/SCAI/SCCM/SCCT/SCMR 2011 appropriate use criteria for echocardiography: a report of the American College of Cardiology Foundation Appropriate Use Criteria Task Force, | make training dynamic, remind clinicians, audit and provide feedback. |
| (27) | US | Hospital | Quant pre-post | Efficacy/ Effectiveness | PIM Elderly | Potentially inappropriate medication | STOPP (Screening Tool of Older  Person’s Prescriptions) and START (Screening Tool to Alert doctors to  Right Treatment). | create new clinical teams, develop and organize quality monitoring systems |
| (28) | Switzerland | Hospital | Quant pre post | Efficacy/Effectiveness | Antibiotics | Inappropriate use of antibiotics for asymptomatic bacteriuria | Diagnosis, prevention, and treatment of catheter-associated  urinary tract infection in adults: 2009 international clinical practice  guidelines from the Infectious Diseases Society of America. | Distribute educational materials, develop educational materials, make training dynamic, conduct educational meetings, conduct educational outreach, [ACCOUNTABILITY TOOL], remind clinicians. |
| (29) | Sweden | Hospital | Quant RCT | Efficacy/Effectiveness | PIM Elderly | Potentially inappropriate medications | STOPP (Screening Tool of Older Person’s Prescriptions) and START (Screening Tool to Alert doctors to Right Treatment | Develop and organize quality monitoring systems, conduct educational meeting, assess and redesign workflow. |
| (30) | US | Primary care | Quant RCT | Efficacy/Effectiveness | Antibiotics | Inappropriate use  of antibiotics in primary care settings | Principles of appropriate  antibiotic use for treatment of uncomplicated acute bronchitis American Academy of Family Physicians; American College of Physicians–American Society of Internal Medicine; Centers  for Disease Control; Infectious Diseases Society of America.  Principles of appropriate antibiotic use for treatment of nonspecific  upper respiratory tract infections in adults. Academy of Family Physicians; Infectious Diseases Society of America; Centers for Disease Control; American College of Physicians–American Society of Internal Medicine. | Remind clinicians, make training dynamic, identify and prepare champions, audit and provide feedback, develop educational materials. |
| (31) | US | Hospital | Quant pre-post | Efficacy/Effectiveness | Acid suppression medication | Inappropriate prescription of Acid suppression medications (AST) | ASHP therapeutic guidelines on stress ulcer pro-phylaxis. American Society of Health System Pharmacists | conduct educational meetings, develop and organize quality monitoring systems |
| (32) | US | Primary care | Quant Quasi-experimental | Efficacy/Effectiveness | Antibiotics | Inappropriate use  of antibiotics in primary care settings | Antibiotic prescribing and use. Centers for Disease Control and Prevention. | Assess and redesign workflow, develop educational materials |
| (33) | US | Hospital | Quant longitudinal | Association | Antibiotics | Treatment with bronchodilators, corticosteroids, antibiotics, and utilization of chest radiographs "in the management of children with bronchiolitis" | Diagnosis and Management of Bronchiolitis. American Academy of Pediatrics Sub-committee | Distribute educational materials |
| (34) | Italy | Primary care | Quant quasi-experimental | Efficacy/effectiveness | PIM Elderly | Potentially inappropriate medication | Beer’s criteria | Distribute educational materials, develop and organize quality monitoring systems, make training dynamic, audit and provide feedback, use advisory boards and workgroups. |
| (35) | US | Primary Care | Quant longitudinal | Efficacy/Effectiveness | Antibiotics | Inappropriate use of antibiotics for ARI in primary care practices | Management of community-acquired pneumonia in adults. Infectious Diseases Society of America/American Thoracic Society | Remind clinicians, conduct educational outreach, audit and provide feedback. |
| (36) | US | Primary Care | Quant Quasi-Experimental | Efficacy/Effectiveness | Antibiotics | Inappropriate use of antibiotics for ARI in primary care practices | Prescribing of antibiotics for Self-Limiting Respiratory Tract Infections in Adults and Children in Primary Care. National Institute for Health and Clinical Excellence.  Antibiotic prescribing and Use. Centers for Disease Control and Prevention. | remind clinicians, audit and provide feedback, conduct educational outreach, make training dynamic |
| (37) | Canada | Aged Care | Quant longitudinal | Efficacy/Effectiveness | Antipsychotics | Inappropriate prescribing of antipsychotic medication | Recommendations for the management of behavioral and psychosocial symptoms of dementia. From the Canadian Journal of Neurological Sciences.  Guidelines for the management of agitation in dementia. From the International Journal of Geriatric Psychiatry.  Pharmacological treatment of neuro-psychiatric symptoms of dementia: from the Journal of the American Medical Association. | make training dynamic, develop and organize quality monitoring systems |
| (38) | Iran | Hospital | Quant pre-post | Efficacy/Effectiveness | Acid suppression medication | Inappropriate AST prescribing | ASHP therapeutic guidelines on stress ulcer prophylaxis. American Society of Health-System Pharmacists. | conduct educational meeting, distribute educational materials, conduct educational outreach |
| (39) | Norway | Primary Care | Quant RCT | Efficacy/Effectiveness | PIM Elderly | Potentially inappropriate medication | Beers Criteria  Indicators for evaluation of the quality of drug therapy in the elderly. Swedish National Board of Health and Welfare [Socialstyrelsen]. | conduct educational outreach, audit and provide feedback, distribute educational materials, make training dynamic |
| (40) | US | Multiple setting | Mixed Pre-post | Efficacy/Effectiveness | Imaging | Inappropriate radionuclide imaging (RNI) studies | Appropriate use criteria for Imaging. The American College of Cardiology. | create learning collaborative, make training dynamic, audit and provide feedback |
| (41) | US | Hospital | Quant pre-post | Efficacy/Effectiveness | Imaging | Unindicated Dual-Phase Head CT Examinations | Appropriateness Criteria. From the American College of Radiology | distribute educational materials, develop educational materials, provide clinical supervision, audit and provide feedback, [ACCOUNTABILITY TOOL] |
| (42) | US | Multiple settings | Quant Cohort | Efficacy/Effectiveness | Echocardiography | Inappropriate stress echocardiography | Appropriateness criteria for trans-thoracic and transesophageal echocardiography: a report of the American College of Cardiology Foundation Quality Strategic Directions Committee Appropriateness Criteria Working Group.  Appropriateness criteria for stress echo: a report of the American College of Cardiology Foundation Appropriateness Criteria Task Force  Appropriate use criteria for echocardiography: a report of the American College of Cardiology Foundation Appropriate Use Criteria Task Force. | conduct educational meetings, develop educational materials |
| (43) | Cameroon | Multiple settings | Mixed Case study | Process evaluation | Anti-malarials | Over-diagnosis, over-prescription of anti-malarials and under-prescription of ACT | Guidelines for the Treatment of Malaria. WHO | use train the trainer techniques |
| (44) | US | Hospital | Quant interrupted time series analysis | Efficacy/Effectiveness | Antibiotics | Inappropriate use of viral testing, chest radiograph (CXR), and albuterol or antibiotics | Diagnosis and Management of Bronchiolitis. American Academy of Pediatrics Sub-committee | Distribute educational materials, remind clinicians, make training dynamic, develop educational materials, audit and provide feedback, provide clinical supervision. |
| (45) | US | Hospital | Quant interrupted time series analysis | Efficacy/Effectiveness | Echocardiography | Inappropriate Transthoracic echocardiography (TTE) | Appropriateness criteria for transthoracic and transesophageal echo-cardiography: A report of the American College of Cardiology Foundation Quality Strategic Directions Committee Appropriateness Criteria Working Group.  Appropriate Use Criteria for Echocardiography. A Report of the American College of Cardiology Foundation Appropriate Use Criteria Task Force. | conduct educational meetings, distribute educational materials, audit and provide feedback |
| (46) | Thailand | Hospital | Quant pre-post | Efficacy/Effectiveness | Antibiotics | Inappropriate use of antibiotics | Guideline for the management of acute diarrhea in adults. From the Journal of Gastroenterol Hepatology  Practice guidelines for the management of infectious diarrhea. From the Journal of Clinical  Infectious Diseases  Clinical practice. Bacterial diarrhea. From the New English Journal of Medicine.  Clinical practice. Acute infectious diarrhea. From the New England Journal of Medicine. | Distribute educational materials, develop educational materials, make training dynamic, develop and organize quality monitoring systems, alter patient/consumer fees, audit and provide feedback. |
| (47) | Australia | Hospital | Quant pre-post | Efficacy/Effectiveness | Antibiotics | Inappropriate use of antibiotics | Therapeutic guidelines: antibiotic. From the Antibiotic Expert Group. | audit and provide feedback, remind clinicians |
| (48) | Norway | Hospital | Quant quasi-experimental | Efficacy/Effectiveness | Antibiotics | Inappropriate use of fluoroquinolone for cystitis | Bruk av antibiotika i sykehus Smittevernloven — håndbok. (Infection control handbook. Inpatient use of antibiotics).  Nasjonale faglige retningslinjer for antibiotikabruk i primærhelsetjenesten (National guidelines for antibiotic prescribing in primary care). | Remind clinicians, change physical structure and equipment |
| (49) | UK | Aged Care | Quant prospective randomized controlled trial | Efficacy/Effectiveness | Antibiotics | Inappropriate use of antibiotics | Loeb Criteria | remind clinicians |
| (50) | US | Hospital | Quant pre-post | Efficacy/Effectiveness | Antibiotics | Inappropriate use of antibiotics for asymptomatic bacteriuria | Guidelines for the diagnosis and treatment of asymptomatic bacteriuria in adults. From Journal of Clinical Infectious Diseases. | Develop educational materials, remind clinicians, conduct educational outreach, develop and organize quality monitoring system. |
| (51) | Italy | Primary Care | Quant Quasi experimental | Efficacy/Effectiveness | PIM Elderly | Potentially inappropriate medications | Beer’s criteria | distribute educational materials, make training dynamic, conduct educational outreach. |
| (52) | US | Primary Care | Quant Longitudinal | Efficacy/Effectiveness | antibiotics | Inappropriate use of antibiotics for outpatient cases of acute bronchitis and URI. | Principles of judicious use of antimicrobial agents for pediatric upper respiratory tract infections. From Pediatrics (Journal).  Principles of appropriate antibiotic use for treatment of acute bronchitis in adults. From Annals of Internal Medicine (Journal). | remind clinicians |
| (53) | US | Primary Care | Quant RCT | Efficacy/Effectiveness | Antibiotics | Inappropriate use of antibiotics | Principles of appropriate antibiotic use for treatment of acute respiratory tract infections in adults. From Annals of Internal Medicine (Journal).  Chronic cough due to acute bronchitis: ACCP evidence-based clinical practice guidelines. | obtain formal commitment |
| (54) | UK | Primary Care | Quant Pre-post | Efficacy/Effectiveness | antiplatelet therapy | Inappropriate antiplatelet therapy | The management of atrial fibrillation. National Institute for Health and Care Excellence.  the ESC Guidelines for the management of atrial fibrillation. | Make training dynamic, remind clinicians, audit and provide feedback, use advisory boards and workgroups. |
| (55) | US | Primary Care | Qual Case Study | Process evaluation | Antibiotics | Inappropriate use of antibiotics | The management of community-acquired pneumonia in infants and children older than 3 months of age: clinical practice guidelines by the Pediatric Infectious Diseases Society and the Infectious Diseases Society of America. | Make training dynamic, audit and provide feedback |
| (56) | US | Aged Care | Quant Quasi Experimental | Efficacy/Effectiveness | Antibiotics | Inappropriate use of antibiotics Nursing Homes | Loeb Criteria | Make training dynamic, audit and feedback, develop educational materials, remind clinicians |
| (57) | US | Hospital | Quant Pre-post | Efficacy/Effectiveness | Stress ulcer prophylaxis | Inappropriate stress ulcer prophylaxis | ASHP Gastrointestinal Stress Ulcer Prophylaxis guidelines. | make training dynamic, remind clinicians |
| (58) | Thailand | Hospital | Quant Pre-post | Efficacy/Effectiveness | Antibiotics | Inappropriate use of antibiotics | Use of antibiotics: a brief exposition of the problem and some tentative solutions from Annals of Internal Medicine (Journal). | Make training dynamic, develop and organize quality monitoring system |
| (59) | US | Aged Care | Quant Cross sectional /longitudinal study | Efficacy/Effectiveness | Antipsychotics | Inappropriate prescribing of antipsychotic medications | AGS Guide to the Management of Psychotic Disorders and Neuropsychiatric Symptoms of Dementia in Older Adults  Interpretive Guidelines for Long-Term Facilities by Centers for Medicare and Medicaid Services | audit and provide feedback |
| (60) | US | Hospital | Quant pre-post | Efficacy/Effectiveness | Stress ulcer prophylaxis | Inappropriate stress ulcer prophylaxis | Clinical practice guidelines for Clostridium difficile infection in adults: by the Society for Healthcare Epidemiology of America (SHEA) and the Infectious Diseases Society of America (IDSA) | develop and organize quality monitoring system |
| (61) | UK | Hospital | Quant pre-post | Efficacy/Effectiveness | Antibiotics | Inappropriate use of antibiotics in hospital | Scottish Intercollegiate Guidelines Network, Antibiotic Prophylaxis in Surgery. | Audit and provide feedback |
| (62) | US | Multiple settings | Quant pre-post | Efficacy/Effectiveness | screening | Over-screening for cervical cancer in young women | ACOG practice bulletin: Cervical cytology screening. | Make training dynamic |
| (63) | US | Multiple settings | Quant pre-post | Efficacy/Effectiveness | Radiotherapy | the use of extended fractionation schemes (>10 fractions) | Palliative radiotherapy for bone metastases: An ASTRO evidence-based guideline. | Assess and redesign workflow, remind clinicians, use advisory boards and workgroups, develop and organize quality monitoring systems. |
| (64) | Australia | Hospital | Quant pre-post | Process evaluation | Catheters | Indwelling urinary catheters (IUC) | Guideline for prevention of catheter-associated urinary tract infections by the Healthcare Infection Control Practices Advisory Committee. | Distribute educational materials, identify and prepare champions, use advisory boards and workgroups, make training dynamic, remind clinicians. |
| (65) | Serbia | Aged Care | Quant pre-post | Efficacy/Effectiveness | PIM elderly | Potentially inappropriate medications | Beers Criteria  STOPP (Screening Tool of Older Person’s Prescriptions) and START (Screening Tool to Alert doctors to Right Treatment). | Conduct educational meeting, develop educational materials. |
| (66) | US | Hospital | Quant quasi-experimental | Efficacy/Effectiveness | Imaging | Inappropriate head CTs in patients with MTBI | ACEP's campaign to reduce unnecessary testing in the emergency department. | Remind clinicians |
| (67) | Canada | Hospital | Quant Quasi-experimental | Efficacy/Effectiveness | Antibiotics | Inappropriate use of antibiotics for asymptomatic bacteriuria | Guidelines for the diagnosis and treatment of asymptomatic bacteriuria in adults. By the Infectious Diseases Society of America | Make training dynamic |
| (68) | India | Hospital | Quant pre-post | Efficacy/Effectiveness | PIM Elderly | Potentially inappropriate medication | Beer Criteria | Audit and provide feedback |
| (69) | US | Primary care | Quant pre-post | Efficacy/Effectiveness | Antibiotics, Imaging | Inappropriate use of antibiotics for sinusitis and papanicolaou tests, dual-energy X-ray absorptiometry (DEXA), and electro cardio-graphic (ECG) screening, testing. | Choosing wisely | Make training dynamic |
| (70) | US | Hospital | Quant pre-post | Efficacy/Effectiveness | PIM elderly | Potentially inappropriate medication (diphenhydramine, metoclopramide) and inappropriate use of antipsychotics | Beers Criteria | Remind clinicians |
| (71) | Kenya | Primary Care | Quant RCT | Efficacy/Effectiveness | Antimalarials | Over prescription of antimalarials | Guidelines for the treatment of malaria. World Health Organization (WHO). | Alter incentive/allowance structures |
| (72) | Spain | Aged Care | Quant pre-post | Efficacy/Effectiveness | PIM elderly | Potentially inappropriate medication | Beers Criteria | develop educational materials, make training dynamic, develop and organize quality monitoring system |
| (73) | Kenya | Multiple settings | Quant pre-post | Efficacy/Effectiveness | Antimalarials | Over prescription of antimalarials | Guidelines for the treatment of malaria. World Health Organization (WHO). | make training dynamic, change physical structure and equipment |
| (74) | Italy | Primary Care | Quant pre-post | Efficacy/Effectiveness | NSAIDs and COXIB | Inappropriate use of NSAIDs and COXIB by GPs in patients with HF, CAD, CVD and PAD after hospital discharge. | A 2012 amendment of a previous Italian legislative decree (first published in 2009), the Note 66 (17), released by the Italian drug regulatory authority Agenzia Italiana del Farmaco (AIFA) | Remind clinicians |
| (75) | US | Hospital | Mixed Between subject experiment | Efficacy/Effectiveness | Imaging | Inappropriate diagnostic imaging orders | ACR appropriateness criteria. | remind clinicians, use advisory boards and workgroups |
| (76) | US | Hospital | Quant pre-post | Efficacy/Effectiveness | Pulse oximetry | Overuse of CPOx for children on room air | Clinical practice guideline: the diagnosis, management, and prevention of bronchiolitis. From Pediatrics (Journal). | Develop educational material, distribute educational materials, remind clinicians, audit and provide feedback, use advisory boards and workgroups, change physical structure or equipment, assess and redesign workflow, tailor strategies |
| (77) | US | Primary Care | Quant prospective interrupted time series | Efficacy/Effectiveness | Screening | PSA-based screening for prostate cancer in men aged 75 years and older. | NCCN Clinical Practice Guidelines in Oncology: Prostate Cancer Early Detection  Early detection of prostate cancer guideline. By the American Urological Association. | Remind clinicians, use advisory boards and workgroups |
| (78) | Canada | Hospital | Quant Pre-post | Efficacy/Effectiveness | Antibiotics | Antibiotic prophylaxis | Guideline for prevention of surgical  site infection, by the Centers for Disease Control and Prevention (CDC)  Antimicrobial prophylaxis for surgery: an advisory statement from the National Surgical Infection Prevention Project.  Quality standard for antimicrobial  prophylaxis in surgical procedures. Infectious Diseases Society of America. | develop educational materials, make training dynamic, audit and provide feedback |
| (79) | US | Hospital | Quant Pre-post | Efficacy/Effectiveness | PIM Elderly | Potentially inappropriate medications | Beer’s criteria. | make training dynamic, remind clinicians, audit and provide feedback, conduct educational outreach, use advisory boards and workgroups. |
| (80) | Spain | Primary Care | Quant Quasi experimental | Efficacy/Effectiveness | Antibiotics | Inappropriate use of Antibiotics in Acute exacerbation of chronic obstructive pulmonary disease | CTS Guideline - Highlights for Primary Care - Recommendations for Management of COPD.  Global Strategy for the Diagnosis, Management and Prevention of COPD, Global Initiative for Chronic Obstructive Lung Disease (GOLD)  the Anthonisen Criteria (AC): Antibiotic therapy in exacerbations of chronic obstructive pulmonary disease. From Annals of Internal Medicine (Journal). | Audit and provide feedback, make training dynamic, develop educational materials, facilitate relay of clinical data. |
| (81) | US | Hospital | Quant pre-post | Efficacy/Effectiveness | Antibiotics | Inappropriate use of antibiotics for asymptomatic bacteriuria in patients with urinary  catheters | Infectious Diseases Society of America guidelines for the  diagnosis and treatment of asymptomatic bacteriuria in adults.  Infectious Diseases Society of America. Diagnosis, prevention, and treatment of catheter-associated urinary tract infection in adults. | Remind clinicians, make training dynamic. |
| (82) | US | Hospital | Quant pre-post | Efficacy/Effectiveness | Echocardiography | Inappropriate use of echocardiography for mild, asymptomatic native valve disease in adult patients with no change in signs or symptoms’ | Appropriate Use Criteria for Echocardiography: A Report of the American College of Cardiology Foundation Appropriate Use Criteria Task Force.  National Institute for Health and Care Excellence. Chronic heart failure: management of chronic heart failure in adults in primary and secondary care. | remind clinicians, use advisory boards and workgroups |
| (83) | UK | Aged Care | Mixed Case study | Process evaluation | Antipsychotics | Inappropriate prescribing of antipsychotics in care homes | Optimising treatment and care for behavioural and psychological symptoms of dementia. Alzheimer’s Society. | Make training dynamic |
| (84) | Canada | Hospital | Quant pre-post | Efficacy/Effectiveness | PIM Elderly | Potentially Inappropriate Medication in hospitalized elderly adults | Beers Criteria | Develop educational materials, conduct educational meetings, develop and organize quality monitoring systems |
| (85) | US | Hospital | Quant pre-post | Efficacy/Effectiveness | Imaging | Inappropriate imaging | American College of Emergency Physicians. Clinical policy: critical issues in the evaluation and management of emergency department patients with suspected appendicitis. | Assess and redesign workflow, conduct educational meeting, develop educational material, remind clinicians, use advisory boards and workgroups. |
| (86) | US | Hospital | Quant pre-post | Efficacy/Effectiveness | Antibiotics | Inappropriate use of antibiotics | Infectious Diseases Society of America (IDSA) guidelines and the Global Initiative for Chronic Obstructive Lung Disease (GOLD) guidelines for appropriate Type of Infection, drug selection and duration. | Develop and organize quality monitoring system. |
| (87) | US | Multiple settings | Quant Cohort | Efficacy/Effectiveness | Radiotherapy | Inappropriate use of androgen-deprivation therapy for patients with early prostate cancer | NCCN practice guidelines for prostate cancer.  NCCN Practice Guidelines in Oncology | Use other payment schemes |
| (88) | Canada | Primary Care | Quant pre-post | Efficacy/Effectiveness | Lab tests | Inappropriate use of selected clinical chemistry tests | Evidence of the clinical usefulness of each of the tests in this study was derived from literature searches in addition to various national guidelines that were based on extensive reviews. | Conduct educational meetings, audit and feedback, develop educational material, change physical structure and equipment, distribute educational materials. |
| (89) | US | Hospital | Quant pre-post | Efficacy/Effectiveness | Lab tests | Inappropriate urine cultures | CAUTIs. US Centers for Disease Control and Prevention. | facilitate relay of clinical data |
| (90) | Italy | Hospital | Quant RCT | Efficacy/Effectiveness | PIM Elderly | Potentially inappropriate medication for hospitalized elderly adults | Beers Criteria | make training dynamic, remind clinicians |
| (91) | US | Multiple settings | Quant Cross sectional | Efficacy/Effectiveness | Imaging | Inappropriate advanced outpatient imaging studies (CT, MRI, PET, nuclear medicine) | Practice Guidelines of the American College of Physicians, Practice Guidelines of the American Academy of Neurology, and American College of Radiology Appropriateness Criteria | Distribute educational materials, make training dynamic, [ACCOUNTABILITY TOOL]. |
| (92) | US | Hospital | Quant pre-post | Efficacy/Effectiveness | Cardiac testing | Inappropriate Use of Cardiac Stress Tests with Imaging | Appropriate use criteria for cardiac radionuclide imaging: a report of the American College of Cardiology  Appropriate use criteria Task Force.  Appropriate use criteria for echocardiography: a report of the American College of Cardiology Foundation appropriate use criteria Task Force. | change physical structure and equipment |
| (93) | US | Primary Care | Quant Cross sectional | Process evaluation | Antibiotics | Inappropriate use of antibiotics | Clinical practice guideline for the diagnosis and management of group A streptococcal pharyngitis: By the Infectious Diseases Society of America.  Diagnosis and management of cough ACCP evidence-based clinical practice guidelines.  Infectious Diseases Society of America/American Thoracic Society consensus guidelines on the management of community-acquired pneumonia in adults. | remind clinicians, [ACCOUNTABILITY TOOL], audit and provide feedback, make training dynamic |
| (94) | Colombia | Hospital | Quant Pre-post | Efficacy/Effectiveness | Lab tests, Imaging, other medications | Inappropriate use of diagnostic tests, inappropriate use of chest radiography and routine use of inhaled bronchodilators | Clinical practice guideline on acute bronchiolitis: clinical practice guidelines in the Spanish national healthcare system. | Make training dynamic |
| (95) | Multiple countries | Primary Care | Quant Cross sectional | Efficacy/Effectiveness | Antibiotics | Inappropriate use of antibiotics | WHO Global Strategy for containment of antibiotic resistance.  WHO Global Action plan on antibiotic resistance.  WHO Global Action plan on antibiotic resistance. | [POLICY AND REGULATIONS], distribute educational materials, conduct educational meeting, use mass media, alter incentive/allowance structures, alter patient/consumer fees, start a dissemination organization |
| (96) | UK | Primary Care | Quant Pre-post | Efficacy/Effectiveness | Domperidone | Over prescription of domperidone | New advice for domperidone by the Medicines and Healthcare products Regulatory Agency | Audit and provide feedback |
| (97) | US | Primary Care | Quant Pre-post | Efficacy/Effectiveness | Imaging | Osteoporosis screening with dual-energy X-ray absorptiometry (DXA) scans. | Osteoporosis screening by the US Preventive Services Task Force  Choosing Wisely | Distribute educational materials |
| (98) | Netherlands | Hospital | Quant pre-post | Efficacy/Effectiveness | Lab tests | antinuclear antibody (ANA) test overuse | Choosing Wisely | Make training dynamic |
| (99) | US | Hospital | Quant pre-post | Efficacy/Effectiveness | Antibiotics | Inappropriate use of antibiotics | ACCP evidence-based clinical practice guidelines.  Principles of appropriate antibiotic use for treatment of uncomplicated acute bronchitis: From the Annals of Internal Medicine (Journal).  Principles of appropriate antibiotic use for treatment of acute bronchitis in adults. From the Annals of Internal Medicine (Journal). | Make training dynamic |
| (100) | US | Primary Care | Quant RCT | Efficacy/Effectiveness | Imaging | Inappropriate use of spinal magnetic resonance imaging (MRI) for sub-acute back pain, dual-energy x-ray absorptiometry (DXA) screening for postmenopausal women at low risk for osteoporosis, and neuroimaging for recent-onset uncomplicated headache | Adult acute and subacute low back pain. Institute for Clinical Systems Improvement.  Diagnosis and treatment of osteoporosis. Institute for Clinical Systems Improvement. | Make training dynamic |
| (101) | England | Hospital | Mixed RCT | Efficacy/Effectiveness Process evaluation | Antibiotics | Inappropriate use of antibiotics | The Trust’s antimicrobial guidelines. | Make training dynamic |
| (102) | Australia | Primary Care | Quant pre-post | Efficacy/Effectiveness | Lab tests | Inappropriate lab test | Diagnostic approach to fatigue in primary care. Melbourne, Australia: Therapeutic Guidelines Limited.  Guidelines for preventive activities in general practice. By the Royal Australian College of General Practitioners.  Evidence-based management of acute musculoskeletal pain. By the Australian Acute Musculoskeletal Pain Guidelines Group. | Make training dynamic |
| (103) | England | Hospital | Quant Cross sectional | Process evaluation | Antibiotics | Inappropriate use of antibiotics | Respiratory tract infections (self-limiting): prescribing antibiotics. National Institute for Health and Care Excellence. | Distribute educational materials |
| (104) | US | Primary Care | Quant RCT | Efficacy/Effectiveness | Antibiotics | Inappropriate use of antibiotics | Principles of appropriate antibiotic use for treatment of uncomplicated acute bronchitis: From the Annals of Internal Medicine (Journal).  Principles of appropriate antibiotic use for acute rhinosinusitis in adults: From the Annals of Internal Medicine (Journal).  Principles of appropriate antibiotic use for acute pharyngitis in adults: From the Annals of Internal Medicine (Journal).  Principles of appropriate antibiotic use for treatment of nonspecific upper respiratory tract infections in adults: From the Annals of Internal Medicine (Journal). | Develop educational materials remind clinicians, [ACCOUNTABILITY TOOL], audit and provide feedback, change physical structure and equipment. |
| (105) | Cyprus | Hospital | Quant Pre-post | Efficacy/Effectiveness | Lab tests | Inappropriate lab tests | Guidelines for laboratory ordering in Emergency Department. By the Committee for clinical guidelines for laboratory tests. | distribute educational materials, change physical structure and equipment, develop disincentives |
| (106) | Italy | Hospital | Quant Pre-post | Efficacy/Effectiveness | Antibiotics | Inappropriate use of antibiotics | WSES guidelines for management of intra-abdominal infections. | Distribute educational materials, make training dynamic, develop educational materials, develop and organize quality monitoring systems, [ACCOUNTABILITY TOOL], audit and provide feedback, |
| (107) | Korea | Multiple settings | Quant Pre-post | Efficacy/Effectiveness | Pregnancy | Pregnancy-contraindicated drug use | Public Announcement of Pregnancy-contra indicated Drugs. By the Korea Ministry of Food and Drug Safety. | [ACCOUNTABILITY TOOL] |
| (108) | US | Multiple settings | Quant Pre-post | Efficacy/Effectiveness | Imaging | Inappropriate use of knee MRI studies for patients with moderate to severe knee osteoarthritis | ACR Appropriateness Criteria non traumatic knee pain.  Treatment of osteoarthritis of the knee: evidence-based guideline. By the American Academy of Orthopaedic Surgeons.  College of Rheumatology 2012 recommendations for the use of non-pharmacologic and pharmacologic therapies in osteoarthritis of the hand, hip, and knee. | Distribute educational material |
| (109) | US | Hospital | Qual Case study | Process evaluation | lab tests, Imaging, echocardiograph, ANA, colonoscopy | Inappropriate measure of creatinine on patients receiving scheduled dialysis, DXA scan on a women less than 65 without clinical risk factors for early osteoporosis, routine daily chest X-rays on intubated patients, repeat a positive hepatitis A or C antibody, PET or bone scan imaging on asymptomatic women with stage I or stage II breast cancer, repeat a positive ANA, an echocardiogram in the setting of heart failure, elective screening colonoscopy on patients older than 75 years | High-value, cost-conscious health care: concepts for clinicians to evaluate the benefits, harms, and costs of medical interventions. Clinical Guidelines Committee of the American College of Physicians  Choosing Wisely | Use advisory boards and workgroups, Identify and prepare champions, conduct educational meeting, change record system, assess and redesign workflow, audit and provide feedback, conduct local needs assessment |
| (110) | UK | Multiple settings | Quant Pre-post | Efficacy/Effectiveness | Lab tests | Inappropriate C. difficile testing | Guidance on the management and treatment of Clostridium difficile infection. Public Health England. | Remind clinicians |
| (111) | Ireland | Primary Care | Quant RCT | Efficacy/Effectiveness | Antibiotics | Inappropriate use of antibiotics | Guidelines for antimicrobial prescribing in primary care in Ireland. | Distribute educational materials, develop educational materials, make training dynamic, remind clinicians, audit and provide feedback, |
| (112) | UK | Hospital | Quant Pre-post | Efficacy/Effectiveness | Antibiotics | Inappropriate use of antibiotics | NHS Lothian Adult Antimicrobial Guidelines. | Remind clinicians, develop educational materials, develop and organize quality monitoring systems, conduct cyclical small tests of change |
| (113) | US | Anticoagulation clinic | Mixed Pre-post | Process evaluation | Lab tests | Inappropriate INR laboratory tests | Evidence-based management of anticoagulant therapy: Antithrombotic Therapy and Prevention of Thrombosis. By the American College of Chest Physicians Evidence-Based Clinical Practice Guidelines | Distribute educational materials, audit and provide feedback, make training dynamic. |
| (114) | US | Hospital | Quant Pre-post | Efficacy/Effectiveness | Stress ulcer prophylaxis | Inappropriate use of Stress ulcer prophylaxis | ACCF/ACG/AHA 2010 expert consensus document on the concomitant use of proton pump inhibitors and thienopyridines.  Guidelines for prevention of NSAID-related ulcer complications. From the American Journal of Gastroenterology. | distribute educational materials, make training dynamic, develop and organize quality monitoring systems, |
| (115) | Australia | Hospital | Quant Pre-post | Efficacy/Effectiveness | Antibiotics | Inappropriate use of antibiotics for non-severe CAP patients | Therapeutic Guidelines: Antibiotic. Therapeutic Guidelines Limited. | make training dynamic audit and provide feedback |
| (116) | US | Hospital | Quant Pre-post | Efficacy/Effectiveness | Imaging | Over-utilized, high-cost imaging studies (CT brain, CT c-spine and CT pulmonary embolism [PE]) | the Canadian Head Injury rules, the National Emergency X-Radiography Utilization Study (NEXUS) c-spine rules, the Pulmonary Embolism Rule-out Criteria (PERC) and Wells scores. | [ACCOUNTABILITY TOOL], change physical structure and equipment, |
| (117) | Israel | Aged Care | Quant Retrospective study of RCT | Efficacy/Effectiveness | PIM Elderly | Potentially inappropriate medications | STOPP (Screening Tool of Older Per-son’s Prescriptions) and START (Screening Tool to Alert Doctors to Right Treatment). | develop and organize quality monitoring systems |
| (118) | England | Primary Care | Quant Pre-post | Efficacy/Effectiveness | Antibiotics | Inappropriate use of antibiotics | Prescribing of antibiotics for self-limiting respiratory tract infections in  adults and children in primary care, National Institute for Health and Care Excellence.  Management of sore throat and indications for tonsillectomy. By the Scottish International Guidelines Network. | remind clinicians, conduct cyclical small tests of change, conduct local needs assessment |
| (119) | Ireland | Primary Care | Quant RCT | Cost Effectiveness | PIM Elderly | Potentially inappropriate medications in primary care | Beer’s criteria   STOPP (Screening Tool of Older Person’s Prescriptions) and START (Screening Tool to Alert doctors to Right Treatment). | conduct educational outreach, develop and organize quality monitoring systems, remind clinicians, develop educational materials, |
| (120) | Switzerland | Hospital | Quant Pre-post | Efficacy/Effectiveness | PIM Elderly | Potentially inappropriate medications | STOPP (Screening Tool of Older Person’s Prescriptions) and START (Screening Tool to Alert doctors to Right Treatment). | Conduct educational outreach |
| (121) | Switzerland | Primary Care | Quant RCT | Efficacy/Effectiveness | Antibiotics | Inappropriate use of antibiotics | German Society for General Medicine and Family Medicine & British National Institute of Clinical Excellence (NICE) guidelines | Audit and provide feedback |
| (122) | US | ? | Quant Pre-post | Efficacy/Effectiveness | Imaging | Inappropriate imaging | Not covered by guidelines, of uncertain appropriateness, or inappropriate according to professional society guidelines that had been preselected by CMS, primarily from the American College of Radiology and the American College of Cardiology. | Remind Clinicians, audit and provide feedback |
| (123) | US | Multiple settings | Mixed Case study | Process evaluation | Imaging | Conducting a CT scan to evaluate suspected appendicitis in children | Choosing Wisely | remind clinicians, use advisory boards and workgroups, develop educational materials |
| (124) | India | Hospital | Quant Feasibility | Efficacy/Effectiveness | Surgical and non-surgical procedure | Inappropriate elective percutaneous coronary intervention | Appropriate use criteria for coronary revascularization focused update: A report of the American College of Cardiology Foundation Appropriate Use Criteria Task Force. | use other payment schemes, [ACCOUNTABILITY TOOL] |
| (125) | Denmark | Multiple settings | Quant pre-post | Efficacy/Effectiveness | Surgical and non-surgical procedures | Cataract surgery | Sundhedsstyrelsen (2013): National kliniskretningslinje for behandling af aldersbetingetgr a stær. | Distribute educational materials |
| (126) | US | Multiple settings | Quant pre-post | Efficacy/Effectiveness | Imaging | Inappropriate imaging | Haynes RB, Wilczynski NL. Effects of computerized clinical decision support systems on practitioner performance and patient outcomes: methods of a decision-maker-researcher partnership systematic review. Implement Sci 2010; 5:12  Archambault PM, Turgeon AF, Witteman HO, et al. Implementation and evaluation of a Wiki in-volving multiple stakeholders including patients in the promotion of best practices in trauma care: the WikiTrauma interrupted time series protocol. JMIR Res Protoc 2015; 4:e21  Djulbegovic B, Kumar A, Kaufman RM, Tobian A, Guyatt GH. Quality of evidence is a key determinant for making a strong guidelines recommendation. J Clin Epidemiol 2015; 68:727–737  Li Y, Kong N, Lawley M, Weiss L, Pagán JA. Advancing the use of evidence-based decision-making in local health departments with systems science methodologies. Am J Public Health 2015; 105(suppl 2):S217–S222 | Remind clinicians |
| (127) | US | Hospital | Quant Pre-post | Efficacy/Effectiveness | Antibiotics | Inappropriate use of high-risk antibiotics | CDC STD Treatment Guidelines.  ATS/IDSA Community Acquired Pneumonia in Adults: Guidelines for Management.  IDSA Practice Guidelines for Management of Skin and Soft Tissue Infections. | remind clinicians, [ACCOUNTABILITY TOOL], conduct educational meeting |
| (128) | Croatia | Hospital | Quant Pre-post | Efficacy/Effectiveness | Lab tests | Inappropriate laboratory testing | The third universal definition of myocardial infarction by a Writing group on behalf of the joint ESC/ACCF/AHA/WHF Task force for the universal definition of myocardial infarction. | Change physical structure and equipment |
| (129) | US | Primary Care | Quant RCT | Efficacy Effectiveness | Antibiotics | Inappropriate use of antibiotics  for nonspecific  upper respiratory tract infections, acute bronchitis, and  influenza. | Principles of Appropriate Antibiotic Use for Treatment of Nonspecific Upper Respiratory Tract Infections in Adults from Annals of Internal Medicine (Journal). | make training dynamic, remind clinicians, change physical structure and equipment, audit and provide feedback, [ACCOUNTABILITY TOOL] |
| (130) | China | Hospital | Quant Pre-post | Efficacy/Effectiveness | Stress ulcer prophylaxis | Inappropriate use of prophylactic acid suppressant in hepatobiliary surgical patients | ASHP Therapeutic Guidelines on Stress Ulcer Prophylaxis. | develop and organize quality monitoring systems, make training dynamic, develop educational materials, audit and provide feedback, |
| (131) | Canada | Hospital | Quant Quasi-experimental | Efficacy/Effectiveness | Antibiotics | Inappropriate use of antibiotics | IDSA guidelines | develop and organize quality monitoring systems, audit and provide feedback |
| (132) | US | Hospital | Quant Pre-post | Efficacy/Effectiveness | Lab tests | Inappropriate use of lab tests troponin and myoglobin/CK-MB for the diagnosis of AMI | Choosing Wisely | Distribute educational materials |
| (133) | Canada | Multiple settings | Quant Cohort (retrospective) | Efficacy/Effectiveness | Imaging | Overutilization of non-invasive cardiac imaging | ACCF/ASNC appropriateness criteria for single-photon emission computed tomography myocardial perfusion imaging (SPECTMPI): a report of the American College of Cardiology Foundation Quality Strategic Directions Committee Appropriateness Criteria Working Group and the American Society of Nuclear Cardiology endorsed by the American Heart Association.  Appropriate Use Criteria for Cardiac Radionuclide Imaging: A Report of the American College of Cardiology Foundation Appropriate Use Criteria Task Force.  Multimodality appropriate use criteria for the detection and risk assessment of stable ischemic heart disease: a report of the American College of Cardiology Foundation Appropriate Use Criteria Task Force. | Distribute educational materials |
| (134) | US | Hospital | Quant pre-post | Efficacy/Effectiveness | PIM Elderly | Inappropriate use of Glyburide | Beer’s criteria | remind clinicians, change physical structure and equipment |
| (135) | Netherland | Hospital | Quant Pre-post | Efficacy/Effectiveness | Antibiotics | Inappropriate use of antibiotics | National guidelines for antibiotic prescriptions. | Make training dynamic, identify and prepare champions, assess and redesign workflow, develop and organize quality monitoring systems, create new clinical teams, distribute educational materials, assess readiness and identify barriers and facilitators, tailor strategies |
| (136) | Singapore | Hospital | Quant pre-post | Efficacy/Effectiveness | Lab tests, Imaging | Inappropriate use of lab tests and imaging for preoperative investigations | Choosing wisely | remind clinicians, change physical structure and equipment |
| (137) | South Korea | Multiple settings | Quant Pre-post | Efficacy/Effectiveness | PIM Elderly | Potentially inappropriate medication | Public Announcement on Additional List of Age-Contraindicated Drugs. By the Ministry of Food and Drug Safety. | develop and organize quality monitoring systems |
| (138) | UK | Primary Care | Quant Pre-post | Efficacy/Effectiveness | Antipsychotics | Inappropriate prescribing of antipsychotics to patients diagnosed with dementia | Dementia: supporting people with dementia and their carers in health and social care. By the National Institute for Health and Care Excellence. | Distribute educational materials |
| (139) | US | Multiple settings | Quant Pre-post | Efficacy/Effectiveness | PIM Elderly | Potentially inappropriate medications | Beers Criteria | Develop and organize quality improvement systems |
| (140) | China | Primary Care | Quant RCT | Efficacy/Effectiveness | Antibiotics | Inappropriate use of antibiotics for pediatric outpatients | Guideline on Antibiotic Use in Clinical Practice. National Health and Family Planning Commission.  Respiratory tract infections (self-limiting): prescribing antibiotics. National Institute for Health and Care Excellence. | distribute educational materials, make training dynamic, audit and provide feedback, develop educational materials |
| (141) | US | Hospital | Quant Pre-post | Efficacy/Effectiveness | Opioids | Inappropriate opioid prescribing | Ohio guidelines for emergency and acute care facility opioid and other controlled substances (OOCS) prescribing. | Distribute educational materials |
| (142) | US | Hospital | Quant Pre-post | Efficacy/Effectiveness | Antibiotics | Inappropriate use of antibiotics post discharge | Practice guidelines for the diagnosis and management of skin and soft tissue infections. By the Infectious Diseases Society of America.  International clinical practice guidelines for the treatment of acute uncomplicated cystitis and pyelonephritis in women. By the Infectious Diseases Society of America and the European Society for Microbiology and Infectious Diseases.  American College of Gastroenterology guideline on the management of Helicobacter pylori infection.  Clinical practice guidelines for Clostridium difficile infection in adults. By the Society for Healthcare Epidemiology of America (SHEA) and the Infectious Diseases Society of America (IDSA).  Diagnosis, prevention, and treatment of catheter-associated urinary tract infection in adults: International clinical practice guidelines from the Infectious Diseases Society of America.  Infectious Diseases Society of America/American Thoracic Society consensus guidelines on the management of community-acquired pneumonia in adults.  Guidelines for the management of adults with hospital-acquired, ventilator-associated, and healthcare-associated pneumonia. By the American Thoracic Society and Infectious Diseases Society of America. | distribute educational materials, audit and provide feedback, develop and organize quality monitoring systems, stage implementation scale up |
| (143) | England | Hospital | Quant Pre-post | Efficacy/Effectiveness | Antibiotics | Inappropriate use of antibiotics, CXR, and nebulized or inhaled treatments in bronchiolitis. | Bronchiolitis: diagnosis and management of bronchiolitis in children, clinical guideline by the National Institute for Clinical Excellence. | conduct educational meeting, remind clinicians |
| (144) | US | Primary Care | Quant RCT | Cost effectiveness | Antibiotics | Inappropriate use of antibiotics | Practice guidelines for the diagnosis and management of group A streptococcal pharyngitis. Infectious Diseases Society of America.  Clinical practice guideline for the diagnosis and management of group A streptococcal pharyngitis. By the Infectious Diseases Society of America.  Diagnosis and management of cough executive summary: ACCP evidence-based clinical practice guidelines.  Infectious Diseases Society of America/American Thoracic Society consensus guidelines on the management of community-acquired pneumonia in adults. | change physical structure and equipment, [ACCOUNTABILITY TOOL], audit and provide feedback, |
| (145) | US | Multiple settings | Quant Pre-post | Efficacy/Effectiveness | Imaging | Early magnetic resonance imaging (MRI) for acute  low back pain (LBP) | Washington State Engrossed Substitute House Bill 2105: diagnostic  imaging workgroup.  ACR Appropriateness  Criteria on low back pain. | Use other payment schemes |
| (146) | US | Hospital | Quant Pre-post | Efficacy/Effectiveness | Antibiotics | Inappropriate use of antibiotics | Best practice policy statement on urologic surgery antimicrobial prophylaxis.  Clinical practice guidelines for antimicrobial prophylaxis in surgery. From the American Journal of Health Systems Pharmacology. | distribute educational materials, make training dynamic, use advisory boards and workgroups |
| (147) | Belgium | Primary Care | Quant A cluster randomized, factorial controlled trial (CRCT) | Efficacy/Effectiveness | Antibiotics | Inappropriate use of antibiotics for non-severe acute infections. | Belgian guide to anti-infectious treatment in the ambulatory care. | facilitating relay of clinical data, assess and redesign workflow, develop educational materials |
| (148) | Australia | Primary Care | Quant RCT | Efficacy/Effectiveness | Antibiotics | inappropriate use of antibiotics for upper respiratory tract infections (URTIs) and acute bronchitis/bronchiolitis | Antibiotic Expert Groups. Therapeutic Guidelines: Antibiotic. | Make training dynamic, tailor strategies, stage implementation scale up |
| (149) | Saudi Arabia | Hospital | Quant Pre-post | Efficacy/Effectiveness | PIM Elderly | Potentially inappropriate medications in geriatric patients | STOPP and Beers criteria | conduct educational meeting, conduct educational outreach, remind clinicians |
| (150) | Thailand | Hospital | Quant Pre-post | Efficacy/Effectiveness | Antibiotics | Inappropriate use of antibiotics for URIs. | Clinical practice guideline for the diagnosis and management of group A streptococcal pharyngitis. By the Infectious Diseases Society of America | Audit and provide feedback |
| (151) | US | Hospital | Quant pre-post | Efficacy/Effectiveness | bronchodilators, Imaging, lab tests | Routine use of bronchodilators, chest radiographs, or respiratory viral testing in children with a clinical diagnosis of bronchiolitis. | American Academy of Pediatrics’ Subcommittee on Diagnosis and Management of Bronchiolitis. Diagnosis and management of bronchiolitis.  American Academy of Pediatrics. Clinical practice guideline: the diagnosis, management, and prevention of bronchiolitis. | audit and provide feedback, obtain formal commitment, remind clinicians, changes in physical structure and equipment, distribute educational materials, make training dynamic, develop educational materials, conduct educational meeting |
| (152) | Ireland | Primary Care | Quant RCT | Efficacy/Effectiveness | PIM Elderly | Potentially inappropriate medication | Beer’s criteria, the STOPP criteria | conduct educational outreach, remind clinicians, develop educational materials |
| (153) | Ireland | Primary Care | Quant RCT | Efficacy/Effectiveness | PIM Elderly | Potentially inappropriate medication | Beer’s criteria, the STOPP criteria | conduct educational outreach, remind clinicians, develop educational materials |
| (154) | New Zealand | Hospital | Mixed Pre-post | Efficacy/Effectiveness | Obsetrics | The use of CTG monitoring, for assessment and  screening on admission to the maternity unit (admission  CTG) | American College of Nurse-Midwives (ACNM): Intermittent auscultation for  intrapartum fetal heart rate surveillance  American College of Obstetricians & Gynecologists (ACOG): Intrapartum  fetal heart rate monitoring. Practice Bulletin.  Intrapartum Care of Healthy Women and Their Babies During Childbirth, National Collaborating  Centre for Women’s and Children’s Health. National Institute for Health and Care Excellence.  New Zealand College of Midwives (NZCOM): Foetal Monitoring in Labour,  NZCOM Consensus Statement.  MIDIRS: Informed Choice for Professionals - Fetal Heart Rate Monitoring in Labour.  Royal Australian and New Zealand College of Obstetricians and  Gynaecologists (RANZCOG): Intrapartum Fetal Surveillance. Clinical Guidelines.  Royal College of Midwives (RCM): Evidence Based Guidelines for Midwifery-Led  Care in Labour.  Royal College of Obstetricians and Gynaecologists (RCOG): The Use of  Electronic Fetal Monitoring. The Use & Interpretation of Cardiotocography in  Intrapartum Fetal Surveillance, Evidenced-Based Clinical Guideline Number 8. | remind clinicians, make training dynamic, develop educational materials |
| (155) | US | Hospital | Quant Cohort | Efficacy/Effectiveness | ESAs | Inappropriate use of  erythropoiesis-stimulating agents (ESAs). | Healthcare Series MICROMEDEX. MICROME DEX Healthcare Series. Greenwood Village, Colo:  MICROMEDEX; 2011. | develop educational material, [BLACK BOX WARNING], use other payment schemes, |
| (156) | US | Aged Care | Quant Pre-post | Efficacy/Effectiveness | Antipsychotics | Inappropriate use of antipsychotic medications in the nursing home | Centers for Medicare and Medicaid Services. National Partnership to improve dementia care in nursing homes. | Assess and redesign workflow, revise professional roles, develop educational materials |
| (157) | US | Hospital | Quant Cohort | Efficacy/Effectiveness | Stress ulcer prophylaxis | Inappropriate utilization of Stress ulcer prophylaxis | 2010 expert consensus document on the concomitant use of proton pump inhibitors and thienopyridines: a focused update of the  ACCF/ACG/AHA 2008 expert consensus document on reducing the gastrointestinal risks of antiplatelet therapy and NSAID use. | use advisory boards and workgroups, distribute educational materials, make training dynamic, develop educational materials, remind clinicians, conduct educational outreach, develop and implement tools for quality monitoring |
| 2^nd^ |  |  |  |  |  |  |  |  |

| (158) | USA | Hospital | Quant Quasi-experimental | Efficacy/Effectiveness | Lab tests | lab testing for non-critically ill hospitalized COVID-19 patient | Choosing Wisely | change physical structure and equipment |
| --- | --- | --- | --- | --- | --- | --- | --- | --- |
| (159) | USA | Aged Care | Qual Cross-sectional | Process evaluation | Anti-psychotics | Inappropriate prescribing of psychotropic medication | Centers for Medicare and Medicaid Services. (2019). National partnership to improve dementia care in nursing homes: Antipsychotic medication use data report (April 2019)  Bowers, L. A. (2019, October 4). Increasing assisted living regulation is a trend, NCAL says. McKnight’s Senior Living. | identify and prepare champions, develop and organize quality monitoring systems, change physical structure and equipment |
| (160) | USA | Hospital | Quant Pre-post | Efficacy/Effectiveness | Other^1^ |  | Beers Criteria | [policy and regulation], change physical structure and equipment, develop and organize quality monitoring systems, make training dynamic, develop educational materials, audit and provide feedback |
| (161) | Denmark | Hospital | Quant RCT | Feasibility | PIM elderly | Potentially inappropriate medications | STOPP (Screening Tool of Older Person’s Prescriptions) and the Danish deprescribing list | develop and organize quality monitoring systems, [communication tool] |
| (162) | India | Hospital | Quant Pre-post | Efficacy/Effectiveness | Antibiotics | Inappropriate use of antibiotics | Surviving sepsis campaign: International guidelines for management of severe sepsis and septic shock: 2012. | audit and provide feedback, make training dynamic |
| (163) | Malaysia | Hospital | Quant Pre-post | Efficacy/Effectiveness | PIM elderly | Potentially inappropriate medications | STOPP (Screening Tool of Older Person’s Prescriptions) and START (Screening Tool to Alert doctors to Right Treatment) criteria | conduct educational outreach visits, remind clinicians |
| (164) | Japan | university hospital | Quant Pre-post | Efficacy/Effectiveness | Antibiotics | Inappropriate use of antibiotics | AURORA 2016 report by the Australian Commission on Safety and Quality in Health Care (ACSQHC) | conduct educational meeting, audit and provide feedback, provide clinical supervision |
| (165) | USA | Primary Care | Quant Pre-post | Efficacy/Effectiveness | Other^1^ | Inappropriate use of opioids | State of Wisconsin Medical Board Opioid Prescribing Recommendations | conduct educational meeting, remind clinicians, |
| (166) | India | Hospital | Quant Pre-post | Efficacy/Effectiveness | Antibiotics | Inappropriate use of antibiotics | National Guidelines developed by National Centre for Disease Control | develop educational materials |
| (167) | Canada | Aged Care | Quant RCT | Efficacy/Effectiveness | PIM elderly | Potentially inappropriate medications | Beer’s criteria | develop and organize quality monitoring system |
| (168) | USA | Hospital | Quant Pre-post | Efficacy/Effectiveness, Cost-effectiveness | Lab tests | Duplicate laboratory tests | Choosing wisely, American college of Chest Physicians and American Thoracic Society | develop and organize quality monitoring system |
| (169), | USA | Hospital | Quant Quasi-experimental | Process evaluation | Imaging, Antibiotics, Other^1^, Lab tests | Medical interventions discouraged by the AAP bronchiolitis guideline | the American Academy of Pediatrics (AAP) clinical practice guideline | distribute educational materials, change physical structure and equipment, conduct educational meeting, develop educational materials, audit and provide feedback |
| (170) | Australia | Primary Care | Mixed Pre-post | Feasibility | Antibiotics | Inappropriate use of antibiotics | Australia’s National Antimicrobial Resistance Strategy (Australian Government Department of Health) | remind clinicians, audit and provide feedback, conduct educational meeting |
| (171) | Multiple countries | Hospital | Mixed RCT | Efficacy/Effectiveness | PIM elderly | Potentially inappropriate medications | STOPP (Screening Tool of Older Person’s Prescriptions) and START (Screening Tool to Alert doctors to Right Treatment) criteria | develop and organize quality monitoring systems, remind clinicians, [communication tool] |
| (172) | USA | Hospital | Quant Pre-post | Efficacy/Effectiveness | Antibiotics | Inappropriate use of antibiotics | IDSA Clinical Practice Guidelines | develop and organize quality monitoring system, conduct educational meeting |
| (173) | Germany | Hospital | Quant Pre-post | Efficacy/Effectiveness | Antibiotics | Inappropriate use of antibiotics | Infectious Diseases Society of America and the Society for Healthcare Epidemiology of America guidelines for developing an institutional program to enhance antimicrobial stewardship | distribute educational materials, remind clinicians, develop educational materials, audit and provide feedback, conduct educational outreach, assess and redesign workflow |
| (174) | United Kingdom | Health Care System | Quant Quasi-experimental | Efficacy/Effectiveness | Antibiotics | Inappropriate use of antibiotics | NICE guidelines | alter incentive/allowance structures |
| (175) | Spain | Aged Care | Quant Pre-post | Efficacy/Effectiveness | Anti-psychotics | Antipsychotic treatment in institutionalized dementia patients | Guidelines from Ministry of Health Malaysia, Malaysian Psychiatric Association, Academy of Medicine Malaysia and Malaysian Society of Neurosciences 2009. Management of dementia.   Canadian Consensus Conference (3rd), 2007. Diagnosis and treatment of dementia. | distribute educational materials, |
| (176) | Australia | Aged Care | Quant Quasi-experimental | Efficacy/Effectiveness | Anti-psychotics | Inappropriate use of antipsychotic medications | Royal Australian and New Zealand College of Psychiatrists The Use of Antipsychotics in Residential Aged Care. Clinical Recommendations (2011)   The Royal Australian & New Zealand College of Psychiatrists Assessment and Management of People with Behavioural and Psychological Symptoms of Dementia (BPSD)—A Handbook for NSW Health Clinicians (2013) | identify and prepare champions, make training dynamic, conduct educational outreach, distribute educational materials. |
| (177) | USA | Hospital | Quant Quasi-experimental | Efficacy/Effectiveness | Antibiotics | Inappropriate use of antibiotics | Guidelines from California Medical Association’s Alliance Working for Antibiotic Resistance Education program | conduct educational meeting |
| (178) | Australia | Primary Care | Quant Cohort | Efficacy/Effectiveness | Acid suppression medication^2^ | Inappropriate PPI prescriptions | NICE guideline; Australian Therapeutic Guidelines | audit and provide feedback, conduct educational meeting, develop educational materials, |
| (179) | USA | Hospital | Quant Pre-post | Efficacy/Effectiveness, Cost-effectiveness | Other^1^ | Inappropriate albumin use in non-critically ill patients | American Association for the Study of Liver Diseases. Introduction to the revised American Association for the Study of Liver Diseases Practice Guideline management of adult patients with ascites due to cirrhosis 2012.   Guidelines on the use of therapeutic apheresis in clinical practice-evidence-based approach from the Writing Committee of the American Society for Apheresis: the seventh special issue.   Surviving sepsis campaign: international guidelines for management of sepsis and septic shock: 2016. | develop and organize quality monitoring system, distribute educational materials, conduct educational meeting, [accountability tool] |
| (180) | USA | Hospital | Quant Pre-post | Efficacy/Effectiveness, Cost-effectiveness | Other^1^ | Inappropriate use of albumin | American Association Liver Diseases Practice Guideline 2012  Guidelines on the use of therapeutic apheresis in clinical practice-evidence-based approach from the Writing Committee of the American Society for Apheresis   Surviving sepsis campaign: international guidelines for management of sepsis and septic shock: 2016. | distribute educational materials, [accountability tool], develop and organize quality monitoring system, audit and provide feedback, |
| (181) | USA | Primary Care | Quant Quasi-experimental | Efficacy/Effectiveness | Antibiotics | Inappropriate use of antibiotics | Infectious Diseases Society of America, 2012.   IDSA clinical practice guideline for acute bacterial rhinosinusitis in children and adults  Practice guidelines for the diagnosis and management of skin and soft tissue infections: 2014 update by the Infectious Diseases Society of America  Infectious Diseases Society of America guidelines for the diagnosis and treatment of asymptomatic bacteriuria in adults  International clinical practice guidelines for the treatment of acute uncomplicated cystitis and pyelonephritis in women: a 2010 update | conduct educational meeting, remind clinicians, |
| (182) | United Kingdom | Primary Care | Mixed Cross-sectional | Feasibility | PIM elderly | Potentially inappropriate medications | NICE clinical guidelines  STOPP (Screening Tool of Older Person’s Prescriptions) and START (Screening Tool to Alert doctors to Right Treatment) criteria | develop educational materials, develop and organize quality monitoring systems |
| (183) | Spain | Hospital | Quant Quasi-experimental | Efficacy/Effectiveness | Acid suppression medication^2^ | chronic PPI use in hospitalized patients | Farrell B, Pottie K, Thompson W et al (2017) Deprescribing proton pump inhibitors: evidence-based clinical practice guideline. Can Fam Physician 63(5):354–364  Bytzer P (2018) Deprescribing proton pump inhibitors: why, when and how. Med J Aust 209(10):436–438   Boghossian TA, Rashid FJ, Thompson W et al (2017) Deprescribing versus continuation of chronic proton pump inhibitor use in adults. Cochrane Database Syst Rev. | develop educational materials |
| (184) | USA | Primary Care | Quant RCT | Efficacy/Effectiveness | PIM elderly | Potentially inappropriate medications | Beers Criteria  Recommendations from the American Geriatrics Society (AGS), Centers for Medicare and Medicaid Services, and the National Committee for Quality Assurance | develop and organize quality monitoring system, change physical structure and equipment, develop educational materials |
| (185) | USA | Hospital | Quant RCT | Efficacy/Effectiveness | PIM elderly | Potentially inappropriate medications | US Food and Drug Administration | remind clinicians, develop and organize quality monitoring system, |
| (186) | Spain | Primary Care | Quant Pre-post | Cost-effectiveness | PIM elderly | Potentially inappropriate medications | STOPP (Screening Tool of Older Person’s Prescriptions) and START (Screening Tool to Alert doctors to Right Treatment) criteria | develop and organize quality monitoring systems, remind clinicians |
| (187) | Spain | Hospital | Quant Quasi-experimental | Efficacy/Effectiveness | Surgical and non-surgical procedures | Inappropriate venous thromboembolism (VTE) prophylaxis | Prevención de la Enfermedad Tromboembólica Venosa en Cirugía General y del Aparato Digestivo [Guidelines from Spanish Association of Surgery]  American College of Chest Physicians Evidence-Based Clinical Practice Guidelines.   European guidelines on perioperative venous thromboembolism prophylaxis: executive summary by the VTE Guidelines Task Force. | audit and provide feedback |
| (188) | Ireland | Primary Care | Quant Quasi-experimental | Feasibility | PIM elderly | Potentially inappropriate medications | World Health Organization (WHO). Medication safety in polypharmacy. | develop and organize quality monitoring system |
| (189) | Switzerland | Aged Care | Quant RCT | Efficacy/Effectiveness | PIM elderly | Potentially inappropriate medications | Beer’s criteria and the Norwegian General Practice – Nursing Home criteria (NORGEP-NH) | develop and organize quality monitoring systems, audit and provide feedback, assess and redesign workflow. Conduct cyclical small tests of change, use advisory board and workgroups |
| (190) | France | Hospital | Quant Pre-post | Process evaluation, Cost-effectiveness | Antibiotics | Inappropriate use of antibiotics | Société française d’hygiène hospitalière. Surveiller et prévenir les infections associées aux soins (2010). [Guidelines from French Society for Hospital Hygiene] | distribute educational materials, change physical structure and equipment, develop educational materials |
| (191) | Australia | Aged Care | Qual Pre-post | Process evaluation | Anti-psychotics | Inappropriate use of psychotropic medication | The Use of Antipsychotics in Residential Aged Care. Clinical Recommendations. | use train-the-trainer strategies |
| (192) | USA | Hospital | Quant Pre-post | Efficacy/Effectiveness | Other^1^ | Inappropriate opioid prescription | Surgeon General’s Advisory on Naloxone and Opioid Overdose, US Department of Health & Human Services  CDC Guideline for Prescribing Opioids for Chronic Pain | change physical structure and equipment, conduct educational meeting, distribute educational materials |
| (193) | Thailand | Hospital | Quant Quasi-experimental | Efficacy/Effectiveness | PIM elderly | Potentially inappropriate medications | Beer’s criteria | develop and organize quality monitoring systems |
| (194) | USA | Hospital | Quant Pre-post | Efficacy/Effectiveness | PIM elderly | Potentially inappropriate medications | Beer’s criteria | develop and organize quality monitoring systems |
| (195) | USA | Primary Care | Mixed Cross-sectional | Process evaluation | PIM elderly | Potentially inappropriate medications | Beers Criteria | develop and organize quality monitoring systems |
| (196) | Spain | Primary Care | Quant Quasi-experimental | Efficacy/Effectiveness | PIM elderly | Potentially inappropriate medications | STOPP (Screening Tool of Older Person’s Prescriptions) and START (Screening Tool to Alert doctors to Right Treatment) criteria  Beer’s criteria | remind clinicians, [COMMUNICATION TOOL] |
| (197) | Canada | Aged Care | Quant Cohort | Efficacy/Effectiveness | Anti-psychotics | Inappropriate antipsychotic prescribing | STOPP (Screening Tool of Older Person’s Prescriptions) and START (Screening Tool to Alert doctors to Right Treatment) criteria  Beers Criteria | conduct educational meeting, audit and provide feedback, use advisory boards and workgroups, develop and organize quality monitoring systems |
| (198) | Canada | Aged Care | Qual Cross-sectional | Process evaluation | Anti-psychotics | Inappropriate antipsychotic prescribing | Guidelines from Institut national d’excellence en sant ́e et en services sociaux (INESSS) [National Institute of Excellence in Health and Social Services (INESSS), 2017]. | make training dynamic, develop educational materials, conduct educational outreach, create new clinical teams, audit and provide feedback. |
| (199) | USA | Hospital | Quant Quasi-experimental | Efficacy/Effectiveness | Antibiotics | Inappropriate use of antibiotics | World Health Organization. Antibiotic resistance: Global report on surveillance, 2014.   Centers for Disease Control and Prevention. Antibiotic resistance threats in the United States, 2019.  United Nations. Draft political declaration of the high-level meeting on the General Assembly on antibiotic resistance. | distribute educational materials, develop educational materials, use advisory boards and workgroups, obtain formal commitment, |
| (200) | USA | Primary Care | Quant Quasi-experimental | Efficacy/Effectiveness | Antibiotics | Inappropriate prescribing of antibiotics | ACCP evidence-based clinical practice guidelines | distribute educational materials, develop educational materials, use advisory boards and workgroups, obtain formal commitment, Organize clinician implementation team meetings |
| (201) | USA | Hospital | Quant Pre-post | Efficacy/Effectiveness | Antibiotics | Inappropriate prescribing of antibiotics for acute respiratory tract infections | Centers for Disease Control and Prevention. Antibiotic prescribing and use in the US, antibiotic use in outpatient settings. 2017. | conduct educational meeting, develop educational materials, audit and provide feedback, |
| (202) | Ireland | Hospital | Quant RCT | Efficacy/Effectiveness, Cost-effectiveness | PIM elderly | Potentially inappropriate medications | STOPP (Screening Tool of Older Person’s Prescriptions) Frail criteria | develop and organize quality monitoring systems, |
| (203) | USA | Hospital | Quant Case study | Efficacy/Effectiveness, Cost-effectiveness | Lab tests | Fecal occult blood tests (FOBTs) for hospitalized patients | Choosing Wisely  US Preventive Services Task Force. Screening for colorectal cancer: recommendation and rationale  Guidelines for colonoscopy surveillance after screening and polypectomy: a consensus update by the US Multi-Society Task Force on Colorectal Cancer. | develop educational materials, conduct educational outreach visits. Conduct local needs assessment, tailor strategies |
| (204) | India | Primary Care | Quant Pre-post | Efficacy/Effectiveness | Antibiotics | Inappropriate use of antibiotics | Barton E, Spencer R. URTIs: recommended diagnosis and treatment in general practice. Prescriber 2011; 22:23–36. 10.1002/psb.743 | audit and provide feedback, make training dynamic, conduct educational outreach, |
| (205) | Switzerland | Hospital | Quant Quasi-experimental | Efficacy/Effectiveness | Acid suppression medication^2^ | Inappropriate PPI prescriptions | Choosing Wisely  P.O. Katz, L.B. Gerson, M.F. Vela. Guidelines for the diagnosis and management of gastroesophageal reflux disease  Canadian healthcare network. Top 100 drugs | conduct educational meeting, develop educational materials, audit and provide feedback, |
| (206) | Switzerland | Hospital | Quant Pre-post | Efficacy/Effectiveness, Cost-effectiveness | Other^1^ | Inappropriate prescribing of Benzodiazepines | Smarter medicine: la liste «Top 5» pour le secteur hospitalier. [Guidelines by Swiss Society of General Internal Medicine that builds on Choosing Wisely campaign] | conduct educational meeting, audit and provide feedback, remind clinicians, develop educational materials |
| (207) | United Kingdom | Aged Care | Quant RCT | Efficacy/Effectiveness, Cost-effectiveness | PIM elderly | Potentially inappropriate medications | STOPP (Screening Tool of Older Person’s Prescriptions) and START (Screening Tool to Alert doctors to Right Treatment) criteria | develop and organize quality monitoring systems |
| (208) | Canada | Hospital | Quant Pre-post | Efficacy/Effectiveness | Imaging, Lab tests, Other^1^ | Low-value bronchiolitis management practices | Clinical practice guideline: the diagnosis, management, and prevention of bronchiolitis; American Academy of Pediatrics guidelines | audit and provide feedback, develop educational materials, make training dynamic, obtain formal commitment |
| (209) | USA | Hospital | Quant Quasi-experimental | Efficacy/Effectiveness | Antibiotics | Inappropriate prescribing of antibiotics | Choosing Wisely  Centers for Disease Control and Prevention (CDC). Be Antibiotics Aware: Smart Use, Best Care. | develop and organize quality monitoring system, assess and redesign workflow, develop educational materials, conduct educational meeting, identify and prepare champions, make training dynamic, remind clinicians, obtain formal commitment, audit and provide feedback, conduct educational outreach visits, change physical structure and equipment. Assess readiness and identify barriers and facilitators, conduct local needs assessment. |
| (210) | USA | Hospital | Quant Pre-post | Efficacy/Effectiveness | Other^1^ | Unnecessary telemetry monitoring | Practice standards for electrocardiographic monitoring in hospital settings by the American Heart Association  Update to Practice Standards for Electrocardiographic Monitoring in Hospital Settings: A Scientific Statement from the American Heart Association.  Telemetry monitoring guidelines for efficient and safe delivery of cardiac rhythm monitoring to noncritical hospital inpatients  Joint Commission on Accreditation of Healthcare Organizations. 2014 National Patient Safety Goal.  Choosing Wisely | assess and redesign workflow, conduct educational meeting, audit and provide feedback, alter incentive/allowance structures |
| (211) | USA | Hospital | Quant Pre-post | Efficacy/Effectiveness | Imaging | Inappropriate use of inpatient PET-CT | Choosing Wisely and Imaging Gently | [ACCOUNTABILITY TOOL], remind clinicians, conduct educational meeting, develop educational materials. |
| (212) | Sweden | Primary Care | Quant Quasi-experimental | Efficacy/Effectiveness | Antibiotics | Inappropriate use of antibiotics | Swedish RTI guidelines by the Public Health Agency of Sweden | alter incentive/allowance structures |
| (213) | USA | Hospital | Quant Quasi-experimental | Feasibility | Imaging | Inappropriate head CT utilization | Choosing Wisely | mandate change, conduct educational meeting, conduct educational outreach visits, [COMMUNICATION TOOL], remind clinicians, assess and redesign workflow, |
| (214) | Switzerland | Hospital | Quant Pre-post | Efficacy/Effectiveness, Cost-effectiveness | Lab tests | Inappropriate laboratory tests | Smarter Medicine/Choosing Wisely Switzerland | audit and provide feedback, make training dynamic |
| (215) | Malaysia | Hospital | Qual Quasi-experimental | Efficacy/Effectiveness | Other^1^ | Physical restraint use in hospital | Guidelines by the Center of Medicare and Medicaid Service 2017 | make training dynamic |
| (216) | Belgium | Aged Care | Quant Quasi-experimental | Efficacy/Effectiveness | Anti-psychotics | Inappropriate prescribing of Benzodiazepines in nursing homes | STOPP (Screening Tool of Older Person’s Prescriptions) and START (Screening Tool to Alert doctors to Right Treatment) criteria  Beers Criteria | conduct educational meeting, develop and organize quality monitoring systems, obtain formal commitment, conduct a local consensus discussion |
| (217) | Spain | Primary Care | Quant Pre-post | Efficacy/Effectiveness | Other^1^ | Inappropriate mirabegron treatment | EAU guidelines on urinary incontinence. 2018. | develop educational materials, audit and provide feedback |
| (218) | Spain | Hospital | Quant Pre-post | Efficacy/Effectiveness | PIM elderly | Potentially inappropriate medications | STOPP (Screening Tool of Older Person’s Prescriptions) criteria | develop and organize quality monitoring system |
| (219) | Canada | Hospital | Quant Cross-sectional | Efficacy/Effectiveness | PIM elderly | Potentially inappropriate medications | Beer’s criteria | develop educational materials, develop and organize quality monitoring systems |
| (220) | France | Primary Care | Quant Pre-post | Efficacy/Effectiveness | PIM elderly | Potentially inappropriate medications | Explicit criteria for determining inappropriate medication use in nursing home residents: UCLA Division of Geriatric Medicine  STOPP (Screening Tool of Older Person’s Prescriptions) criteria | develop educational materials, conduct educational meeting |
| (221) | Spain | Hospital | Quant/Cohort | Efficacy/Effectiveness | PIM Elderly | Potentially inappropriate medications | Baer criteria; START/STOPP | develop and organize quality assurance systems, assess and redesign workflow |
| (222) | USA | Hospital | Quant Pre-post | Efficacy/Effectiveness | Anti-psychotics | Inappropriate prescription of antipsychotics | Food and Drug Administration. Information for  Healthcare Professionals: Conventional Antipsychotics. | change physical structure and equipment |
| (223) | USA | Hospital | Quant Cohort | Efficacy/Effectiveness | Surgical and non-surgical procedures | Inappropriate use of urinary catheters | Centers for Disease Control and Prevention (CDC) guidelines | identify and prepare champions, audit and provide feedback |
| (224) | Norway | Aged Care | Quant RCT | Efficacy/Effectiveness | Other | Inappropriate use of antihypertensives | ESH/ESC guidelines for the management of arterial hypertension:  2014 evidence-based guideline for the management of high blood pressure in adults: report from the panel members appointed to the Eighth Joint National Committee | conduct educational meeting, identify and prepare champions, develop educational materials, develop and organize quality monitoring systems |
| (225) | Sweden | Hospital | Quant RCT | Efficacy/Effectiveness | PIM elderly | Potentially inappropriate medications | The National Board of Health and Welfare. Indicators for Evaluating the Quality of Older People’s Drug Therapy. | develop and organize quality monitoring system, conduct educational outreach visits |
| (226) | USA | Hospital | Quant Quasi-experimental | Efficacy/Effectiveness | Lab tests | Inappropriate HO-CDI diagnostic testing | The 2018 Infectious Diseases Society of America (IDSA) and Society for Healthcare Epidemiology of America (SHEA) CDI clinical practice guidelines | remind clinicians, |
| (227) | USA | Health Care System | Quant Cohort | Efficacy/Effectiveness, Cost-effectiveness | PIM elderly | Potentially inappropriate medications | Farrell B, Black C, Thompson W, et al. Deprescribing antihyperglycemic agents in older persons: evidence-based clinical practice guideline. Can Fam Physician. 2017;63(11):832-43. | develop and organize quality monitoring systems |
| (228) | The Netherlands | Primary Care | Quant Pre-post | Efficacy/Effectiveness | PIM elderly | Potentially inappropriate medications | Multidisciplinary Guideline Polypharmacy in the Elderly from Dutch General Practitioners, Dutch Geriatric Society, Dutch Order of Medical Specialists.  STOPP (Screening Tool of Older Person’s Prescriptions) and START (Screening Tool to Alert doctors to Right Treatment) criteria | develop and organize quality monitoring system |
| (229) | USA | Hospital | Quant Pre-post | Efficacy/Effectiveness | Antibiotics | Inappropriate use of antibiotics | Infectious Diseases Society of America guidelines for the diagnosis and treatment of asymptomatic bacteriuria in adults. | develop educational materials, distribute educational materials, make training dynamic |
| (230) | France | Aged Care | Quant Pre-post | Efficacy/Effectiveness | Other^1^ | Inappropriate prescribing of benzodiazepines | Procedures for stopping benzodiazepines and similar drugs in older patients. The French National Health Authority (HAS) guidelines. | develop and organize quality monitoring system, develop educational materials, |
| (231) | USA | Hospital | Quant Quasi-experimental | Efficacy/Effectiveness | Lab tests | Inappropriate laboratory testing | Choosing Wisely  Official American thoracic Society/American Association of critical care nurses/American College of chest physicians/Society of critical care medicine policy Statement: The choosing wisely top 5 list in critical care medicine | assess and redesign workflow, develop educational materials, remind clinicians. Use advisory boards and workgroups. |
| (232) | USA | Health Care System | Quant Quasi-experimental | Efficacy/Effectiveness | Other^1^ | Inappropriate opioid prescription | American College of Obstetricians Gynecologists (ACOG). Committee opinion No. 711: Opioid use and opioid use disorder in pregnancy. | [policy and regulations] |
| (233) | Japan | Hospital | Quant Quasi-experimental | Efficacy/Effectiveness | Antibiotics | Inappropriate use of antibiotics | World Health Organization (WHO). Global action plan on antimicrobial resistance.  Ministry of Health, Labor and Welfare. Antimicrobial resistance action plan | conduct educational outreach visits, develop and organize quality monitoring system, [ACCOUNTABILITY TOOL] |
| (234) | Australia | Primary Care | Quant Quasi-experimental | Efficacy/Effectiveness | PIM elderly | Potentially inappropriate medications | STOPP (Screening Tool of Older Person’s Prescriptions) and START (Screening Tool to Alert doctors to Right Treatment) criteria  Beers Criteria | develop and organize quality monitoring system, distribute educational materials |
| (235) | Kenya | Primary Care | Quant Pre-post | Efficacy/Effectiveness, Feasibility | Antibiotics | Inappropriate antibiotic prescription | Scottish Intercollegiate Guidelines Network (SIGN) Guideline No 88 2012  Urinary tract infections in pregnancy: old and new unresolved diagnostic and therapeutic problems.   Treatment of non-neurogenic male LUTS. European association of urology, 2017.   Clinical guidelines from Ministry of Medical Services & Ministry of Public Health and Sanitation | develop and implement tool for quality monitoring, develop educational materials |
| (236) | Australia | Primary Care | Quant Cohort | Feasibility | PIM elderly | Potentially inappropriate medications | Beer’s criteria | conduct educational meetings, develop educational materials |
| (237) | USA | Primary Care | Mixed Quasi-experimental | Efficacy/Effectiveness | imaging and Antibiotics | Unnecessary imaging for lower back pain and headache and inappropriate use of antibiotics | Choosing wisely | obtain formal commitment, remind clinicians, develop educational materials. |
| (238) | Netherlands | Hospital | Quant Pre-post | Efficacy/Effectiveness | Surgical and non-surgical procedures | Inappropriate use of peripheral intravenous catheters and urinary catheters | Diagnosis, prevention, and treatment of catheter-associated urinary tract infection in adults: 2009 international clinical practice guidelines from the Infectious Diseases Society of America | identify and prepare champions, audit and provide feedback, conduct educational meeting, develop educational materials, assess and redesign workflow. Assess readiness for change and identify barriers and facilitators. |
| (239) | USA | Hospital | Quant Quasi-experimental | Efficacy/Effectiveness | Imaging | Imaging for low back pain | National clinical guidelines for non-surgical treatment of patients with recent onset low back pain or lumbar radiculopathy   Diagnosis and treatment of low back pain: a joint clinical practice guideline from the American College of physicians and the American pain Society. | remind clinicians |
| (240) | Canada | Aged Care | Quant Pre-post | Efficacy/Effectiveness | Antibiotics | Inappropriate antibiotic use for long term care facility residents with asymptomatic bacteriuria | Infectious Diseases Society of America guidelines for the diagnosis and treatment of asymptomatic bacteriuria in adults  Choosing Wisely Canada  Guidelines for the Prevention and Treatment of Urinary Tract Infections (UTIs) in Continuing Care Settings. | audit and provide feedback, conduct educational meeting, develop educational materials, |
| (241) | Afghanistan | Primary Care | Quant RCT | Efficacy/Effectiveness | Antibiotics, Other^1^ | Inappropriate prescribing of antimalarials and antibiotics | World Health Organization (WHO). Universal access to malaria diagnostic testing — an operational manual. | conduct educational meeting, develop educational materials, changes in physical structure and equipment |
| (242) | USA | Hospital | Quant Cohort | Efficacy/Effectiveness | Acid suppression medication^2^ | Inappropriate PPI prescriptions | Food and Drug Administration. FDA Drug Safety Communication: Clostridium difficile -associated diarrhea can be associated with stomach acid drugs known as proton pump inhibitors (PPIs). | develop educational materials, audit and provide feedback, change physical structure and equipment, conduct educational meeting, develop and organize quality monitoring system, |
| (243) | China | Hospital | Quant Pre-post | Efficacy/Effectiveness, Cost-effectiveness | Antibiotics | Excessive use of antibiotics | WHO 2015. Antimicrobial Resistance: Draft Global Action Plan on Antimicrobial Resistance  WHO 2016. WHO Methodology for A Global Program on Surveillance of Antimicrobial Consumption | [policy and regulations] |
| (244) | USA | Health Care System | Quant Pre-post | Efficacy/Effectiveness | Acid suppression medication^2^ | Inappropriate PPI use | The risks and benefits of long-term use of proton pump inhibitors: expert review and best practice advice from the American Gastroenterological Association | conduct educational meeting, develop educational materials, distribute educational materials, make training dynamic |
| (245) | USA | Hospital | Quant Cohort | Efficacy/Effectiveness | Antibiotics | Inappropriate use of antibiotics | Clinical practice guidelines for Clostridium difficile infection in adults and children: 2017 update by the Infectious Diseases Society of America (IDSA) and Society for Healthcare Epidemiology of America (SHEA). | conduct educational meeting, remind clinicians, change physical structure and equipment |
| (246) | USA | Aged Care | Mixed Quasi-experimental | Efficacy/Effectiveness | PIM elderly | Potentially inappropriate medications | 2019 ACC/AHA Guideline on the Primary Prevention of Cardiovascular Disease: a report of the American College of Cardiology/American Heart Association Task Force on Clinical Practice Guidelines | [COMMUNICATION TOOL], conduct educational meeting, develop and organize quality monitoring system. Use advisory boards and workgroups |
| (247) | China | Hospital | Quant Pre-post | Efficacy/Effectiveness, Cost-effectiveness | Acid suppression medication^2^ | Inappropriate PPI use | Critical Appraisal of the Quality of Clinical Practice Guidelines for Stress Ulcer Prophylaxis. [national guidelines] | distribute educational materials, develop and organize quality monitoring systems, audit and provide feedback, |
| (248) | USA | Health Care System | Quant Pre-post | Efficacy/Effectiveness, Cost-effectiveness | Surgical and non-surgical procedures | Preoperative care for patients undergoing cataract surgery | Choosing Wisely | conduct educational meeting, develop and organize quality monitoring system |
| (249) | Greece | Hospital | Quant Quasi-experimental | Efficacy/Effectiveness | Other^1^ | Inappropriate use of antifungals | Practice Guidelines for the Diagnosis and Management of Aspergillosis: 2016 Update by the Infectious Diseases Society of America   ECIL-6 Guidelines for the treatment of invasive candidiasis, aspergillosis and mucormycosis in leukemia and hematopoietic stem cell transplant patients | develop educational materials, distribute educational materials, conduct educational meeting |
| (250) | Canada | Primary Care | Quant RCT | Efficacy/Effectiveness, Process evaluation | PIM elderly | Potentially inappropriate medications | Beers Criteria | develop educational materials, |
| (251) | USA | Hospital | Quant Pre-post | Efficacy/Effectiveness, Cost-effectiveness | Acid suppression medication^2^ | inappropriate use of stress ulcer prophylaxis | American Society of Health-System Pharmacists therapeutic guidelines on stress ulcer prophylaxis | develop and organize quality monitoring system. conduct local needs assessment |
| (252) | USA | Primary Care | Quant Pre-post | Efficacy/Effectiveness | Antibiotics | Inappropriate antibiotic prescribing | High Value Care Task Force of the American College of Physicians and for the Centers for Disease Control and Prevention.  IDSA clinical practice guideline for acute bacterial rhinosinusitis in children and adults  Centers for Disease Control and Prevention. Appropriate antibiotic use: community. | remind clinicians |
| (253) | Canada | Hospital | Quant Quasi-experimental | Efficacy/Effectiveness | PIM elderly | Potentially inappropriate medications | Beers Criteria  Screening Tool of Older People's Prescriptions (STOPP) criteria  Choosing Wisely | develop and organize quality monitoring systems, develop educational materials |
| (254) | USA | Primary Care | Mixed Quasi-experimental | Efficacy/Effectiveness | PIM elderly | Potentially inappropriate medications | STOPP (Screening Tool of Older Person’s Prescriptions) and START (Screening Tool to Alert doctors to Right Treatment) criteria  Beers Criteria  Standards of medical care in diabetes--2014. Diabetes Care.  Evidence-based guideline from the Eighth Joint National Committee  American College of Cardiology/American Heart Association Task Force on Practice Guidelines  American Geriatrics Society Guidelines for Improving the Care of Older Adults with Diabetes Mellitus | develop and organize quality monitoring system, conduct educational meeting, make training dynamic |
| (255) | USA | Hospital | Quant Cross-sectional | Process evaluation | Other^1^ | Use of physical restraints | Clinical practice from the American College of Critical Care Medicine Task Force 2001–2002 | create a learning collaborative, distribute educational materials, develop educational materials |
| (256) | Australia | Primary Care | Quant Cohort | Cost-effectiveness | Imaging | Inappropriate CT scans and X-rays of the low back | Choosing Wisely Australia initiative, The Royal Australian and New Zealand College of Radiologists and the Australian Physiotherapy Association | audit and provide feedback, change physical structure and equipment, remind clinicians, develop educational materials, |
| (257) | Canada | Hospital | Quant Cohort | Efficacy/Effectiveness | Antibiotics | Inappropriate processing of midstream urine (MSU) cultures/inappropriate antibiotic treatment of asymptomatic bacteriuria | Clinical practice guideline for the management of asymptomatic bacteriuria: 2019 update by the Infectious Diseases Society of America. Screening for asymptomatic bacteriuria in adults: US Preventive Services Task Force recommendation statement. | [ACCOUNTABILITY TOOL] |
| (258) | USA | Hospital | Mixed Pre-post | Feasibility | Acid suppression medication^2^ | Inappropriate PPI use | Evidence-based guidelines from the Canadian Family Physician | develop and organize quality monitoring systems, conduct educational meetings, use advisory boards and workgroups, conduct cyclical small tests of change |
| (259) | Canada | Primary Care | Quant Pre-post | Efficacy/Effectiveness | Other^1^ | Inappropriate opioid prescribing | Canadian Guideline for Opioid for Chronic Pain by the National Pain Centre National Opioid Use Guideline Group.  Canadian Guideline for Safe and Effective Use of Opioids for Chronic Non-Cancer Pain 2010 | develop and organize quality monitoring systems, develop educational materials, identify and prepare champions, conduct educational meeting, remind clinicians, provide clinical supervision |
| (260) | USA | Hospital | Quant Pre-post | Efficacy/Effectiveness | Antibiotics | Inappropriate antibiotic use for asymptomatic bacteriuria | Clinical practice guideline for the management of asymptomatic bacteriuria: 2019 update by the Infectious Diseases Society of America | change physical structure and equipment, remind clinicians, conduct educational meeting, make training dynamic, develop educational materials |
| (261) | USA | Health Care System | Quant Quasi-experimental | Efficacy/Effectiveness | PIM elderly | Potentially inappropriate medications | Screening Tool of Older People's Prescriptions (STOPP) criteria   Beer’s criteria | develop educational materials |
| (262) | USA | Health Care System | Quant/Cohort | Efficacy/Effectiveness | Other | Granulocyte colony–stimulating factors (G-CSFs) | American Society of Clinical Oncology: 2006 Update of Recommendations for the Use of White Blood Cell Growth Factors: An Evidence-Based Clinical Practice Guideline | develop educational materials, assess and redesign workflow, develop and organize quality monitoring systems, [ACCOUNTABILITY TOOL] |
| (263) | Spain | Primary Care | Quant Quasi-experimental | Efficacy/Effectiveness | Antibiotics | Inappropriate use of antibiotics | World Health Organization (WHO). Antimicrobial resistance: global report on surveillance 2014 | develop educational materials, change physical structure and equipment, conduct educational outreach visits, audit and provide feedback |
| (264) | USA | Health Care System | Quant Quasi-experimental | Efficacy/Effectiveness | Imaging, Other^1^ | Inappropriate ordering of Complete blood counts (CBCs) and electrocardiograms (EKGs) | Choosing Wisely | distribute educational materials, develop educational materials, conduct educational meeting, make training dynamic, mandate change |
| (265) | New Zealand | Hospital | Qual Cross-sectional | Process evaluation | Lab tests | Routine pre-operative testing | Choosing Wisely  O’Neill F, Carter E, Pink N, Smith I. Routine preoperative tests for elective surgery: summary of updated NICE guidance | distribute educational materials, develop educational materials |
| (266) | United Kingdom | Hospital | Quant Cohort | Efficacy/Cost-effectiveness | Other^1^ | Inappropriate use of antifungals | ESCMID guideline for the diagnosis and management of Candida diseases 2012: non-neutropenic adult patients.  NICE. Antimicrobial Stewardship Quality Standard QS121. 2016. | distribute educational materials, change physical structure and equipment, develop educational materials, make training dynamic, identify and prepare champions |
| (267) | USA | Health Care System | Quant Pre-post | Efficacy/Effectiveness | Imaging | Inappropriate imaging | Choosing Wisely  American College of Radiology. ACR Appropriateness Criteria | make training dynamic, develop educational materials, audit and provide feedback, conduct local needs assessment |
| (268) | Spain | Hospital | Quant Quasi-experimental | Efficacy/Effectiveness | PIM elderly | Potentially inappropriate medications | STOPP (Screening Tool of Older Person’s Prescriptions) and START (Screening Tool to Alert doctors to Right Treatment) criteria | develop and organize quality monitoring systems, audit and provide feedback, develop educational materials, develop and implement tools for quality monitoring |
| (269) | USA | Primary Care | Quant RCT | Efficacy/Effectiveness | Anti-psychotics | Inappropriate prescribing of antipsychotic agents | Choosing Wisely,   FDA, 2016  Beers Criteria | audit and provide feedback |
| (270) | Spain | Primary Care | Quant Quasi-experimental | Efficacy/Effectiveness | PIM elderly | Potentially inappropriate medications | STOPP (Screening Tool of Older Person’s Prescriptions) and START (Screening Tool to Alert doctors to Right Treatment) criteria | develop and organize quality monitoring systems, develop educational materials, |
| (271) | Canada | Primary Care | Quant RCT | Cost-effectiveness | Other^1^ | Inappropriate use of NSAIDs | Beer’s criteria | develop educational materials, [COMMUNICATION TOOL] |
| (272) | Spain | Aged Care | Quant Cost-effectiveness/Pre-post | Cost-effectiveness | PIM elderly | Potentially inappropriate medications | STOPP (Screening Tool of Older Person’s Prescriptions) and START (Screening Tool to Alert doctors to Right Treatment) criteria  Beer’s criteria | develop and organize quality monitoring systems |
| (273) | Australia | Health Care System | Quant Cross-sectional | Efficacy/Effectiveness | Anti-psychotics | Inappropriate prescription of antipsychotics | Martins D, Khuu W, Tadrous M, et al.; Ontario Drug Policy Research Network. Impact of delisting high-strength opioid formulations from a public drug benefit formulary on opioid utilization in Ontario, Canada. Pharmacoepidemiol Drug Saf. 2019;28(5):726-733. | Alter patient/consumer fees, change physical structure and equipment |
| (274) | Argentina | Hospital | Quant Pre-post | Feasibility | PIM elderly | Potentially inappropriate medications | Beer’s criteria | develop educational materials, remind clinicians, identify and prepare champions, make training dynamic |
| (275) | Germany | Primary Care | Quant RCT | Efficacy/Effectiveness | PIM elderly | Potentially inappropriate medications | Boyd CM, Darer J, Boult C, et al.. Clinical practice guidelines and quality of care for older patients with multiple comorbid diseases. JAMA 2005; 294:716–24. | make training dynamic, develop and organize quality monitoring system, [COMMUNICATION TOOL] |
| (276) | India | Hospital | Quant Quasi-experimental | Feasibility | Other^1^ | Inappropriate prescribing of Benzodiazepines and Z drugs | Pottie et al., 2018 K. Pottie, W. Thompson, S. Davies, J. Grenier, C. Sadowski, V. Welch, ..., B. Farrell Evidence-based clinical practice guideline for deprescribing benzodiazepine receptor. Can Fam Physician, 64 (2018), pp. 339-351 | develop and organize quality monitoring system, [COMMUNICATION TOOL]. |
| (277) | Denmark | Hospital | Quant Pre-post | Efficacy/Effectiveness | PIM elderly | Potentially inappropriate medications | Beers Criteria  Improved Prescribing in the Elderly Tool (IPET)) | develop and organize quality monitoring systems, make training dynamic, |
| (278) | Australia | Primary Care | Quant Quasi-experimental | Efficacy/Effectiveness | Imaging | Clinical management decisions related to imaging and complex medicines for low back pain | International clinical guidelines by the American College of Physicians | remind clinicians, change physical structure and equipment |
| (279) | Canada | Hospital | Quant Cohort | Process evaluation/cost saving | Lab tests | Inappropriate laboratory testing | Choosing Wisely Canada   Clinical practice guidelines for hypothyroidism in adults: cosponsored by the American Association of Clinical Endocrinologists and the American Thyroid Association | change physical structure and equipment, [ACCOUNTABILITY TOOL], conduct educational meeting, develop educational materials, remind clinicians |
| (280) | China | Health Care System | Quant Quasi-experimental | Efficacy/Effectiveness, Cost-effectiveness | Antibiotics | Inappropriate prescribing of antibiotics | World Health Organization (WHO). The World medicines situation 2011: rational use of medicines. | [POLICY AND REGULATION], distribute educational materials, change physical structure and equipment, [ACCOUNTABILITY TOOL], develop and organize quality monitoring system |
| (281) | USA | Hospital | Quant Pre-post | Efficacy/Effectiveness, Cost-effectiveness | Lab tests, Other^1^ | ineffective testing and therapies in children with bronchiolitis | Choosing wisely  American Academy of Pediatrics Guideline | distribute educational materials, change physical structure and equipment, make training dynamic, develop educational materials, audit and provide feedback, alter incentive/allowance structure. Assess readiness and identify barriers and facilitators |
| (282) | USA | Hospital | Quant Quasi-experimental | Efficacy/Effectiveness | Surgical and non-surgical procedures | Early elective deliveries (EEDs) | The American College of Obstetricians and Gynecologists. Avoidance of Nonmedically Indicated Early-Term Deliveries and Associated Neonatal Morbidities. | audit and provide feedback, conduct educational meeting, create a learning collaborative |
| (282) | USA | Hospital | Mixed/Quasi-experimental | Efficacy/Effectiveness | Imaging | CXR ordering | An official American Thoracic Society/American Association of Critical-Care Nurses/American College of Chest Physicians/Society of Critical Care Medicine policy statement: the Choosing Wisely® top 5 list in critical care medicine.  ACR appropriateness criteria® routine chest radiography. | conduct educational meeting, audit and feedback, develop educational materials, use advisory boards and workgroups, conduct cyclical small tests of change, assess readiness and identify barriers and facilitators |
| (283) | Canada | Primary Care | Qual Quasi-experimental | Feasibility study | Acid suppression medication^2^ | inappropriate prescriptions of PPIs and benzodiazepines | Beers Criteria  STOPP (Screening Tool of Older Person’s Prescriptions) and START (Screening Tool to Alert doctors to Right Treatment) criteria | conduct educational meetings, develop educational materials, remind clinicians |
| (284) | Belgium | Hospital | Quant/Quasi-experimental | Efficacy/Effectiveness | PIM Elderly | Potentially inappropriate medications | RASP (Rationalization of  Home Medication by an Adjusted STOPP in Older Patients) list | provide clinical supervision, develop and organize quality monitoring systems |
| (285) | The Netherlands | Aged Care | Quant RCT | Efficacy/Effectiveness | PIM elderly | Potentially inappropriate medications | Guideline for problem behaviour of the Dutch Association of Elderly Care Physicians and Social Geriatricians (Verenso)  the Systematic Tool to Reduce Inappropriate Prescribing (STRIP)  the Screening Tool to Alert doctors to Right Treatment (START)   the Screening Tool of Older Person’s potentially inappropriate Prescriptions (STOPP) | develop and organize quality monitoring systems, conduct educational meetings, use advisory boards and workgroups |
| (286) | The Netherlands | Hospital | Quant Pre-post | Efficacy/Effectiveness | Other^1^ | Inappropriate Use of antithrombotic  agents | The European guidelines from the European Society of Cardiology (ESC), the European Association for Cardio-Thoracic Surgery (EACTS), European Respiratory  Society (ERS) and the European Society for Vascular Surgery (ESVS)  Dutch national guidelines. | remind clinicians, develop and organize quality monitoring system |
| (287) | The Netherlands | Primary Care | Quant Quasi-experimental | Efficacy/Effectiveness, cost-effectiveness | Lab tests | vitamin D and B12 tests | Choosing wisely | conduct educational meeting, audit and provide feedback, develop educational materials. |
| (288) | USA | Primary Care | Mixed Quasi-experimental | Efficacy/Effectiveness, Feasibility | PIM elderly | Potentially inappropriate medications | Beers Criteria  The National Committee for Quality Assurance. 2015 State of Healthcare Quality | conduct educational outreach visits, develop educational materials, remind clinicians, develop and organize quality monitoring systems, audit and provide feedback |
| (289) | Netherlands | Hospital | Quant | Feasibility | PIM elderly | Potentially inappropriate medications | the Systematic Tool to Reduce Inappropriate Prescribing (STRIP) method embedded in the Dutch multidisciplinary polypharmacy guideline (Dutch General Practitioners, Dutch Geriatric Society, Dutch Order of Medical Specialists, 2012) | develop and organize quality monitoring systems |
| (290) | Vietnam | Hospital | Quant Pre-post | Efficacy/Effectiveness | PIM Elderly | Potentially inappropriate medications | Beer’s criteria | conduct educational meeting, develop educational materials. |
| (291) | United Kingdom | Primary Care | Quant Quasi-experimental | Efficacy/Effectiveness | Antibiotics | Inappropriate prescribing of antibiotics | NICE guidelines | [POLICY AND REGULATION], distribute educational materials, develop educational materials, make training dynamic, use mass media, use data warehousing technique, [INTERNATIONAL COLLABORATION] |
| (292) | USA | Primary Care | Quant Pre-post | Efficacy/Effectiveness | Imaging | Inappropriate lumbar spine MRI for uncomplicated low back pain | Guidelines from the American College of Physicians and the American Pain Society  ACR Appropriateness Criteria Low Back Pain  Choosing Wisely | make training dynamic |
| (293) | USA | Hospital | Quant Quasi-experimental | Efficacy/Effectiveness | Imaging | inappropriate CXRs obtained for pediatric patients with acute asthma exacerbations | Guidelines for the diagnosis and management of asthma by the National Asthma Education and Prevention Program; National Heart, Lung, and Blood Institute. | assess and redesign workflow, develop educational materials, change physical structure and equipment, remind clinicians, conduct educational meeting, use advisory boards and workgroups, assess change readiness and identify barriers and facilitators |
| (294) | Belgium | Aged Care | Quant Quasi-experimental | Efficacy/Effectiveness, Feasibility | PIM elderly | Potentially inappropriate medications | The European Union (EU) (7)-PIM list  STOPP (Screening Tool of Older Person’s Prescriptions) and START (Screening Tool to Alert doctors to Right Treatment) criteria  Beer’s criteria | develop and organize quality monitoring systems |
| (295) | Spain | Aged Care | Quant Quasi-experimental | Efficacy/Effectiveness | PIM elderly | Potentially inappropriate medications | STOPP (Screening Tool of Older Person’s Prescriptions) and START (Screening Tool to Alert doctors to Right Treatment) criteria | develop and organize quality monitoring system, develop educational materials, conduct educational meeting |
| (296) | Australia | Primary Care | Quant Cross-sectional | Feasibility | Anti-psychotics | Anti-psychotic medication for patients with dementia | Wallace T, Chand R, Buck E, et al. ReBOC. Reducing behaviours of concern. A hands-on guide. A resource to assist those caring for people living with dementia. 2012; Glenside, South Australia: Alzheimer's Australia  Burns K, Jayasinha R, Tsang R, Brodaty H. Behaviour management: a guide to good practice. Managing behavioural and psychological symptoms of dementia. 2012; Sydney: Commonwealth of Australia, 188 p. | develop educational materials, Change physical structure and equipment |
| (297) | USA | Health Care System | Quant | Feasibility, Cost-effectiveness | PIM elderly | Potentially inappropriate medications | Beers Criteria  STOPP (Screening Tool of Older Person’s Prescriptions) and START (Screening Tool to Alert doctors to Right Treatment) criteria  Medication Appropriateness Index (MAI) | develop and organize quality monitoring system |
| (298) | USA | Primary Care | Quant Cross-sectional | Feasibility | PIM elderly | Potentially inappropriate medications | STOPP (Screening Tool of Older Person’s Prescriptions) and START (Screening Tool to Alert doctors to Right Treatment) criteria | develop and organize quality monitoring systems |
| (299) | Canada | Aged Care | Quant Quasi-experimental | Feasibility | PIM elderly | Potentially inappropriate medications | Beer’s criteria and the STOPP/START | Develop and organize quality monitoring systems, make training dynamic, develop educational materials. |
| (300) | Canada | Hospital | Quant Pre-post | Efficacy/Effectiveness | Other^1^ | Inappropriate use of sedatives | American Society of Geriatrics   Choosing Wisely | develop educational materials |
| (301) | Canada | Hospital | Quant Quasi-experimental | Efficacy/Effectiveness; feasibility | PIM elderly | Potentially inappropriate medications | American Society of Geriatrics  Choosing Wisely | develop educational materials |
| (302) | Canada | Primary Care | Quant Cohort | Efficacy/Effectiveness | Lab tests | Inappropriate ordering of thyroid-stimulating hormone test | Choosing Wisely Canada  the College of Family Physicians of Canada on thyroid function test | audit and provide feedback, conduct educational meeting |
| (303) | USA | Primary Care | Quant Case study | Feasibility | Imaging |  | Choosing Wisely  American Diabetes Association; European Association for the Study of Diabetes. Management of hyperglycemia in type 2 diabetes: a patient-centered approach: position statement of the American Diabetes Association (ADA) and the European Association for the Study of Diabetes (EASD).  Canadian Diabetes Association Clinical Practice Guidelines Expert Committee; Cheng AY Canadian Diabetes Association 2013 clinical practice guidelines for the prevention and management of diabetes in Canada  U.S. Department of Health and Human Services Office of Disease Prevention and Health Promotion National Action Plan for Adverse Drug Event Prevention.   U.S. Department of Veterans Affairs Management of diabetes mellitus in primary care (2017): VA/DoD clinical practice guidelines | develop and organize quality monitoring system, remind clinicians, develop educational materials, make training dynamic |
| (304) | Australia | Primary Care | Quant Quasi-experimental | Efficacy/Effectiveness | Antibiotics | Inappropriate antibiotic prescriptions for upper respiratory tract infections | AURA 2016: first Australian report on antimicrobial use and resistance in human health. Australian Commission on Safety and Quality in Health care (ACSQHC). | audit and provide feedback, make training dynamic, conduct educational meeting, develop educational materials |
| (305) | Australia | Health Care System | Quant Pre-post | Efficacy/Effectiveness | Acid suppression medication | reducing unnecessary prescribing of proton pump inhibitors (PPIs) | Choosing wisely Australia | audit and provide feedback, make training dynamic |
| (306) | China | Hospital | Quant Pre-post | Efficacy/Effectiveness, Cost-effectiveness, Feasibility | Acid suppression medication^2^ | Inappropriate PPI prescriptions | Chinese National Expert Committee on Rational Drug Use. Guidelines for Chinese Doctors and Pharmacists.  Deprescribing proton pump inhibitors: Evidence-based clinical practice guideline. | conduct educational outreach visits, conduct educational meetings, develop educational materials, |
| (307) | USA | Hospital | Quant RCT | Efficacy/Effectiveness | Antibiotics | Inappropriate antibiotic prescriptions | Presidential Advisory Council on Combating Antibiotic-resistant Bacteria. National Action Plan for Combating Antibiotic-resistant Bacteria. White House; 2015   Centers for Disease Control and Prevention. FastStats: Emergency Department Visits. | conduct educational meeting, identify and prepare champions, audit and provide feedback, develop educational materials, obtain formal commitment |
| (308) | China | Hospital | Quant Pre-post | Efficacy/Effectiveness | Acid suppression medication^2^ | Inappropriate PPI prescriptions | American gastroenterological association | conduct educational outreach visits, conduct educational meeting |
| (309) | China | Primary Care | Quant Quasi-experimental | Efficacy/Effectiveness | Antibiotics | Inappropriate prescribing of antibiotics | World Health Organization (WHO). How to investigate drug use in health facilities. Selected drug use indicators. | audit and provide feedback, conduct educational outreach visits |
| (310) | USA | Hospital | Mixed Quasi-experimental | Efficacy/Effectiveness | Imaging | CXR ordering | An official American Thoracic Society/American Association of Critical-Care Nurses/American College of Chest Physicians/Society of Critical Care Medicine policy statement: the Choosing Wisely® top 5 list in critical care medicine.  McComb BL, Chung JH, Crabtree TD, et al. ACR appropriateness criteria® routine chest radiography. | conduct educational meeting, audit and provide feedback, develop educational materials, Use advisory boards and workgroups. Conduct cyclical small tests of change, assess readiness and identify barriers and facilitators |

^1^Includes Bronchodilators (n=4), Opioids (n=4), Antimalarials (n=4), Benzodiazepines (n=3), Physical restraint (n=2), Echocardiography (n=2), Screening (n=2), Albumin (n=2), Antifungals (n=2), Sedatives (n=2), Corticosteroids (n=2), NSAIDs (n=2), Antihypertensives (n=1), SMBG (n=1), Antiplatelet therapy (n=1), Catheters (n=1), ANA (n=1), Colonoscopy (n=1), COXIB (n=1), Pulse oximetry (n=1), Erythropoiesis-stimulating agents (ESAs) (n=1), Antithrombotic agents (n=1), Z drugs (n=1), Domperidone (n=1), Cardiac testing (n=1), Complete blood counts (CBCs) (n=1), Electrocardiograms (EKGs) (n=1), Mirabegron (n=1), Telemetry (n=1)
^2^Includes Stress ulcer prophylaxis (n=5), Proton pump inhibitors (n=1)

1. Ip IK, Gershanik EF, Schneider LI, Raja AS, Mar W, Seltzer S, et al. Impact of IT-enabled intervention on MRI use for back pain. Am J Med. 2014;127(6):512-518.e1.

2. Iyengar K, Jain M, Thomas S, Dashora K, Liu W, Saini P, et al. Adherence to evidence based care practices for childbirth before and after a quality improvement intervention in health facilities of Rajasthan, India. BMC Pregnancy Childbirth. 2014;14:270.

3. Nausheen S, Hammad R, Khan A. Rational use of antibiotics--a quality improvement initiative in hospital setting. JPMA - J Pakistan Med Assoc. 2013;63(1):60–4.

4. Meeker D, Linder JA, Fox CR, Friedberg MW, Persell SD, Goldstein NJ, et al. Effect of Behavioral Interventions on Inappropriate Antibiotic Prescribing Among Primary Care Practices: A Randomized Clinical Trial. JAMA. 2016;315(6):562–70.

5. Alagiakrishnan K, Wilson P, Sadowski CA, Rolfson D, Ballermann M, Ausford A, et al. Physicians’ use of computerized clinical decision supports to improve medication management in the elderly – The Seniors Medication Alert and Review Technology intervention. Clin Interv Aging. 2016;11:73–81.

6. Andrew MK, Purcell CA, Marshall EG, Varatharasan N, Clarke B, Bowles SK. Polypharmacy and use of potentially inappropriate medications in long-term care facilities: does coordinated primary care make a difference? Int J Pharm Pract. 2018 Aug 1;26(4):318–24.

7. Cooper DL, Titler M, Struble L, Redman R. A multifaceted, evidence-based program to reduce inappropriate antibiotic treatment of suspected urinary tract infections. Ann Long-Term Care. 2017;25(2):36–43.

8. Latham I, Brooker D. Reducing anti-psychotic prescribing for care home residents with dementia. Nurse Prescr. 2017;15(10).

9. Hogli JU, Garcia BH, Skjold F, Skogen V, Smabrekke L. An audit and feedback intervention study increased adherence to antibiotic prescribing guidelines at a Norwegian hospital. BMC Infect Dis. 2016;16:96.

10. Clyne B, Cooper JA, Hughes CM, Fahey T, Smith SM, Team O-S study. A process evaluation of a cluster randomised trial to reduce potentially inappropriate prescribing in older people in primary care (OPTI-SCRIPT study). Trials. 2016;17(1):386.

11. Dalbak LG, Rognstad S, Melbye H, Stra, J. Changed terms for drug payment influenced GPs’ diagnoses and prescribing practice for inhaled corticosteroids. Eur J Gen Pract. 2013;19(2):106–10.

12. Jennings RM, Burtner JJ, Pellicer JF, Nair DK, Bradford MC, Shaffer M, et al. Reducing head CT use for children with head injuries in a community emergency department. Pediatrics. 2017;139(4).

13. Martins CM, da Costa Teixeira AS, de Azevedo LF, Sa LM, Santos PA, do Couto ML, et al. The effect of a test ordering software intervention on the prescription of unnecessary laboratory tests - a randomized controlled trial. BMC Med Informatics Decis Mak. 2017;17(1):20.

14. Sadowski BW, Lane AB, Wood SM, Robinson SL, Kim CH. High-Value, Cost-Conscious Care: Iterative Systems-Based Interventions to Reduce Unnecessary Laboratory Testing. Am J Med. 2017;130(9):1112.e1-1112.e7.

15. White DR, Hamilton KW, Pegues DA, Hanish A, Umscheid CA. The Impact of a Computerized Clinical Decision Support Tool on Inappropriate Clostridium difficile Testing. Infect Control Hosp Epidemiol. 2017;38(10):1204–8.

16. Klatte JM, Selvarangan R, Jackson MA, Myers AL. Reducing Overutilization of Testing for Clostridium difficile Infection in a Pediatric Hospital System: A Quality Improvement Initiative. Hosp Pediatr. 2016;6(1):9–14.

17. Melendez-Rosado J, Thompson KM, Cowdell JC, Sanchez Alvarez C, Ung RL, Villanueva A, et al. Reducing unnecessary testing: an intervention to improve resident ordering practices. Postgrad Med J. 2017;93(1102):476–9.

18. Robson J, Smithers H, Chowdhury T, Bennett-Richards P, Keene D, Dostal I, et al. Reduction in self-monitoring of blood glucose in type 2 diabetes: an observational controlled study in east London. Br J Gen Pract. 2015;65(633):e256-63.

19. Lin IB, Coffin J, O’Sullivan PB. Using theory to improve low back pain care in Australian Aboriginal primary care: a mixed method single cohort pilot study. BMC Fam Pract. 2016;17:44.

20. Garcia-Gollarte F, Baleriola-Julvez J, Ferrero-Lopez I, Cuenllas-Diaz A, Cruz-Jentoft AJ. An educational intervention on drug use in nursing homes improves health outcomes resource utilization and reduces inappropriate drug prescription. J Am Med Dir Assoc. 2014;15(12):885–91.

21. Corbi G, Gambassi G, Pagano G, Russomanno G, Conti V, Rengo G, et al. Impact of an Innovative Educational Strategy on Medication Appropriate Use and Length of Stay in Elderly Patients. Medicine (Baltimore). 2015;94(24):e918.

22. Angelidou A, Bell K, Gupta M, Tropea Leeman K, Hansen A. Implementation of a Guideline to Decrease Use of Acid-Suppressing Medications in the NICU. Pediatrics. 2017;140(6).

23. Sigmund AE, Stevens ER, Blitz JD, Ladapo JA. Use of Preoperative Testing and Physicians’ Response to Professional Society Guidance. JAMA Intern Med. 2015;175(8):1352–9.

24. Ackerman SL, Gonzales R, Stahl MS, Metlay JP. One size does not fit all: evaluating an intervention to reduce antibiotic prescribing for acute bronchitis. BMC Health Serv Res. 2013;13:462.

25. Bailey JE, Wan JY, Mabry LM, L, Y SH, Pope RA, et al. Does health information exchange reduce unnecessary neuroimaging and improve quality of headache care in the emergency department? J Gen Intern Med. 2013;28(2):176–83.

26. Bhatia RS, Milford CE, Picard MH, Weiner RB. An educational intervention reduces the rate of inappropriate echocardiograms on an inpatient medical service. Jacc Cardiovasc Imaging. 2013;6(5):545–55.

27. Brahmbhatt M, Palla K, Kossifologos A, Mitchell D, Lee T. Appropriateness of medication prescribing using the STOPP/START criteria in veterans receiving home-based primary care. Consult Pharm. 2013;28(6):361–9.

28. Egger M, Balmer F, Friedli-Wuthrich H, Muhlemann K. Reduction of urinary catheter use and prescription of antibiotics for asymptomatic bacteriuria in hospitalised patients in internal medicine: before-and-after intervention study. Swiss Med Wkly. 2013;143:w13796.

29. Gillespie U, Alassaad A, Hammarlund-Udenaes M, Morlin C, Henrohn D, Bertilsson M, et al. Effects of pharmacists’ interventions on appropriateness of prescribing and evaluation of the instruments’ (MAI, STOPP and STARTs’) ability to predict hospitalization--analyses from a randomized controlled trial. PLoS One. 2013;8(5):e62401.

30. Gonzales R, Anderer T, McCulloch CE, Maselli JH, Bloom F. J. J, Graf TR, et al. A cluster randomized trial of decision support strategies for reducing antibiotic use in acute bronchitis. JAMA Intern Med. 2015;173(4):267–73.

31. Gupta R, Marshall J, Munoz JC, Kottoor R, Jamal MM, Vega KJ. Decreased acid suppression therapy overuse after education and medication reconciliation. Int J Clin Pract. 2013;67(1):60–5.

32. Jenkins TC, Irwin A, Coombs L, Dealleaume L, Ross SE, Rozwadowski J, et al. Effects of clinical pathways for common outpatient infections on antibiotic prescribing. Am J Med. 2013;126(4):327-335.e12.

33. Johnson LW, Robles J, Hudgins A, Osburn S, Martin D, Thompson A. Management of bronchiolitis in the emergency department: impact of evidence-based guidelines? Pediatrics. 2013;131(SUPPL.1):S103-9.

34. Keith SW, Maio V, Dudash K, Templin M, Del Canale S. A physician-focused intervention to reduce potentially inappropriate medication prescribing in older people: a 3-year, Italian, prospective, proof-of-concept study. Drugs Aging. 2013;30(2):119–27.

35. Litvin CB, Ornstein SM, Wessell AM, Nemeth LS, Nietert PJ. Use of an electronic health record clinical decision support tool to improve antibiotic prescribing for acute respiratory infections: the ABX-TRIP study. J Gen Intern Med. 2013;28(6):810–6.

36. Mainous A. G. 3rd, Lambourne CA, Nietert PJ. Impact of a clinical decision support system on antibiotic prescribing for acute respiratory infections in primary care: quasi-experimental trial. J Am Med Informatics Assoc. 2013;20(2):317–24.

37. Monette J, Monette M, Sourial N, V, Al AC, Wolfson C, et al. Effect of an interdisciplinary educational program on antipsychotic prescribing among residents with dementia in two long-term care centers. J Appl Gerontol. 2013;32(7):833–54.

38. Mousavi M, Dashti-Khavidaki S, Khalili H, Farshchi A, Gatmiri M. Impact of clinical pharmacy services on stress ulcer prophylaxis prescribing and related cost in patients with renal insufficiency. Int J Pharm Pract. 2013;21(4):263–9.

39. Rognstad S, Brekke M, Fetveit A, Dalen I, Stra, J. Prescription peer academic detailing to reduce inappropriate prescribing for older patients: a cluster randomised controlled trial. Br J Gen Pract. 2013;63(613):e554-62.

40. Saifi S, Taylor AJ, Allen J, Hendel R. The use of a learning community and online evaluation of utilization for SPECT myocardial perfusion imaging. Jacc Cardiovasc Imaging. 2013;6(7):823–9.

41. Strother MK, Robert EC, Cobb JG, Pruthi S, Feurer ID. Reduction in the number and associated costs of unindicated dual-phase head CT examinations after a quality improvement initiative. AJR Am J Roentgenol. 2013;201(5):1049–56.

42. Willens HJ, Nelson K, Hendel RC. Appropriate use criteria for stress echocardiography: impact of updated criteria on appropriateness ratings, correlation with pre-authorization guidelines, and effect of temporal trends and an educational initiative on utilization. Jacc Cardiovasc Imaging. 2013;6(3):297–309.

43. Achonduh OA, Mbacham WF, Mangham-Jefferies L, Cundill B, Ch, Ler C, et al. Designing and implementing interventions to change clinicians’ practice in the management of uncomplicated malaria: lessons from Cameroon. Malar J. 2014;13:204.

44. Akenroye AT, Baskin MN, Samnaliev M, Stack AM. Impact of a bronchiolitis guideline on ED resource use and cost: a segmented time-series analysis. Pediatrics. 2014;133(1):e227-34.

45. Bhatia RS, Dudzinski DM, Milford CE, Picard MH, Weiner RB. Educational intervention to reduce inappropriate transthoracic echocardiograms: the need for sustained intervention. Echocardiography. 2014;31(8):916–23.

46. Boonyasiri A, Thamlikitkul V. Effectiveness of multifaceted interventions on rational use of antibiotics for patients with upper respiratory tract infections and acute diarrhea. J Med Assoc Thail. 2014;97:S13-9.

47. Chaves NJ, Ingram RJ, MacIsaac CM, Buising KL. Sticking to minimum standards: implementing antibiotic stewardship in intensive care. Intern Med J. 2014;44(12):1180–7.

48. Fagan M, Lindbaek M, Reiso H, Berild D. A simple intervention to reduce inappropriate ciprofloxacin prescribing in the emergency department. Scand J Infect Dis. 2014;46(7):481–5.

49. Fleet E, Gopal Rao G, Patel B, Cookson B, Charlett A, Bowman C, et al. Impact of implementation of a novel antimicrobial stewardship tool on antibiotic use in nursing homes: a prospective cluster randomized control pilot study. J Antimicrob Chemother. 2014;69(8):2265–73.

50. Kelley D, Aaronson P, Poon E, McCarter YS, Bato B, Jankowski CA. Evaluation of an antimicrobial stewardship approach to minimize overuse of antibiotics in patients with asymptomatic bacteriuria. Infect Control Hosp Epidemiol. 2014;35(2):193–5.

51. Lopatto J, Keith SW, Del Canale S, Templin M, Maio V. Evaluating sustained quality improvements: long-term effectiveness of a physician-focused intervention to reduce potentially inappropriate medication prescribing in an older population. J Clin Pharm Ther. 2014;39(3):266–71.

52. McCullough JM, Zimmerman FJ, Rodriguez HP. Impact of clinical decision support on receipt of antibiotic prescriptions for acute bronchitis and upper respiratory tract infection. J Am Med Informatics Assoc. 2014;21(6):1091–7.

53. Meeker D, Knight TK, Friedberg MW, Linder JA, Goldstein NJ, Fox CR, et al. Nudging guideline-concordant antibiotic prescribing: a randomized clinical trial. JAMA Intern Med. 2014;174(3):425–31.

54. Robson J, Dostal I, Mathur R, Sohanpal R, Hull S, Antoniou S, et al. Improving anticoagulation in atrial fibrillation: observational study in three primary care trusts. Br J Gen Pract. 2014;64(622):e275-81.

55. Szymczak JE, Feemster KA, Zaoutis TE, Gerber JS. Pediatrician perceptions of an outpatient antimicrobial stewardship intervention. Infect Control Hosp Epidemiol. 2014;35:S69-78.

56. Zimmerman S, Sloane PD, Bertr, R., Olsho LE, Beeber A, et al. Successfully reducing antibiotic prescribing in nursing homes. J Am Geriatr Soc. 2014;62(5):907–12.

57. Agee C, Coulter L, Hudson J. Effects of pharmacy resident led education on resident physician prescribing habits associated with stress ulcer prophylaxis in non-intensive care unit patients. Am J Heal Pharm. 2015;72(11):S48-52.

58. Apisarnthanarak A, Lapcharoen P, Vanichkul P, Srisaeng-Ngoen T, Mundy LM. Design and analysis of a pharmacist-enhanced antimicrobial stewardship program in Thailand. Am J Infect Control. 2015;43(9):956–9.

59. Bowblis JR, Lucas JA, Brunt CS. The Effects of Antipsychotic Quality Reporting on Antipsychotic and Psychoactive Medication Use. Health Serv Res. 2015;50(4):1069–87.

60. Buckley MS, Park AS, Anderson CS, Barletta JF, Bikin DS, Gerkin RD, et al. Impact of a clinical pharmacist stress ulcer prophylaxis management program on inappropriate use in hospitalized patients. Am J Med. 2015;128(8):905–13.

61. Cameron M, Jones S, Adedeji O. Antibiotic prophylaxis audit and questionnaire study: Traffic Light Poster improves adherence to protocol in gastrointestinal surgery. Int J Surg. 2015;19:112–5.

62. Choma K, McKeever AE. Cervical cancer screening in adolescents: an evidence-based internet education program for practice improvement among advanced practice nurses. Worldviews Evidence-Based Nurs. 2015;12(1):51–60.

63. Gebhardt BJ, Rajagopalan MS, Gill BS, Heron DE, Rakfal SM, Flickinger JC, et al. Impact of dynamic changes to a bone metastases pathway in a large, integrated, National Cancer Institute-designated comprehensive cancer center network. Pract Radiat Oncol. 2015;5(6):398–405.

64. Giles M, Watts W, O’Brien A, Berenger S, Paul M, McNeil K, et al. Does our bundle stack up! Innovative nurse-led changes for preventing catheter-associated urinary tract infection (CAUTI). Healthc Infect. 2015;20(2):62–71.

65. Ilic D, Bukumiric Z, Jankovic S. Impact of educational intervention on prescribing inappropriate medication to elderly nursing homes residents. Srp Arh Celok Lek. 2015;143(3):174–9.

66. Ip IK, Raja AS, Gupta A, Andruchow J, Sodickson A, Khorasani R. Impact of clinical decision support on head computed tomography use in patients with mild traumatic brain injury in the ED. Am J Emerg Med. 2015;

67. Irfan N, Brooks A, Mithoowani S, Celetti SJ, Main C, Mertz D. A Controlled Quasi-Experimental Study of an Educational Intervention to Reduce the Unnecessary Use of Antimicrobials For Asymptomatic Bacteriuria. PLoS One. 2015;10(7):e0132071.

68. Kashyap M, D’Cruz S, Sachdev A, Tiwari P. Evidence-based information leads to reduction in inappropriate drug prescribing: Results from Indian older inpatients. Int J Risk Saf Med. 2015;27(4):209–17.

69. Kost A, Genao I, Lee JW, Smith SR. Clinical decisions made in primary care clinics before and after choosing wisely^TM^. J Am Board Fam Med. 2015;28(4):471–4.

70. Lester PE, Rios-Rojas L, Islam S, Fazzari MJ, Gomolin IH. Impact of computerized physician order entry alerts on prescribing in older patients. Drugs Aging. 2015;32(3):227–33.

71. Menya D, Platt A, Manji I, Sang E, Wafula R, Ren J, et al. Using pay for performance incentives (P4P) to improve management of suspected malaria fevers in rural Kenya: A cluster randomized controlled trial. BMC Med. 2015;13(1).

72. Mestres C, Agustí A, Puerta L, Barba M. Prescription of potentially inappropriate drugs for geriatric patients in long-term care: Improvement through pharmacist’s intervention. Eur J Hosp Pharm. 2015;22(4):198–201.

73. Njuguna J, Menge D, Nzou J, Chege C. Impact of an Intervention to Minimize Overdiagnosis of Malaria Cases in a Low Risk Kenyan sub-County. J Heal Care Poor Underserved. 2015;26(3):802–10.

74. Orl, O V, Menditto E, Guerriero F, Rotunno R. Effectiveness of the cardiopain initiative in reducing inappropriate nsaid prescriptions in pain therapy among high cardiovascular risk patients: An informative italian survey. Heart Int. 2015;10(1):e20–4.

75. Rayo MF, Kowalczyk N, Liston BW, S, Ers EB, White S, et al. Comparing the Effectiveness of Alerts and Dynamically Annotated Visualizations (DAVs) in Improving Clinical Decision Making. Hum Factors. 2015;57(6):1002–14.

76. Schondelmeyer AC, Simmons JM, Statile AM, Hofacer KE, Smith R, Prine L, et al. Using quality improvement to reduce continuous pulse oximetry use in children with wheezing. Pediatrics. 2015;135(4):e1044-51.

77. Shelton JB, Ochotorena L, Bennett C, Shekelle P, Kwan L, Skolarus T, et al. Reducing PSA-Based Prostate Cancer Screening in Men Aged75 Years and Older with the Use of Highly Specific Computerized Clinical Decision Support. J Gen Intern Med. 2015;30(8):1133–9.

78. So JP, Aleem IS, Tsang DS, Matlow AG, Wright JG, SickKids Surgical Site Infection Task F. Increasing Compliance With an Antibiotic Prophylaxis Guideline to Prevent Pediatric Surgical Site Infection: Before and After Study. Ann Surg. 2015;262(2):403–8.

79. Stevens MB, Hastings SN, Powers J, V, Enberg AE, Echt K V, et al. Enhancing the quality of prescribing practices for older veterans discharged from the emergency department (EQUiPPED): Preliminary results from enhancing quality of prescribing practices for older veterans discharged from the emergency department, a novel. J Am Geriatr Soc. 2015;63(5):1025–9.

80. Strykowski DF, Nielsen AB, Llor C, Siersma V, Bjerrum L. An intervention with access to C-reactive protein rapid test reduces antibiotic overprescribing in acute exacerbations of chronic bronchitis and COPD. Fam Pract. 2015;32(4):395–400.

81. Trautner BW, Grigoryan L, Petersen NJ, Hysong S, Cadena J, Patterson JE, et al. Effectiveness of an Antimicrobial Stewardship Approach for Urinary Catheter-Associated Asymptomatic Bacteriuria. JAMA Intern Med. 2015;175(7):1120–7.

82. Boggan JC, Schulteis RD, Donahue M, Simel DL. Guideline-based decision support has a small, non-sustained effect on transthoracic echocardiography ordering frequency. BMJ Qual Saf. 2015;25(1):57–62.

83. Brooker DJ, Latham I, Evans SC, Jacobson N, Perry W, Bray J, et al. FITS into practice: Translating research into practice in reducing the use of anti-psychotic medication for people with dementia living in care homes. Aging Ment Heal. 2016;20(7).

84. Cossette B, Bergeron J, Ricard G, Ethier JF, Joly-Mischlich T, Levine M, et al. Knowledge Translation Strategy to Reduce the Use of Potentially Inappropriate Medications in Hospitalized Elderly Adults. J Am Geriatr Soc. 2016;64(12):2487–94.

85. Depinet H, von Allmen D, Towbin A, Hornung R, Ho M, Aless, et al. Risk Stratification to Decrease Unnecessary Diagnostic Imaging for Acute Appendicitis. Pediatrics. 2016;138(3).

86. Ellis K, Rubal-Peace G, Chang V, Liang E, Wong N, Campbell S. Antimicrobial Stewardship for a Geriatric Behavioral Health Population. Antibiotics. 2016;5(1):19.

87. Ellis SD, Chen RC, Dusetzina SB, Wheeler SB, Jackson GL, Nielsen ME, et al. Are small reimbursement changes enough to change cancer care? reimbursement variation in prostate cancer treatment. J Oncol Pract. 2016;12(4):e423–36.

88. Elnenaei MO, Campbell SG, Thoni AJ, Lou A, Crocker BD, Nassar BA. An effective utilization management strategy by dual approach of influencing physician ordering and gate keeping. Clin Biochem. 2016;49(3):208–12.

89. Epstein L, Edwards JR, Halpin AL, Preas MA, Blythe D, Harris AD, et al. Evaluation of a novel intervention to reduce unnecessary urine cultures in intensive care units at a Tertiary Care Hospital in Maryland, 2011-2014. Infect Control Hosp Epidemiol. 2016;37(5):606–9.

90. Franchi C, Tettamanti M, Djade CD, Pasina L, Mannucci PM, Onder G, et al. E-learning in order to improve drug prescription for hospitalized older patients: a cluster-randomized controlled study. Br J Clin Pharmacol. 2016;82(1):53–63.

91. Friedman DP, Smith NS. Impact of a Collaborative Radiology Utilization Management Program: Does the Specialty of the Referring Provider Matter? AJR Am J Roentgenol. 2016;207(1):121–5.

92. Gertz ZM, O’Donnell W, Raina A, Balderston JR, Litwack AJ, Goldberg LR. Implementation of a Computerized Order Entry Tool to Reduce the Inappropriate and Unnecessary Use of Cardiac Stress Tests With Imaging in Hospitalized Patients. Am J Cardiol. 2016;118(8):1123–7.

93. Gong CL, Hay JW, Meeker D, Doctor JN. Prescriber preferences for behavioural economics interventions to improve treatment of acute respiratory infections: a discrete choice experiment. BMJ Open. 2016;6(9):e012739.

94. Henao-Villada R, Sossa-Briceno MP, Rodriguez-Martinez CE. Impact of the implementation of an evidence-based guideline on diagnostic testing, management, and clinical outcomes for infants with bronchiolitis. Ther Adv Respir Dis. 2016;10(5):425–34.

95. Holloway KA, Rosella L, Henry D. The Impact of WHO Essential Medicines Policies on Inappropriate Use of Antibiotics. PLoS One. 2016;11(3):e0152020.

96. Ioannou A, Jain A, Kassianos G, Missouris C. Survey of the use of domperidone and the association with QTc prolongation in general practice. Postgrad Med J. 2016;92(1089):390–2.

97. Lasser EC, Pfoh ER, Chang HY, Chan KS, Bailey JC, Kharrazi H, et al. Has Choosing Wisely affected rates of dual-energy X-ray absorptiometry use? Osteoporos Int. 2016;27(7):2311–6.

98. Lesuis N, Hulscher ME, Piek E, Demirel H, van der Laan-Baalbergen N, Meek I, et al. Choosing Wisely in Daily Practice: An Intervention Study on Antinuclear Antibody Testing by Rheumatologists. Arthritis Care Res (Hoboken). 2016;68(4):562–9.

99. Link TL, Townsend ML, Leung E, Kommu S, Vega RY, Hendrix CC. Reducing Inappropriate Antibiotic Prescribing for Adults With Acute Bronchitis in an Urgent Care Setting: A Quality Improvement Initiative. Adv Emerg Nurs J. 2016;38(4):327–35.

100. May L, Franks P, Jerant A, Fenton J. Watchful Waiting Strategy May Reduce Low-Value Diagnostic Testing. J Am Board Fam Med JABFM. 2016;29(6):710–7.

101. McLellan L, Dornan T, Newton P, Williams SD, Lewis P, Steinke D, et al. Pharmacist-led feedback workshops increase appropriate prescribing of antimicrobials. J Antimicrob Chemother. 2016;71(5):1415–25.

102. Morgan S, Morgan A, Kerr R, Tapley A, Magin P. Test ordering by GP trainees: Effects of an educational intervention on attitudes and intended practice. Can Fam Physician. 2016;62(9):733–41.

103. Panesar P, Jones A, Aldous A, Kranzer K, Halpin E, Fifer H, et al. Attitudes and Behaviours to Antimicrobial Prescribing following Introduction of a Smartphone App. PLoS One. 2016;11(4):e0154202.

104. Persell SD, Doctor JN, Friedberg MW, Meeker D, Friesema E, Cooper A, et al. Behavioral interventions to reduce inappropriate antibiotic prescribing: a randomized pilot trial. BMC Infect Dis. 2016;16:373.

105. Petrou P. Failed Attempts to Reduce Inappropriate Laboratory Utilization in an Emergency Department Setting in Cyprus: Lessons Learned. J Emerg Med. 2016;50(3):510–7.

106. Sartelli M, Labricciosa FM, Scoccia L, Bellesi J, Mazzoccanti MR, Scaloni G, et al. Non-Restrictive Antimicrobial Stewardship Program in a General and Emergency Surgery Unit. Surg Infect (Larchmt). 2016;17(4):485–90.

107. Song I, Choi SH, Shin JY. Trends in prescription of pregnancy-contraindicated drugs in Korea, 2007-2011. Regul Toxicol Pharmacol. 2016;75:35–45.

108. Spence SC, McAlister W, Reed B, Zare M, Bingham B, Low J, et al. A Multispecialty Collaboration to Reduce Unnecessary Imaging for Knee Osteoarthritis. J Am Coll Radiol. 2016;13(11):1343–6.

109. Stinnett-Donnelly JM, Stevens PG, Hood VL. Developing a high value care programme from the bottom up: a programme of faculty-resident improvement projects targeting harmful or unnecessary care. BMJ Qual Saf. 2016;25(11):901–8.

110. Thompson I, Lavelle C, Leonard L. An evaluation of the effectiveness of an algorithm intervention in reducing inappropriate faecal samples sent for Clostridium difficile testing. J Infect Prev. 2016;17(6).

111. Vellinga A, Galvin S, Duane S, Callan A, Bennett K, Cormican M, et al. Intervention to improve the quality of antimicrobial prescribing for urinary tract infection: a cluster randomized trial. C Can Med Assoc J. 2016;188(2):108–15.

112. Yeo JM. Antimicrobial stewardship: Improving antibiotic prescribing practice in a respiratory ward. BMJ Qual Improv Reports. 2016;5(1).

113. Barnes GD, Misirliyan S, Kaatz S, Jackson EA, Haymart B, Kline-Rogers E, et al. Barriers and facilitators to reducing frequent laboratory testing for patients who are stable on warfarin: a mixed methods study of de-implementation in five anticoagulation clinics. Implement Sci. 2017;12(1):87.

114. Belfield KD, Kuyumjian AG, Teran R, Amadi M, Blatt M, Bicking K. Impact of A Collaborative Strategy to Reduce the Inappropriate Use of Acid Suppressive Therapy in Non-Intensive Care Unit Patients. Ann Pharmacother. 2017;51(7):577–83.

115. Bond SE, Boutlis CS, Yeo WW, Miyakis S. Impact of an antimicrobial stewardship intervention on appropriateness of prescribing for community-acquired pneumonia in an Australian regional hospital. Intern Med J. 2017;47(5).

116. Bookman K, West D, Ginde A, Wiler J, McIntyre R, Hammes A, et al. Embedded Clinical Decision Support in Electronic Health Record Decreases Use of High-cost Imaging in the Emergency Department: EmbED study. Acad Emerg Med. 2017;24(7):839–45.

117. Frankenthal D, Israeli A, Caraco Y, Lerman Y, Kalendaryev E, Z, et al. Long-Term Outcomes of Medication Intervention Using the Screening Tool of Older Persons Potentially Inappropriate Prescriptions Screening Tool to Alert Doctors to Right Treatment Criteria. J Am Geriatr Soc. 2017;65(2):e33–8.

118. Freer J, Ally T, Brugha R. Impact of Centor scores on determining antibiotic prescribing in children. Int J Health Care Qual Assur. 2016;30(4):319–26.

119. Gillespie P, Clyne B, Raymakers A, Fahey T, Hughes CM, Smith SM. Reducing Potentially Inappropriate Prescribing for Older People in Primary Care: Cost-Effectiveness of the Opti-Script Intervention. Int J Technol Assess Health Care. 2017;33(4):494–503.

120. Hannou S, Voirol P, Pannatier A, Weibel ML, Sadeghipour F, von Gunten A, et al. Pharmacist intervention acceptance for the reduction of potentially inappropriate drug prescribing in acute psychiatry. Int J Clin Pharm. 2017;39(6):1228–36.

121. Hemkens LG, Saccilotto R, Reyes SL, Glinz D, Zumbrunn T, Grolimund O, et al. Personalized Prescription Feedback Using Routinely Collected Data to Reduce Antibiotic Use in Primary Care: A Randomized Clinical Trial. JAMA Intern Med. 2017;177(2):176–83.

122. Ip IK, Lacson R, Hentel K, Malhotra S, Darer J, Langlotz C, et al. JOURNAL CLUB: Predictors of Provider Response to Clinical Decision Support: Lessons Learned From the Medicare Imaging Demonstration. AJR Am J Roentgenol. 2017;208(2):351–7.

123. Jones EB, Swain M, Burdick T. Using clinical decision support to reduce inappropriate imaging: A health care improvement case study. J Clin Outcomes Manag. 2017;24(11).

124. Karthikeyan G, Shirodkar U, Rajivlochan M, Birch S. Appropriateness-based reimbursement of elective invasive coronary procedures in low- and middle-income countries: Preliminary assessment of feasibility in India. Natl Med J India. 2017;30(1):11–4.

125. Kessel L, Erngaard D, Flesner P, Andresen J, Hjortdal J. Do evidence-based guidelines change clinical practice patterns? Acta Opthalmologica. 2017;95(4):337–43.

126. Lacson R, Ip I, Hentel KD, Malhotra S, Balthazar P, Langlotz CP, et al. Medicare Imaging Demonstration: Assessing Attributes of Appropriate Use Criteria and Their Influence on Ordering Behavior. AJR Am J Roentgenol. 2017;208(5):1051–7.

127. Lambl BB, Kaufman N, Kurowski J, O’Neill W, Buckley F. J, Duram M, et al. Does electronic stewardship work? J Am Med Informatics Assoc. 2017;24(5):981–5.

128. Lapic I, Juros GF, Rako I, Rogic D. Changing the electronic request form proves to be an effective tool for optimizing laboratory test utilization in the emergency department. Int J Med Inform. 2017;102:29–34.

129. Linder JA, Meeker D, Fox CR, Friedberg MW, Persell SD, Goldstein NJ, et al. Effects of Behavioral Interventions on Inappropriate Antibiotic Prescribing in Primary Care 12 Months After Stopping Interventions. JAMA. 2017;318(14):1391–2.

130. Luo HL, Fan QZ, Xiao SL, Chen K. Impact of clinical pharmacist interventions on inappropriate prophylactic acid suppressant use in hepatobiliary surgical patients undergoing elective operations. PLoS One. 2017;12(10):15.

131. Nault V, Pepin J, Beaudoin M, Perron J, Moutquin JM, Valiquette L. Sustained impact of a computer-assisted antimicrobial stewardship intervention on antimicrobial use and length of stay. J Antimicrob Chemother. 2017;72(3):933–40.

132. Prochaska MT, Hohmann SF, Modes M, Arora VM. Trends in troponin-only testing for AMI in academic teaching hospitals and the impact of choosing wisely®. J Hosp Med. 2017;12(12):957–62.

133. Roifman I, Austin PC, Qiu F, Wijeysundera HC. Impact of the Publication of Appropriate Use Criteria on Utilization Rates of Myocardial Perfusion Imaging Studies in Ontario, Canada: A Population-Based Study. J Am Heart Assoc. 2017;6(6):5.

134. Ruhl, J., D, Bellone JM, Wilkes E. Implementation and Assessment of an Ambulatory Prescribing Guidance Tool to Improve Patient Safety in the Geriatric Population. Consult Pharm. 2017;32(3):169–74.

135. Sikkens JJ, van Agtmael MA, Peters EJG, Lettinga KD, van der Kuip M, V, et al. Behavioral Approach to Appropriate Antimicrobial Prescribing in Hospitals: The Dutch Unique Method for Antimicrobial Stewardship (DUMAS) Participatory Intervention Study. JAMA Intern Med. 2017;177(8):1130–8.

136. Sim EY, Tan DJA, Abdullah HR. The use of computerized physician order entry with clinical decision support reduces practice variance in ordering preoperative investigations: A retrospective cohort study. Int J Med Inform. 2017;108:29–35.

137. Song I, Shin HN, Shin JY. Decrease in use of contraindicated drugs with automated alerts in children. Pediatr Int. 2017;59(6):720–6.

138. Stocks SJ, Kontopantelis E, Webb RT, Avery AJ, Burns A, Ashcroft DM. Antipsychotic Prescribing to Patients Diagnosed with Dementia Without a Diagnosis of Psychosis in the Context of National Guidance and Drug Safety Warnings: Longitudinal Study in UK General Practice. Drug Saf. 2017;40(8):679–92.

139. V, Erman AJ, Moss JM, Bryan W. E. 3rd, Sloane R, Jackson GL, et al. Evaluating the Impact of Medication Safety Alerts on Prescribing of Potentially Inappropriate Medications for Older Veterans in an Ambulatory Care Setting. J Pharm Pract. 2017;30(1):82–8.

140. Wei XL, Zhang ZT, Walley JD, Hicks JP, Zeng J, Deng SM, et al. Effect of a training and educational intervention for physicians and caregivers on antibiotic prescribing for upper respiratory tract infections in children at primary care facilities in rural China: a cluster-randomised controlled trial. Lancet Glob Heal. 2017;5(12):E1258–67.

141. Weiner SG, Baker O, Poon SJ, Rodgers AF, Garner C, Nelson LS, et al. The Effect of Opioid Prescribing Guidelines on Prescriptions by Emergency Physicians in Ohio. Ann Emerg Med. 2017;70(6):799-808.e1.

142. Yogo N, Shihadeh K, Young H, Calcaterra SL, Knepper BC, Burman WJ, et al. Intervention to Reduce Broad-Spectrum Antibiotics and Treatment Durations Prescribed at the Time of Hospital Discharge: A Novel Stewardship Approach. Infect Control Hosp Epidemiol. 2017;38(5):534–41.

143. Breakell R, Thorndyke B, Clennett J, Harkensee C. Reducing unnecessary chest X-rays, antibiotics and bronchodilators through implementation of the NICE bronchiolitis guideline. Eur J Pediatr. 2018;177(1):47–51.

144. Gong CL, Zangwill KM, Hay JW, Meeker D, Doctor JN. Behavioral Economics Interventions to Improve Outpatient Antibiotic Prescribing for Acute Respiratory Infections: a Cost-Effectiveness Analysis. J Gen Intern Med. 2019;8:8.

145. Graves JM, Fulton-Kehoe D, Jarvik JG, Franklin GM. Impact of an Advanced Imaging Utilization Review Program on Downstream Health Care Utilization and Costs for Low Back Pain. Med Care. 2018;56(6):520–8.

146. Gregg JR, Bhalla RG, Cook JP, Kang C, Dmochowski R, Talbot TR, et al. An Evidence-Based Protocol for Antibiotic Use Prior to Cystoscopy Decreases Antibiotic Use without Impacting Post-Procedural Symptomatic Urinary Tract Infection Rates. J Urol. 2018;199(4):1004–10.

147. Lemiengre MB, Verbakel JY, Colman R, De Burghgraeve T, Buntinx F, Aertgeerts B, et al. Reducing inappropriate antibiotic prescribing for children in primary care: a cluster randomised controlled trial of two interventions. Br J Gen Pract. 2018;68(668):e204–10.

148. Magin P, Tapley A, Morgan S, Davis JS, McElduff P, Yardley L, et al. Reducing early career general practitioners’ antibiotic prescribing for respiratory tract infections: A pragmatic prospective non-randomised controlled trial. Fam Pract. 2018;35(1):53–60.

149. Najjar MF, Sulaiman SAS, Al Jeraisy M, Balubaid H. The impact of a combined intervention program: An educational and clinical pharmacist’s intervention to improve prescribing pattern in hospitalized geriatric patients at King Abdulaziz Medical City in Riyadh, Saudi Arabia. Ther Clin Risk Manag. 2018;14:557–64.

150. Rattanaumpawan P, Chuenchom N, Thamlikitkul V. Individual feedback to reduce inappropriate antimicrobial prescriptions for treating acute upper respiratory infections in an outpatient setting of a Thai university hospital. J Glob Antimicrob Resist. 2018;12:11–4.

151. Tyler A, Krack P, Bakel LA, O’Hara K, Scudamore D, Topoz I, et al. Interventions to Reduce Over-Utilized Tests and Treatments in Bronchiolitis. Pediatrics. 2018;11:11.

152. Clyne B, Smith SM, Hughes CM, Bol, F., Bradley MC, et al. Effectiveness of a Multifaceted Intervention for Potentially Inappropriate Prescribing in Older Patients in Primary Care: A Cluster-Randomized Controlled Trial (OPTI-SCRIPT Study). Ann Fam Med. 2015;13(6):545–53.

153. Clyne B, Smith SM, Hughes CM, Bol, F., Cooper JA, et al. Sustained effectiveness of a multifaceted intervention to reduce potentially inappropriate prescribing in older patients in primary care (OPTI-SCRIPT study). Implement Sci. 2016;11(1):79.

154. Maude RM, Skinner JP, Foureur MJ. Intelligent Structured Intermittent Auscultation (ISIA): evaluation of a decision-making framework for fetal heart monitoring of low-risk women. BMC Pregnancy Childbirth. 2014;14:184.

155. Seetasith A, Holdford D, Shah A, Patterson J. On-label and off-label prescribing patterns of erythropoiesis-stimulating agents in inpatient hospital settings in the US during the period of major regulatory changes. Res Soc Adm Pharm. 2017;13(4):778–88.

156. Tawiah P, Black M, Scott-Walker M, Johnson E, Vaughan CP. Reducing antipsychotic use through culture change: An interdisciplinary effort. Ann Long-Term Care. 2016;24(10):27–32.

157. Tasaka CL, Burg C, VanOsdol SJ, Bekeart L, Anglemyer A, Tsourounis C, et al. An interprofessional approach to reducing the overutilization of stress ulcer prophylaxis in adult medical and surgical intensive care units. Ann Pharmacother. 2014;48(4):462–9.

158. Abdul-Moheeth M, Valencia V, Schaefer S, Brode WM, Nieto K, Moriates C. Improving Healthcare Value: Effectiveness of a Program to Reduce Laboratory Testing for Non-Critically-Ill Patients With COVID-19. J Hosp Med. 2021;16(8):495–8.

159. Abrahamson K, Davila H, Kirk L, Garavito GA, Mueller C. Can a Nursing Home Psychotropic Reduction Project be Successfully Implemented in Assisted Living? J Appl Gerontol. 2021;40(9):1071–9.

160. Adeola M, Azad R, Kassie GM, Shirkey B, Taffet G, Liebl M, et al. Multicomponent Interventions Reduce High-Risk Medications for Delirium in Hospitalized Older Adults. J Am Geriatr Soc. 2018;66(8):1638–45.

161. Aharaz A, Rasmussen JH, McNulty HBØ, Cyron A, Fabricius PK, Bengaard AK, et al. A collaborative deprescribing intervention in a subacute medical outpatient clinic: A pilot randomized controlled trial. Metabolites. 2021 Apr 1;11(4).

162. Ahmed SA, Kumar A, Sethi P, Kapil A, Pandey RM. Original Articles Effectiveness of education and antibiotic control programme at All India Institute of Medical Sciences, New Delhi. Vol. 31, THE NATIONAL MEDICAL JOURNAL OF INDIA. 2018.

163. Akkawi ME, Nik Mohamed MH, Md Aris MA. The impact of a multifaceted intervention to reduce potentially inappropriate prescribing among discharged older adults: A before-and-after study. J Pharm Policy Pract. 2020 Jul 17;13(1).

164. Alcorn S, van der Hoek J, Shaban RZ. Reducing inappropriate third-generation cephalosporin use for community-acquired pneumonia in a small Australian emergency department. Infect Dis Heal. 2018;23(3):163–9.

165. Arizmendez NP, Kotovicz F, Kram JJF, Baumgardner DJ. Multimodal local opioid prescribing intervention outcomes in chronic noncancer pain management. J Am Board Fam Med. 2019;32(4):559–66.

166. Azeez S, Panakkal LM, Meenpidiyil SS, Sulaiman N. Impact of clinical pharmacist intervention in promoting rational antibiotic use in pediatric patients. Res J Pharm Technol. 2020;13(11):5077–82.

167. Balsom C, Pittman N, King R, Kelly D. Impact of a pharmacist-administered deprescribing intervention on nursing home residents: a randomized controlled trial. Int J Clin Pharm. 2020 Aug 1;42(4):1153–67.

168. Bejjanki H, Mramba LK, Beal SG, Radhakrishnan N, Bishnoi R, Shah C, et al. The role of a best practice alert in the electronic medical record in reducing repetitive lab tests. Clin Outcomes Res. 2018;10:611–8.

169. Berg K, Nedved A, Richardson T, Montalbano A, Michael J, Johnson M. Actively Doing Less: Deimplementation of Unnecessary Interventions in Bronchiolitis Care Across Urgent Care, Emergency Department, and Inpatient Settings. Hosp Pediatr. 2020 May 1;10(5):385–91.

170. Biezen R, Buising K, Monaghan T, Bal R, Thursky K, Cheah R, et al. Evaluating the implementation of a pilot quality improvement program to support appropriate antimicrobial prescribing in general practice. Antibiotics. 2021;10(7).

171. Blum MR, Sallevelt BTGM, Spinewine A, O’Mahony D, Moutzouri E, Feller M, et al. Optimizing Therapy to Prevent Avoidable Hospital Admissions in Multimorbid Older Adults (OPERAM): Cluster randomised controlled trial. BMJ. 2021 Jul 13;374.

172. Bolten BC, Bradford JL, White BN, Heath GW, Sizemore JM, White CE. Effects of an automatic discontinuation of antibiotics policy: A novel approach to antimicrobial stewardship. Am J Heal Pharm. 2019 Sep 1;76(Supplement_3):S85–90.

173. Borde JP, Kaier K, Steib-Bauert M, Vach W, Geibel-Zehender A, Busch H, et al. Feasibility and impact of an intensified antibiotic stewardship programme targeting cephalosporin and fluoroquinolone use in a tertiary care university medical center. BMC Infect Dis. 2014;14(1).

174. Bou-Antoun S, Costelloe C, Honeyford K, Mazidi M, Hayhoe BWJ, Holmes A, et al. Age-related decline in antibiotic prescribing for uncomplicated respiratory tract infections in primary care in England following the introduction of a national financial incentive (the Quality Premium) for health commissioners to reduce use of antibiotic. J Antimicrob Chemother. 2018;73(10):2883–92.

175. Bravo-José P, Sáez-Lleó CI, Peris-Martí JF. Deprescribing antipsychotics in long term care patients with dementia. Farm Hosp. 2019;43(4):140–5.

176. Brodaty H, Aerts L, Harrison F, Jessop T, Cations M, Chenoweth L, et al. Antipsychotic Deprescription for Older Adults in Long-term Care: The HALT Study. J Am Med Dir Assoc. 2018 Jul 1;19(7):592-600.e7.

177. Brown CA. Reducing Outpatient Antibiotic Prescribing for Acute Respiratory Infections: A Quasi-Experimental Study. J Dr Nurs Pract. 2018;11(1):3–15.

178. Bruno C, Pearson SA, Daniels B, Buckley NA, Schaffer A, Zoega H. Passing the acid test? Evaluating the impact of national education initiatives to reduce proton pump inhibitor use in Australia. BMJ Qual Saf. 2020 May 1;29(5):365–73.

179. Buckley MS, Agarwal SK, Lansburg JM, Kopp BJ, Erstad BL. Clinical Pharmacist–Led Impact on Inappropriate Albumin Utilization and Associated Costs in General Ward Patients. Ann Pharmacother. 2021 Jan 1;55(1):44–51.

180. Buckley MS, Knutson KD, Agarwal SK, Lansburg JM, Wicks LM, Saggar RC, et al. Clinical Pharmacist–Led Impact on Inappropriate Albumin Use and Costs in the Critically Ill. Ann Pharmacother. 2020 Feb 1;54(2):105–12.

181. Buehrle DJ, Buehrle DJ, Shively NR, Wagener MM, Clancy CJ, Decker BK. Clinical Infectious Diseases Clinical Infectious Diseases ® 2019;XX(XX):1-7 Sustained Reductions in Overall and Unnecessary Antibiotic Prescribing at Primary Care Clinics in a Veterans Affairs Healthcare System Following a Multifaceted Stewardship Intervention. Available from: https://academic.oup.com/cid/advance-article-abstract/doi/10.1093/cid/ciz1180/5669960

182. Cadogan CA, Ryan C, Gormley GJ, Francis JJ, Passmore P, Kerse N, et al. A feasibility study of a theory-based intervention to improve appropriate polypharmacy for older people in primary care. Pilot feasibility Stud. 2018;4(1):23.

183. Calvo LLJ, García Cámara P, Llorente Barrio M, Sierra Gabarda O, Monzón Baez R, Arbonés Mainar JM, et al. Successful deprescribing of proton pump inhibitors with a patient-centered process: the DESPIBP Project. Eur J Clin Pharmacol. 2021;

184. Campbell NL, Holden RJ, Tang Q, Boustani MA, Teal E, Hillstrom J, et al. Multicomponent behavioral intervention to reduce exposure to anticholinergics in primary care older adults. J Am Geriatr Soc. 2021 Jun 1;69(6):1490–9.

185. Campbell NL, Perkins AJ, Khan BA, Gao S, Farber MO, Khan S, et al. Deprescribing in the Pharmacologic Management of Delirium: A Randomized Trial in the Intensive Care Unit. J Am Geriatr Soc. 2019 Apr 1;67(4):695–702.

186. Campins L, Serra-Prat M, Palomera E, Bolibar I, Martínez MÀ, Gallo P. Reduction of pharmaceutical expenditure by a drug appropriateness intervention in polymedicated elderly subjects in Catalonia (Spain). Gac Sanit. 2019;33(2):106–11.

187. Cano-Valderrama O, Cuñarro-López Y, Laiz B, Jiménez-Fuertes M, Torres AJ, Duran-Poveda M. Inappropriate Venous Thromboembolism Prophylaxis in a General Surgery Department: Risk Factors and Improvement with a Simple Educational Program. Indian J Surg. 2020 Oct 1;82(5):855–60.

188. Cardwell K, Smith SM, Clyne B, McCullagh L, Wallace E, Kirke C, et al. Evaluation of the General Practice Pharmacist (GPP) intervention to optimise prescribing in Irish primary care: a non-randomised pilot study. BMJ Open. 2020 Jun 28;10(6):e035087.

189. Cateau D, Ballabeni P, Niquille A. Effects of an interprofessional Quality Circle-Deprescribing Module (QC-DeMo) in Swiss nursing homes: a randomised controlled trial. BMC Geriatr. 2021 Dec 1;21(1).

190. Charra F, Bourne C, Forissier C, Quaglia A, Durand PG, Bergheau F. Quality improvement program of adult urinary tract infection management: Review and impact. Médecine Mal Infect. 2017 Dec 1;47(8):519–25.

191. Chenoweth L, Jessop T, Harrison F, Cations M, Cook J, Brodaty H. Critical Contextual Elements in Facilitating and Achieving Success with a Person-Centred Care Intervention to Support Antipsychotic Deprescribing for Older People in Long-Term Care. Biomed Res Int. 2018;2018.

192. Chiu AS, Freedman-Weiss MR, Jean RA, Cohen E, Yoo PS. No refills: The durable impact of a multifaceted effort by surgical trainees to minimize the prescription of postoperative opioids. Surgery. 2019 Nov 1;166(5):758–63.

193. Chivapricha W, Srinonprasert V, Suansanae T. Impact of Geriatric Pharmacy Specialist Interventions to Reduce Potentially Inappropriate Medication Among Hospitalized Elderly Patients at Medical Wards: A Prospective Quasi-Experimental Study. Drugs - Real World Outcomes. 2021 Mar 1;8(1):39–47.

194. Chowdhury TP, Starr R, Brennan M, Knee A, Ehresman M, Velayutham L, et al. A Quality Improvement Initiative to Improve Medication Management in an Acute Care for Elders Program Through Integration of a Clinical Pharmacist. J Pharm Pract. 2020 Feb 1;33(1):55–62.

195. Clark CM, LaValley SA, Singh R, Mustafa E, Monte S V., Wahler RG. A pharmacist-led pilot program to facilitate deprescribing in a primary care clinic. J Am Pharm Assoc. 2020 Jan 1;60(1):105–11.

196. Coronado-Vázquez V, Gómez-Salgado J, de los Monteros JCE, Ayuso-Murillo D, Ruiz-Frutos C. Shared decision-making in chronic patients with polypharmacy: An interventional study for assessing medication appropriateness. J Clin Med. 2019;8(6).

197. Cossette B, Bruneau MA, Couturier Y, Gilbert S, Boyer D, Ricard J, et al. Optimizing Practices, Use, Care and Services–Antipsychotics (OPUS-AP) in Long-term Care Centers in Québec, Canada: A Strategy for Best Practices. J Am Med Dir Assoc. 2020 Feb 1;21(2):212–9.

198. Couturier Y, Lanneville D, Lane J, Bruneau MA, Morin M, Gilbert S, et al. Implementation conditions leading to the scale-up of an innovation involving the optimal use of antipsychotics in long-term care centers: The Optimizing Practices, Use, Care and Services-Antipsychotics (OPUS-AP) program. Res Soc Adm Pharm. 2022 Mar 1;18(3):2484–8.

199. Craddock K, Molino S, Stranges PM, Suda KJ, Bleasdale SC, Radosta J, et al. The impact of educational interventions on antibiotic prescribing for acute upper respiratory tract infections in the ambulatory care setting: A quasi‐experimental study. JAACP J Am Coll Clin Pharm. 2020;3(3):609–14.

200. Cross AJ, George J, Woodward MC, Le VJ, Elliott RA. Deprescribing potentially inappropriate medications in memory clinic patients (DePIMM): A feasibility study. Res Soc Adm Pharm. 2020 Oct 1;16(10):1392–7.

201. Cummings PL, Alajajian R, May LS, Grant R, Greer H, Sontz J, et al. Utilizing behavioral science to improve antibiotic prescribing in rural urgent care settings. Open Forum Infect Dis. 2020 Jul 1;7(7).

202. Curtin D, Jennings E, Daunt R, Curtin S, Randles M, Gallagher P, et al. Deprescribing in Older People Approaching End of Life: A Randomized Controlled Trial Using STOPPFrail Criteria. J Am Geriatr Soc. 2020 Apr 1;68(4):762–9.

203. Danesh V, Gisi B, Narayan M, Yoder L, Zad O. Fecal Occult Blood Tests: Valuable for Screening, Wasteful for Diagnostics. Clin Nurse Spec. 2019 Jul 1;33(4):191–4.

204. Dehn Lunn A. Reducing inappropriate antibiotic prescribing in upper respiratory tract infection in a primary care setting in Kolkata, India. BMJ Open Qual. 2018;7(4):e000217.

205. Del Giorno R, Ceschi A, Pironi M, Zasa A, Greco A, Gabutti L. Multifaceted intervention to curb in-hospital over-prescription of proton pump inhibitors: A longitudinal multicenter quasi-experimental before-and-after study. Eur J Intern Med. 2018;50:52–9.

206. Del Giorno R, Greco A, Zasa A, Clivio L, Pironi M, Ceschi A, et al. Combining prescription monitoring, benchmarking, and educational interventions to reduce benzodiazepine prescriptions among internal medicine inpatients; a multicenter before and after study in a network of Swiss Public Hospitals. Postgrad Med. 2018;130(7):627–36.

207. Desborough JA, Clark A, Houghton J, Sach T, Shaw V, Kirthisingha V, et al. Clinical and cost effectiveness of a multi-professional medication reviews in care homes (CAREMED). Int J Pharm Pract. 2020;28(6):626–34.

208. Dowling SK, Gjata I, Solbak NM, Weaver CGW, Smart K, Buna R, et al. Group-facilitated audit and feedback to improve bronchiolitis care in the emergency department. Can J Emerg Med. 2020 Sep 1;22(5):678–86.

209. Drees M, Fischer K, Consiglio-Ward L, Caruano J, BCIDP, Chan S, et al. Statewide Antibiotic Stewardship: : An eBrightHealth Choosing Wisely Initiative. Delaware J public Heal. 2019 May 28;5(2):50–8.

210. Edholm K, Kukhareva P, Ciarkowski C, Carr J, Gill D, Rupp A, et al. Decrease in Inpatient Telemetry Utilization Through a System-Wide Electronic Health Record Change and a Multifaceted Hospitalist Intervention. J Hosp Med. 2018 Aug 1;13(8):531–6.

211. Elias RM, Kashiwagi D, Lau C, Hansel SL. An Imaging Stewardship Initiative to Reduce Low-Value Positron Emission Tomography-Computed Tomography Use in Hospitalized Patients. J Healthc Qual. 2020;42(6):e83–91.

212. Ellegård LM, Dietrichson J, Anell A. Can pay‐for‐performance to primary care providers stimulate appropriate use of antibiotics? Health Econ. 2018;27(1):e39–54.

213. Engineer RS, Podolsky SR, Fertel BS, Grover P, Jimenez H, Simon EL, et al. A Pilot Study to Reduce Computed Tomography Utilization for Pediatric Mild Head Injury in the Emergency Department Using a Clinical Decision Support Tool and a Structured Parent Discussion Tool. Pediatr Emerg Care. 2018;37(12):E1670–4.

214. Erard Y, Del Giorno R, Zasa A, De Gottardi S, Della Bruna R, Keller F, et al. A multi-level strategy for a long lasting reduction in unnecessary laboratory testing: A multicenter before and after study in a teaching hospital network. Int J Clin Pract. 2019 Mar 1;73(3).

215. Eskandari F, Abdullah KL, Zainal NZ, Wong LP. The effect of educational intervention on nurses’ knowledge, attitude, intention, practice and incidence rate of physical restraint use. Nurse Educ Pract. 2018;32:52–7.

216. Evrard P, Henrard S, Foulon V, Spinewine A. Benzodiazepine Use and Deprescribing in Belgian Nursing Homes: Results from the COME-ON Study. J Am Geriatr Soc. 2020 Dec 1;68(12):2768–77.

217. Fernández-Liz E, Vivó-Tristante P, Aranzana-Martínez A, Barceló-Colomer ME, Larrosa-Garcia M, del Val García JL, et al. Long-term effectiveness of an intervention to enhance mirabegron use revision and its deprescribing: 36-month follow-up of a quasi-experimental trial in primary care. Curr Med Res Opin. 2021;37(4):703–10.

218. Garay-Bravo C, Peña A, Molina M, Sanfeliu J, Piles P, Blasco P, et al. Application of the STOPP criteria in hospitalised elderly patients to detect and optimise inappropriate psychopharmaceutical prescriptions. Eur Geriatr Med. 2018;9(5):597–602.

219. Gazarin M, Devin B, Tse D, Mulligan E, Naciuk M, Duncan S, et al. Evaluating an inpatient deprescribing initiative at a rural community hospital in Ontario. Can Pharm J. 2020 Jul 1;153(4):224–31.

220. Gibert P, Cabaret M, Moulis M, Bosson JL, Boivin JE, Chanoine S, et al. Optimizing medication use in elderly people in primary care: Impact of STOPP criteria on inappropriate prescriptions. Arch Gerontol Geriatr. 2018;75:16–9.

221. Sara GG, Antoni LA, Ana Cris CL, Oreto RM, Pilar CA, Juan Pablo OB, et al. Deprescribing program in pluripathological elderly patients at a general hospital. Eur J Clin Pharm. 2020;22(3):132–41.

222. Goga JK, Depaolo A, Khushalani S, Walters JK, Roca R, Zisselman M, et al. Lean methodology reduces inappropriate use of antipsychotics for agitation at a psychiatric hospital. Consult Pharm. 2017;32(1):54–62.

223. Greene MT, Fakih MG, Watson SR, Ratz D, Saint S. Reducing Inappropriate Urinary Catheter Use in the Emergency Department: Comparing Two Collaborative Structures. Infect Control Hosp Epidemiol. 2017/12/18. 2018;39(1):77–84.

224. Gulla C, Flo E, Kjome RL, Husebo BS. Deprescribing antihypertensive treatment in nursing home patients and the effect on blood pressure. J Geriatr Cardiol. 2018;15(4):275–83.

225. Gustafsson M, Sjol, Er M, Pfister B, Schneede J, Lovheim H. Effects of Pharmacists’ Interventions on Inappropriate Drug Use and Drug-Related Readmissions in People with Dementia-A Secondary Analysis of a Randomized Controlled Trial. Pharm A J Pharm Educ Pract. 6(1):16.

226. Howard-Anderson JR, Sexton ME, Robichaux C, Wiley Z, Varkey JB, Suchindran S, et al. The impact of an electronic medical record nudge on reducing testing for hospital-onset Clostridioides difficile infection. Infect Control Hosp Epidemiol. 2020/02/10. 2020;41(4):411–7.

227. Hui RL, Chang CC, Niu F, Tang YK, Harano D, Deguzman L, et al. Evaluation of a Pharmacist-Managed Antidiabetic Deprescribing Program in an Integrated Health Care System. Vol. 25, JMCP Journal of Managed Care & Specialty Pharmacy. 2019.

228. Hurmuz MZM, Janus SIM, van Manen JG. Changes in medicine prescription following a medication review in older high-risk patients with polypharmacy. Int J Clin Pharm. 2018;40(2):480–7.

229. James D, Lopez L. Impact of a pharmacist-driven education initiative on treatment of asymptomatic bacteriuria. Am J Heal Pharm. 2019;76:S41–8.

230. Javelot H, Marquis A, Antoine-Bernard E, Grandidier J, Weiner L, Javelot T, et al. Benzodiazepines withdrawal: Initial outcomes and long-term impact on falls in a French nursing home. Pharmacy. 2018;6(2).

231. Jefferson BK, King JE. Impact of the acute care nurse practitioner in reducing the number of unwarranted daily laboratory tests in the intensive care unit. J Am Assoc Nurse Pract. 2018;30(5):285–92.

232. Ji X, Haight SC, Ko JY, Cox S, Barfield WD, Zhang K, et al. Association Between State Policies on Improving Opioid Prescribing in 2 States and Opioid Overdose Rates Among Reproductive-aged Women. Med Care. 2021;59(2).

233. Kato T, Tanaka I, Seyama Y, Sekikawa R, Suzuki S, Nagasawa M, et al. The effectiveness of prescription support and treatment reporting system on the appropriate usage of oral third-generation cephalosporins. J Infect Chemother. 2021;27(3):419–23.

234. Khera S, Abbasi M, Dabravolskaj J, Sadowski CA, Yua H, Chevalier B. Appropriateness of Medications in Older Adults Living With Frailty: Impact of a Pharmacist-Led Structured Medication Review Process in Primary Care. J Prim Care Community Health. 2019;10:2150132719890227–2150132719890227.

235. Kleczka B, Kumar P, Njeru MK, Musiega A, Wekesa P, Rabut G, et al. Using rubber stamps and mobile phones to help understand and change antibiotic prescribing behaviour in private sector primary healthcare clinics in Kenya. BMJ Glob Heal. 2019 Sep 1;4(5):e001422.

236. Kouladjian O’Donnell L, Gnjidic D, Chen TF, Hilmer SN. Integration of an electronic Drug Burden Index risk assessment tool into Home Medicines Reviews: deprescribing anticholinergic and sedative medications. Ther Adv Drug Saf. 2019 Jan;10:204209861983247.

237. Kullgren JT, Krupka E, Schachter A, Linden A, Miller J, Acharya Y, et al. Precommitting to choose wisely about low-value services: A stepped wedge cluster randomised trial. BMJ Qual Saf. 2018;27(5):355–64.

238. Laan BJ, Maaskant JM, Spijkerman IJB, Borgert MJ, Godfried MH, Pasmooij BC, et al. De-implementation strategy to reduce inappropriate use of intravenous and urinary catheters (RICAT): a multicentre, prospective, interrupted time-series and before and after study. Lancet Infect Dis. 2020 Jul 1;20(7):864–72.

239. Lee B, Mafi J, Patel MK, Sorensen A, Vangala S, Wei E, et al. Quality improvement time-saving intervention to increase use of a clinical decision support tool to reduce low-value diagnostic imaging in a safety net health system. BMJ Open Qual. 2021 Feb 1;10(1):e001076.

240. Lee C, Phillips C, Vanstone JR. Educational intervention to reduce treatment of asymptomatic bacteriuria in long-term care. BMJ Open Qual. 2018 Dec 1;7(4):e000483.

241. Leslie T, Rowland M, Mikhail A, Cundill B, Willey B, Alokozai A, et al. Use of malaria rapid diagnostic tests by community health workers in Afghanistan: cluster randomised trial. BMC Med. 2017;15:1–11.

242. Lewis PO, Lundberg TS, Tharp JL, Runnels CW. Implementation of Global Strategies to Prevent Hospital-Onset Clostridium difficile Infection: Targeting Proton Pump Inhibitors and Probiotics. Ann Pharmacother. 2017;51(10):848–54.

243. Li H, Yan S, Li D, Gong Y, Lu Z, Yin X. Trends and patterns of outpatient and inpatient antibiotic use in China’s hospitals: data from the Center for Antibacterial Surveillance, 2012–16. J Antimicrob Chemother. 2019 Jun 1;74(6):1731–40.

244. Lin D, Eke C, Cai C, Thrift AP, Shukla R. Decreasing Overall and Inappropriate Proton Pump Inhibitor Use: Perspective From a Large Safety-Net Healthcare System. Clin Gastroenterol Hepatol. 2020;18(4):763-766.e2.

245. Lin K, Zahlanie Y, Ortwine JK, Mang NS, Wei W, Brown LS, et al. Decreased Outpatient Fluoroquinolone Prescribing Using a Multimodal Antimicrobial Stewardship Initiative. Open Forum Infect Dis. 2020 Jun 1;7(6):ofaa182.

246. Low CE, Sanchez Pellecer DE, Santivasi WL, Thompson VH, Elwood T, Davidson AJ, et al. Deprescribing in Hospice Patients: Discontinuing Aspirin, Multivitamins, and Statins. Mayo Clin Proc Innov Qual Outcomes. 2021 Aug 1;5(4):721–6.

247. Luo H, Fan Q, Xiao S, Chen K. Changes in proton pump inhibitor prescribing trend over the past decade and pharmacists’ effect on prescribing practice at a tertiary hospital. BMC Health Serv Res. 2018;18(1):537.

248. Mafi JN, Godoy-Travieso P, Wei E, Anders M, Amaya R, Carrillo CA, et al. Evaluation of an Intervention to Reduce Low-Value Preoperative Care for Patients Undergoing Cataract Surgery at a Safety-Net Health System. JAMA Intern Med. 2019 May 1;179(5):648–57.

249. Markogiannakis A, Korantanis K, Gamaletsou MN, Samarkos M, Psichogiou M, Daikos G, et al. Impact of a non-compulsory antifungal stewardship program on overuse and misuse of antifungal agents in a tertiary care hospital. Int J Antimicrob Agents. 2021 Jan 1;57(1):106255.

250. Martin P, Tamblyn R, Benedetti A, Ahmed S, Tannenbaum C. Effect of a Pharmacist-Led Educational Intervention on Inappropriate Medication Prescriptions in Older Adults: The D-PRESCRIBE Randomized Clinical Trial. JAMA. 2018 Nov 13;320(18):1889–98.

251. Masood U, Sharma A, Bhatti Z, Carroll J, Bhardwaj A, Sivalingam D, et al. A Successful Pharmacist-Based Quality Initiative to Reduce Inappropriate Stress Ulcer Prophylaxis Use in an Academic Medical Intensive Care Unit. Inq. 2018;55:1–5.

252. May A, Hester A, Quairoli K, Wong JR, Kandiah S. Impact of Clinical Decision Support on Azithromycin Prescribing in Primary Care Clinics. J Gen Intern Med. 2021;36(8):2267–73.

253. McDonald EG, Wu PE, Rashidi B, Forster AJ, Huang A, Pilote L, et al. The MedSafer Study: A Controlled Trial of an Electronic Decision Support Tool for Deprescribing in Acute Care. J Am Geriatr Soc. 2019 Sep 1;67(9):1843–50.

254. Mecca MC, Thomas JM, Niehoff KM, Hyson A, Jeffery SM, Sellinger J, et al. Assessing an Interprofessional Polypharmacy and Deprescribing Educational Intervention for Primary Care Post-graduate Trainees: a Quantitative and Qualitative Evaluation. J Gen Intern Med. 2019;34(7):1220–7.

255. Mitchell DA, Panchisin T, Seckel MA. Reducing Use of Restraints in Intensive Care Units: A Quality Improvement Project. Crit Care Nurse. 2018;38(4):e8–16.

256. Morgan T, Wu J, Ovchinikova L, Lindner R, Blogg S, Moorin R. A national intervention to reduce imaging for low back pain by general practitioners: a retrospective economic program evaluation using Medicare Benefits Schedule data. BMC Health Serv Res. 2019;19(1):983.

257. Mozafarihashjin M, Leis JA, Mieusement LMD, McCreight L, Poutanen S, Shrivastava A, et al. Safety, effectiveness and sustainability of a laboratory intervention to de-adopt culture of midstream urine samples among hospitalized patients. Infect Control Hosp Epidemiol. 2021;42(1):43–50.

258. Musuuza JS, Fong E, Lata P, Willenborg K, Knobloch MJ, Hoernke MJ, et al. Feasibility of a pharmacy-led intervention to de-implement non-guideline-concordant proton pump inhibitor use. Implement Sci Commun. 2021;2(1):59.

259. Naimer MS, Munro J, Singh S, Permaul JA. Improving Family Medicine Residents’ Opioid Prescribing: A Nurse Practitioner-Led Model. J nurse Pract. 2019;15(9):661–5.

260. Narayanan P, Knoph K, Chen NX, McCoy CP, Devalapalli AP, Schoonover K, et al. Decreasing treatment of asymptomatic bacteriuria: An interprofessional approach to antibiotic stewardship. J Clin Outcomes Manag. 2019;26(4):169–74.

261. Navy HJ, Weffald L, Delate T, Patel RJ, Dugan JP. Clinical pharmacist intervention to engage older adults in reducing use of alprazolam. Consult Pharm. 2018;33(12):711–22.

262. Orji CC, Brown CM, Hoverman JR, Richards KM, Garey J, He B. Impact of a G-CSF Policy to Reduce Low-Value Care on Guideline Adherence and Mortality. JCO Oncol Pract. 2021 Apr 14;17(11):e1830–6.

263. Peñalva G, Fernández-Urrusuno R, Turmo JM, Hernández-Soto R, Pajares I, Carrión L, et al. Long-term impact of an educational antimicrobial stewardship programme in primary care on infections caused by extended-spectrum β-lactamase-producing Escherichia coli in the community: an interrupted time-series analysis. Lancet Infect Dis. 2020;20(2):199–207.

264. Pugel S, Stallworth JL, Pugh LB, Terrell C, Bailey Z, Gramling T, et al. Choosing Wisely in Georgia: A Quality Improvement Initiative in 25 Adult Ambulatory Medicine Offices. Jt Comm J Qual Patient Saf. 2018 Dec 1;44(12):699–707.

265. Raina A, Ardagh M, Loring B. What influences clinicians to choose wisely? N Z Med J. 2019;132(1502):16–24.

266. Rautemaa-Richardson R, Rautemaa V, Al-Wathiqi F, Moore CB, Craig L, Felton TW, et al. Impact of a diagnostics-driven antifungal stewardship programme in a UK tertiary referral teaching hospital. J Antimicrob Chemother. 2018 Dec 1;73(12):3488–95.

267. Rezaii PG, Fredericks N, Lincoln CM, Hom J, Willis M, Burleson J, et al. Assessment of the Radiology Support, Communication and Alignment Network to Reduce Medical Imaging Overutilization: A Multipractice Cohort Study. J Am Coll Radiol. 2020 May 1;17(5):597–605.

268. Ruiz-Millo O, Climente-Martí M, Navarro-Sanz JR. Improvement on prescribing appropriateness after implementing an interdisciplinary pharmacotherapy quality programme in a long-term care hospital. Eur J Hosp Pharm. 2018;25(5):267–73.

269. Sacarny A, Barnett ML, Le J, Tetkoski F, Yokum D, Agrawal S. Effect of Peer Comparison Letters for High-Volume Primary Care Prescribers of Quetiapine in Older and Disabled Adults: A Randomized Clinical Trial. JAMA Psychiatry. 2018 Oct 1;75(10):1003–11.

270. San-José A, Pérez-Bocanegra C, Agustí A, Laorden H, Gost J, Vidal X, et al. Integrated health intervention on polypharmacy and inappropriate prescribing in elderly people with multimorbidity: Results at the end of the intervention and at 6 months after the intervention. Med Clin (Barc). 2021 Mar 26;156(6):263–9.

271. Sanyal C, Turner JP, Martin P, Tannenbaum C. Cost-Effectiveness of Pharmacist-Led Deprescribing of NSAIDs in Community-Dwelling Older Adults. J Am Geriatr Soc. 2020 May 1;68(5):1090–7.

272. Sanz-Tamargo G, García-Cases S, Navarro A, Lumbreras B. Adaptation of a deprescription intervention to the medication management of older people living in long-term care facilities. Expert Opin Drug Saf. 2019 Nov 2;18(11):1091–8.

273. Schaffer AL, Buckley NA, Cairns R, Pearson S. Comparison of Prescribing Patterns Before and After Implementation of a National Policy to Reduce Inappropriate Alprazolam Prescribing in Australia. JAMA Netw Open. 2019 Sep 18;2(9):e1911590–e1911590.

274. Schapira M, Calabró P, Montero-Odasso M, Osman A, Guajardo ME, Martínez B, et al. A multifactorial intervention to lower potentially inappropriate medication use in older adults in Argentina. Aging Clin Exp Res. 2021;33(12):3313–20.

275. Schäfer I, Kaduszkiewicz H, Mellert C, Löffler C, Mortsiefer A, Ernst A, et al. Narrative medicine-based intervention in primary care to reduce polypharmacy: results from the cluster-randomised controlled trial MultiCare AGENDA. BMJ Open. 2018;8(1):e017653–e017653.

276. Shilpa HSS, Kumar NN, Maheswari E, Virupaksha HS, Subeesh V, Saraswathy GR, et al. Deprescribing of benzodiazepines and Z-drugs amongst the psychiatric patients of a tertiary care Hospital. Asian J Psychiatr. 2019;44:189–94.

277. Soerensen AL, Lisby M, Nielsen LP, Poulsen BK, Mainz J. Improving Medication Safety in Psychiatry – A Controlled Intervention Study of Nurse Involvement in Avoidance of Potentially Inappropriate Prescriptions. Basic Clin Pharmacol Toxicol. 2018 Aug 1;123(2):174–81.

278. Soon J, Traeger AC, Elshaug AG, Cvejic E, Maher CG, Doust JA, et al. Effect of two behavioural ‘nudging’ interventions on management decisions for low back pain: a randomised vignette-based study in general practitioners. BMJ Qual &amp;amp; Saf. 2019 Jul 1;28(7):547.

279. Taher J, Beriault DR, Yip D, Tahir S, Hicks LK, Gilmour JA. Reducing free thyroid hormone testing through multiple Plan-Do-Study-Act cycles. Clin Biochem. 2020 Jul 1;81:41–6.

280. Tang Y, Liu C, Zhang Z, Zhang X. Effects of prescription restrictive interventions on antibiotic procurement in primary care settings: a controlled interrupted time series study in China. Cost Eff Resour Alloc. 2018;16(1):1.

281. Tejedor-Sojo J, Chan KN, Bailey M, Williams A, Killgore M, Gillard L, et al. Improving Bronchiolitis Care in Outpatient Settings Across a Health Care System. Pediatr Emerg Care. 2019;35(11).

282. Thompson MP, Graetz I, McKillop CN, Grubb PH, Waters TM. Evaluation of a Tennessee statewide initiative to reduce early elective deliveries using quasi-experimental methods. BMC Health Serv Res. 2019;19(1):208.

283. Turner JP, Richard C, Lussier M-T, Lavoie M-E, Farrell B, Roberge D, et al. Deprescribing conversations: a closer look at prescriber–patient communication. Ther Adv drug Saf. 2018;9(12):687–98.

284. Van der Linden L, Hias J, Dreessen L, Milisen K, Flamaing J, Spriet I, et al. Medication review versus usual care to improve drug therapies in older inpatients not admitted to geriatric wards: a quasi-experimental study (RASP-IGCT). BMC Geriatr. 2018;18(1):155.

285. Van Der Spek K, Koopmans RT, Smalbrugge M, Nelissen-Vrancken MH, Wetzels RB, Smeets CH, et al. The effect of biannual medication reviews on the appropriateness of psychotropic drug use for neuropsychiatric symptoms in patients with dementia: A randomised controlled trial. Age Ageing. 2018;47(3):430–7.

286. van Uden RCAE, van den Broek MPH, Houtenbos I, Jaspers TCC, Harmsze AM, Kingma HJ, et al. Unintentional guideline deviations in hospitalized patients with two or more antithrombotic agents: an intervention study. Eur J Clin Pharmacol. 2021;77(12):1919–26.

287. Vugt S, Schepper E, Delft S, Zuithoff N, Wit N, Bindels P. Reducing vitamin test ordering in primary care; the effectiveness of professional and patient oriented strategies in a Cluster randomized Intervention Study. BJGP Open. 2021 Aug 18;5:BJGPO.2021.0113.

288. Vandenberg AE, Echt K V, Kemp L, McGwin G, Perkins MM, Mirk AK. Academic Detailing with Provider Audit and Feedback Improve Prescribing Quality for Older Veterans. J Am Geriatr Soc. 2018;66(3):621–7.

289. Vrijkorte E, de Vries J, Schaafsma R, Wymenga M, Oude Munnink T. Optimising pharmacotherapy in older cancer patients with polypharmacy. Eur J Cancer Care (Engl). 2020 Jan 1;29(1):e13185.

290. Vu TX, Huong QBT. The effect of the pharmacist’s intervention on potentially inappropriate medication prescription in older adults in a Vietnamese hospital. Pharm Sci Asia. 2019;46(1):54–61.

291. Walker AJ, Curtis HJ, Goldacre B. Impact of Chief Medical Officer activity on prescribing of antibiotics in England: an interrupted time series analysis. J Antimicrob Chemother. 2019;74(4):1133–6.

292. Wang KY, Yen CJ, Chen M, Variyam D, Acosta TU, Reed B, et al. Reducing Inappropriate Lumbar Spine MRI for Low Back Pain: Radiology Support, Communication and Alignment Network. J Am Coll Radiol. 2018;15(1):116–22.

293. Watnick CS, Arnold DH, Latuska R, O’Connor M, Johnson DP. Successful Chest Radiograph Reduction by Using Quality Improvement Methodology for Children With Asthma. Pediatrics. 2018 Aug 1;142(2):e20174003.

294. Wauters M, Elseviers M, Vander Stichele R, Dilles T, Thienpont G, Christiaens T. Efficacy, feasibility and acceptability of the OptiMEDs tool for multidisciplinary medication review in nursing homes. Arch Gerontol Geriatr. 2021;95.

295. Weeks WB, Mishra MK, Curto D, Petersen CL, Cano P, Hswen Y, et al. Comparing Three Methods for Reducing Psychotropic Use in Older Demented Spanish Care Home Residents. J Am Geriatr Soc. 2019 Jul 1;67(7):1444–53.

296. Westaway K, Frank O, Shute R, Pall D, Moffat A, LeBlanc V, et al. Gathering tips from carers to support people with dementia: an adaptation of the TOP 5 program for community use. Int J Evid Based Healthc. 2018;16(2):128–35.

297. Whitman A, DeGregory K, Morris A, Mohile S, Ramsdale E. Pharmacist-led medication assessment and deprescribing intervention for older adults with cancer and polypharmacy: a pilot study. Support Care Cancer. 2018;26(12):4105–13.

298. Whitty R, Porter S, Battu K, Bhatt P, Koo E, Kalocsai C, et al. A pilot study of a Medication Rationalization (MERA) intervention. C open. 2AD;6(1):E87–94.

299. Wilchesky M, Mueller G, Morin M, Marcotte M, Voyer P, Aubin M, et al. The OptimaMed intervention to reduce inappropriate medications in nursing home residents with severe dementia: results from a quasi-experimental feasibility pilot study. BMC Geriatr. 2018 Sep 4;18(1):204.

300. Wilson A, Kelly M, Henderson E, Mcbain L, Jayathissa S, Loring B. Reducing inappropriate urine testing at Hutt Valley District Health Board using Choosing Wisely principles. N Z Med J. 2019 Jan 18;132:11–20.

301. Wilson MG, Lee TC, Hass A, Tannenbaum C, McDonald EG. EMPOWERing Hospitalized Older Adults to Deprescribe Sedative Hypnotics: A Pilot Study. J Am Geriatr Soc. 2018;66(6):1186–9.

302. Wintemute K, Greiver M, McIsaac W, Del Giudice ME, Sullivan F, Aliarzadeh B, et al. Choosing Wisely Canada campaign associated with less overuse of thyroid testing: Retrospective parallel cohort study. Can Fam Physician. 2019 Nov;65(11):e487–96.

303. Wright SM, Hedin SC, McConnell M, Burke B V, Watts SA, Leslie DM, et al. Using Shared Decision-Making to Address Possible Overtreatment in Patients at High Risk for Hypoglycemia: The Veterans Health Administration’s Choosing Wisely Hypoglycemia Safety Initiative. Clin diabetes. 2018;36(2):120–7.

304. Wu J, Taylor D, Ovchinikova L, Heaney A, Morgan T, Dartnell J, et al. Relationship between antimicrobial-resistance programs and antibiotic dispensing for upper respiratory tract infection: An analysis of Australian data between 2004 and 2015. J Int Med Res. 2018;46(4):1326–38.

305. Wu J, Dickinson S, Elgebaly Z, Blogg S, Heaney A, Soo Y, et al. Impact of NPS MedicineWise general practitioner education programs and Choosing Wisely Australia recommendations on prescribing of proton pump inhibitors in Australia. BMC Fam Pract. 2020 May 9;21(1):85.

306. Lin M, Xin C, Li G-H, Dong Z. The impact of pharmaceutical interventions on the rational use of proton pump inhibitors in a Chinese hospital. Patient Prefer Adherence. 2020;21.

307. Yadav K, Meeker D, Mistry RD, Doctor JN, Fleming-Dutra KE, Fleischman RJ, et al. A Multifaceted Intervention Improves Prescribing for Acute Respiratory Infection for Adults and Children in Emergency Department and Urgent Care Settings. Acad Emerg Med. 2019 Jul 1;26(7):719–31.

308. Zhang Y, Yang H, Kong J, Liu L, Ran L, Zhang X, et al. Impact of interventions targeting the inappropriate use of proton-pump inhibitors by clinical pharmacists in a hepatobiliary surgery department. J Clin Pharm Ther. 2021 Feb 1;46(1):149–57.

309. Zhen L, Jin C, Xu HN. The impact of prescriptions audit and feedback for antibiotic use in rural clinics: Interrupted time series with segmented regression analysis 11 Medical and Health Sciences 1117 Public Health and Health Services. BMC Health Serv Res. 2018 Oct 16;18(1).

310. Trumbo SP, Iams WT, Limper HM, Goggins K, Gibson J, Oliver L, et al. Deimplementation of Routine Chest X-rays in Adult Intensive Care Units. J Hosp Med. 2019 Feb 1;14(2):83–9.
